# Functional and structural characterization of F_1_ ‐ATPase with common ancestral core domains in stator ring

**DOI:** 10.1002/pro.70345

**Published:** 2025-10-23

**Authors:** Aya K. Suzuki, Ryutaro Furukawa, Meghna Sobti, Simon H. J. Brown, Alastair G. Stewart, Satoshi Akanuma, Hiroshi Ueno, Hiroyuki Noji

**Affiliations:** ^1^ Department of Life Sciences, Graduate School of Arts and Sciences The University of Tokyo Tokyo Japan; ^2^ Department of Applied Chemistry, Graduate School of Engineering The University of Tokyo Tokyo Japan; ^3^ Molecular, Structural and Computational Biology Division The Victor Chang Cardiac Research Institute New South Wales Australia; ^4^ St Vincent's Clinical School, Faculty of Medicine UNSW Sydney New South Wales Australia; ^5^ School of Science, Molecular Horizons, and the Australian Research Council Centre for Cryo‐Electron Microscopy of Membrane Proteins University of Wollongong Wollongong New South Wales Australia; ^6^ Faculty of Human Sciences, Waseda University Saitama Japan; ^7^ Research Institute of Planetary Health (RIPH), The University of Tokyo Tokyo Japan

**Keywords:** ancestral sequencereconstruction, ATPase, cryo‐electronmicroscopy, evolution, F‐type ATPase, protein engineering, type III secretionsystem (T3SS) ATPase, V‐type ATPase

## Abstract

Extant F_1_‐ATPases exhibit diverse rotational stepping behaviors—3‐, 6‐, or 9‐step cycles—yet the evolutionary origin of these patterns remains unclear. Here, we used ancestral sequence reconstruction to infer the catalytic β and non‐catalytic α subunits of a putative ancestral F_1_‐ATPase. We then fused their functionally critical domains into the thermostable F_1_ from *Bacillus* PS3, yielding a stable chimeric enzyme. Cryo‐EM revealed two distinct conformational states—binding and catalytic dwell states—separated by a ~34° rotation of the γ subunit, suggesting a fundamental six‐step mechanism akin to that of extant six‐stepping F_1_‐ATPases. Single‐molecule rotation assays with ATP and the slowly hydrolyzed ATP analog ATPγS demonstrated that the chimeric motor is intrinsically a six‐stepper, pausing at binding and catalytic dwell positions separated by 32.1°, although the binding dwell is significantly prolonged by an unknown mechanism. These findings indicate that F_1_‐ATPase was originally a six‐stepper and diversified into 3‐, 6‐ and 9‐step forms in evolutionary adaptation. Based on these results, we discuss plausible features of the entire F_o_F_1_ complex, along with potential physiological contexts in the last universal common ancestor and related lineages.

## 1 INTRODUCTION

F‐type ATP synthase catalyzes the terminal reaction of oxidative phosphorylation—namely, the synthesis of ATP from ADP and inorganic phosphate (P_i_) driven by proton translocation down a proton motive force (pmf) across biomembranes. This enzyme is among the most ubiquitous in nature, found in the plasma membranes of prokaryotic cells, the thylakoid membranes of chloroplasts, and the inner membranes of mitochondria. Recent comparative genomic analyses have revealed that cells of the last universal common ancestor (LUCA) already possessed F‐type or relevant type ATP synthase (Mahendrarajah et al., 2023). These findings provide significant insights into the metabolic capabilities and ecological contexts of LUCA and its descendants, including the last bacterial common ancestor (LBCA) and the last archaeal common ancestor (LACA) (Mahendrarajah et al., 2023; Moody et al., 2024).

F‐type ATP synthase is composed of two rotary motors termed F_o_ and F_1_. F_o_ is the membrane‐embedded portion, where the *c*‐oligomer ring (*c*‐ring) rotates against the *ab*_2_ stator complex during proton translocation across the membrane. F_1_ is the membrane‐protruding portion and rotates the inner rotor complex against the catalytic stator ring during ATP hydrolysis. F_o_ and F_1_ are connected by the rotor complex and the peripheral stalk, so as to enable the interconversion of pmf and the free energy of ATP hydrolysis. Under ATP‐synthesizing conditions—when the pmf is sufficient and the rotational torque of F_o_ exceeds that of F_1_—F_o_ drives the reverse rotation of F_1_ (opposite to the ATP‐hydrolyzing direction), thereby inducing ATP synthesis on the catalytic stator ring (Noji et al., 2017; Weiss et al., 2016). Conversely, when the torque of F_1_ exceeds that of F_o_ and in the absence of regulatory elements, F_1_ rotates the *c*‐ring in F_o_, forcing F_o_ to pump protons in the reverse direction and thus generate pmf. In this way, F_o_F_1_ interconverts the pmf and the chemical potential of ATP hydrolysis via mechanical rotation.

The minimal subunit composition of F_1_, acting as the ATP‐driven motor, is α_3_β_3_γ_1_, with the γ subunit embedded inside a hetero‐hexameric stator ring consisting of alternating α and β subunits. The α_3_β_3_ stator ring has three catalytic sites, each located at the α–β interface. Owing to the structural asymmetry of α and β, there are two distinct types of interfaces in the α_3_β_3_ ring. One type—the α–β interface—contains the catalytic site, whereas the other type—the β–α interface—also binds ATP but does not hydrolyze it. Because most catalytically important residues reside on the β subunit, it is termed the “catalytic subunit.” Conversely, the “non‐catalytic” ATP‐binding site at the β–α interface is formed mainly by the α subunit residues, so the α subunit is termed the “non‐catalytic subunit.” During catalysis, the three β subunits undergo conformational changes in a coordinated manner, resulting in the unidirectional rotation of the γ subunit (Uchihashi et al., 2011).

The rotary catalysis of F_1_ has been extensively studied via single‐molecule rotation assays of F_1_‐ATPase derived from the thermophilic bacterium *Bacillus* PS3 (hereafter TF_1_) due to stability and the ease of handling (Noji et al., 2017). In consistent with the pseudo‐threefold symmetry of F_1_, the basic step size of rotation is 120°. This 120° step was subsequently resolved into two discrete substeps of 80° and 40°, each initiated after ATP binding and hydrolysis, respectively. Accordingly, the dwell states prior to the 80° and 40° substeps are referred to as the “binding dwell” and the “catalytic dwell,” respectively (Noji & Ueno, 2022). Later studies showed that F_1_ releases P_i_ from a β subunit in the catalytic dwell, but distinct from the one engaged in catalysis. It was also reported that F_1_ pauses at binding dwell angle during temperature‐sensitive reaction intermediate (Enoki et al., 2009). Note that the detailed statistical analysis of the catalytic dwell revealed that ATP hydrolysis induces rotation during the dwell phase (Li et al., 2015). However, the angular displacement upon hydrolysis during dwell phase is subtle and within the angle distribution of the catalytic dwell (typically ±10° − 20°). Thus, TF_1_ is principally a “6‐stepper” motor, making three binding and three catalytic dwells per turn.

Similar reaction schemes with six steps per turn have been reported for F_1_‐ATPases derived from *Escherichia coli* (EF_1_) and yeast mitochondria (yMF_1_) (Steel et al., 2015; Yanagisawa et al., 2024). However, recent studies have revealed variations in the number of substeps. F_1_‐ATPase from human and bovine mitochondrial F_1_ (*h*MF_1_, *b*MF_1_) exhibits an additional pause—referred to as the “short dwell”—alongside the binding and catalytic dwells, resulting in nine steps per turn (Kobayashi et al., 2020). In contrast, F_1_‐ATPase from *Paracoccus denitrificans* (PdF_1_) shows only three steps per turn under all tested conditions, despite its close evolutionary relationship to mitochondria (*Paracoccus* is an α‐proteobacterium from which the mitochondrial ancestor is thought to have diverged) (Zarco‐Zavala et al., 2020). Thus, while most F_1_‐ATPases pause six times per turn (“6‐steppers”), mammalian mitochondrial F_1_ is a “9‐stepper,” and PdF_1_ is a “3‐stepper.” Notably, the number of steps per turn correlates with the number of the *c‐*subunits in the *c*‐ring: six‐steppers typically pair F_o_ with a *c*_10_‐ring, nine‐steppers have a *c*_8_‐ring, and three‐steppers have a *c*_12_‐ring. This trend suggests that F_1_ with more steps per turn has a *c*‐ring containing fewer *c* subunits, implying certain mechanistic or physiological constraints on the total number of steps in the entire F_o_F_1_ complex (Noji et al., 2020).

To investigate which subunit determines the stepping pattern of F_1_, we previously constructed various hybrid F_1_‐ATPases whose subunits originated from different species—TF_1_, *b*MF_1_, and PdF_1_ (Watanabe et al., 2023). Analysis of these hybrids showed that rotational speed principally depends on the origin of the β subunit, as expected. However, we did not identify a single comprehensive rule governing the number of steps for all hybrids. We did find one conditional rule: whenever a hybrid F_1_ contains a subunit from PdF_1_, it consistently exhibits three‐step rotation per turn, just like PdF_1_, regardless of the origins of the other subunits. Hence, although fundamental features—such as the 120° step coupled to a single ATP hydrolysis turnover and the rotation direction—are broadly conserved across species, the number and size of substeps vary. This naturally raises the question: What was the original stepping pattern of F_1_‐ATPase? In other words, how did the common ancestral F_1_‐ATPase rotate?

**FIGURE 1:**
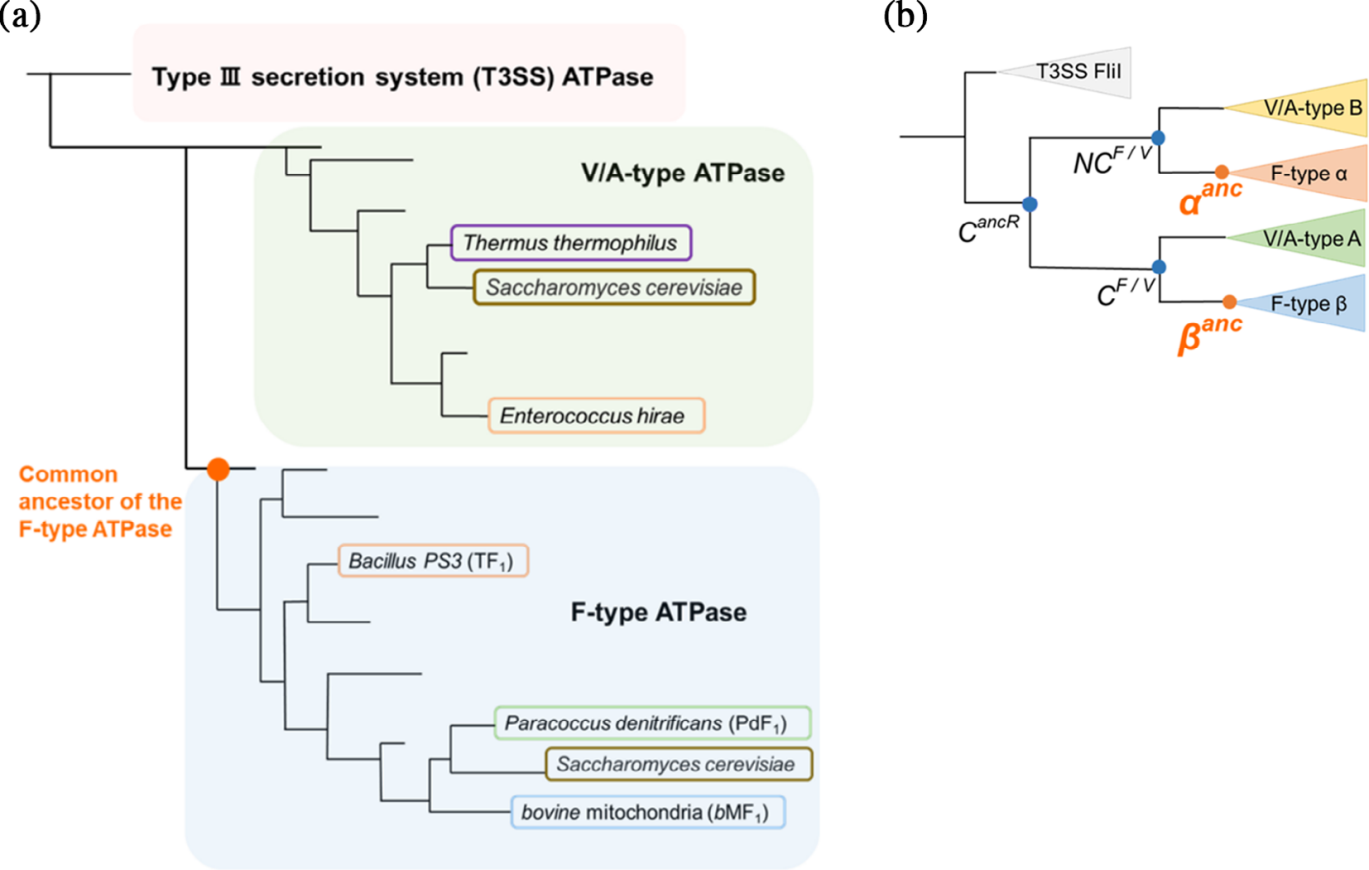
Schematic phylogenetic trees of rotary ATPases. (a) Phylogenetic tree of rotary ATPases. The diagram highlights key species whose rotation schemes have been elucidated through single‐molecule rotation assays. Species belonging to the same phylum are represented in the same color. (b) Conceptual phylogenetic tree of the subunits forming the hexameric ring of rotary ATPases. The node labeled *C* ^*anc*^ represents the common ancestor of the α, β, A, and B subunits. *α* ^*anc*^ and *β* ^*anc*^ indicate the ancestral forms of the F‐type ATPase α and β subunits, respectively, with similar annotations for other subunits. In this study, *α* ^*anc*^ and *β* ^*anc*^ are collectively considered as the ancestral form of the F‐type ATPase.

Evolutionarily, F‐type ATPase is closely related to V/A‐type ATPase (Gogarten et al., 1989). V/A‐type ATPases, which are found in some eubacteria and archaea, are structurally similar to eukaryotic V‐type ATPases, suggesting a conserved architectural framework across domains. In this study, V‐type ATPases and A‐type ATPases are collectively classified as a single family, referred to as V/A‐type ATPases, in the phylogenetic analysis. The catalytic portion of V/A‐type ATPases functions as an ATP‐driven proton pump in the vacuoles of mammalian cells or as an ATP synthase in the plasma membranes of archaea (Figure 1a). V_1_‐ATPase is also an ATP‐driven rotary motor, rotating counterclockwise (when viewed from the membrane side) in the same direction as F_1_ (Ueno et al., 2018). V_1_ consists of a rotor complex and a hetero‐hexameric stator ring composed of A and B subunits, corresponding to the β and the α subunits of F_1_, respectively. Like F_1_, a recent study of V_1_‐ATPase from *Enterococcus hirae* (EhV_1_) resolved these 120° steps into 40° and 80° substeps (Iida et al., 2019), whereas such substeps have not been observed for *Thermus thermophilus* V_1_ (TtV_1_) (Imamura et al., 2005). Thus, although V_1_‐ATPase shares some basic characteristics with F_1_‐ATPase, its stepping behavior does not directly clarify how the ancestral F_1_‐ATPase might have operated.

One experimentally feasible way to explore the functionality of ancestral enzymes is ancestral sequence reconstruction (ASR) (Dube et al., 2022; Matsui, 2021; Spence et al., 2021). ASR uses multiple sequence alignment (MSA) of extant species to infer the most probable amino acid changes along a phylogenetic tree, thereby predicting ancestral protein sequences. A variety of ancestral proteins have been successfully expressed and characterized. The feasibility of sequence reconstruction by ASR methods depends on the degree of sequence conservation in the extant enzymes. Although the rotor subunits of rotary ATPases are less conserved, the non‐catalytic and catalytic subunits of F_1_ and V_1_ show high sequence similarity, respectively, and even resemble each other. Molecular phylogenetic analyses suggest that the ancestral motors of F_1_‐ATPase and V_1_‐ATPase diverged from a common ancestral rotary motor with a hetero‐hexameric stator ring comprising the ancestral non‐catalytic (N*C* ^*F* */* *V*^) and catalytic (*C* ^*F* */* *V*^) subunits (Figure 1b). It has also been proposed that this common ancestral rotary ATPase diverged from the homo‐hexameric ATPase from which the type III secretion system (T3SS) ATPase originated (Gogarten et al., 1989; Kibak et al., 1992) (Figure 1a,b). Divergence time estimates indicate that the ancestral of either or both F_1_‐ and V_1_‐ATPases—had already emerged as the catalytic portion of ATP synthase in LUCA (Mahendrarajah et al., 2023; Moody et al., 2024).

Thus, previous studies have established an evolutionary phylogeny of rotary ATPases that stems from the pre‐LUCA era (Mahendrarajah et al., 2023), laying a foundation for the ancestral sequence inference of these ancient rotary ATPases. However, ancestral sequence reconstruction of multi‐subunit complex enzymes requires extremely precise estimation of the amino acids forming the subunit–subunit interfaces, and consequently, there have been only a few successful examples (Schupfner et al., 2019). In particular, there have been no reports of ancestral sequence research on molecular machines like F_1_‐ATPase, which show large conformational transitions.

In the present study, we reconstructed the amino acid sequences of the catalytic and non‐catalytic subunits of the common ancestral F_1_‐ATPase and prepared these subunits for experimental investigation to understand how the ancestral proteins functioned. We also attempted to test the functionality of the reconstituted sequences. To address the technical challenge regarding the complex instability often observed in ancestral enzymes with multi‐subunit composition, we incorporated the functionally core parts from the ancestral sequences into extant thermostable F_1_, TF_1_; the non‐catalytic and catalytic subunits were designed as chimeras of the ancestral and extant proteins. Specifically, the functionally critical regions—namely, the nucleotide‐binding domain and the C‐terminal helical domain—were derived from the ancestral sequences, whereas the N‐terminal domains were taken from TF_1_ to serve as a structural scaffold. We then co‐expressed these chimeric subunits with the γ subunit from TF_1_, thereby forming a functional and stable F_1_ complex, of which core parts are derived from the ancestral sequence.

## 2 RESULTS

### 2.1 Ancestral sequence reconstruction

Previous studies have elucidated the phylogenetic branching of subunits forming hexameric rings in T3SS ATPase, V_1_‐ATPase, and F_1_‐ATPase (Gogarten et al., 1989; Kibak et al., 1992). In this study, we focused on the five families of subunits represented in the phylogenetic tree (Figure 1b): FliI subunit comprising of the homo‐hexameric ring of T3SS ATPase, the non‐catalytic subunit and the catalytic subunits of the hetero‐hexameric rings of V_1_‐ and F_1_‐ATPase (the B and the A subunits for V_1_‐ATPase, and the α and the β subunits for F_1_‐ATPase). Representative sequence data were downloaded from National Center for Biotechnology Information (NCBI) database and used as query sequences. These query sequences were subjected to BLASTP searches (Altschul et al., 1997) to collect sequences with a similarity above a certain threshold, which were then compiled into datasets for each subunit. Multiple sequence alignment was performed for each subunit. Sequences with large deletions, insertions, or redundancies were removed. As a result, we curated sequence datasets with conserved regions aligned for each subunit.

The final numbers of sequences used for phylogenetic tree construction were 94 for the T3SS ATPase FliI subunit, 128 for the A subunit and 100 for the B subunit of V_1_‐ATPase, and 142 for the α subunit and 153 for the β subunit of F_1_‐ATPase. Using these datasets, phylogenetic trees were inferred with IQ‐TREE (Nguyen et al., 2015) (Figure S1, Supporting Information), a fast and widely used maximum‐likelihood‐based software that incorporates advanced model selection. The phylogenetic tree constructed was compared with those from previous research, focusing on the branching positions of phyla that were consistently present across the trees. The analysis revealed a high degree of concordance in the branching positions (Figure S2).

The phylogenetic tree generated by IQ‐TREE (Nguyen et al., 2015) served as a scaffold for ancestral sequence reconstruction using the codeml program in the PAML package (Yang, 2007), a tool for maximum‐likelihood‐based evolutionary analysis, and GASP (Edwards & Shields, 2004), a probabilistic method for ancestral sequence inference. Amino acid residues at gap positions in GASP‐reconstructed sequences were removed to obtain the final ancestral sequences. The reliability of the reconstructed ancestral sequences was evaluated by the probability values: approximately 0.9 for the ancestral non‐catalytic subunits and catalytic subunits of F_1_‐ATPase (*α* ^*anc*^ and *β* ^*anc*^) and those for V_1_‐ATPases (*B* ^*anc*^ and *A* ^*anc*^), (Figure S3), ensuring the high reliability of the reconstructed sequences. The common ancestral non‐catalytic subunit and catalytic subunit of F_1_‐ and V_1_‐ATPase (*NC* ^*F/V*^ and *C* ^*F* */V*^) showed the probability values around 0.65 (Figure S3). The common ancestor protein of *NC* ^*F/V*^ and *C* ^*F* */V*^, *C* ^*ancR*^, which is supposed to form homo‐hexameric ATPase also shows a similar probability value, 0.67 (Figure S3), suggesting the sequence reconstruction of the more upstream common ancestral proteins is less reliable. To ensure the validity of the ancestral sequence inference, we also reconstructed a phylogenetic tree using RAxML (Stamatakis, 2014), a software optimized for rapid and efficient phylogenic tree searches, on which the ancestral sequence estimations was conducted. The reconstructed ancestral sequence confirms high consistency with the sequences reconstructed from the IQ‐TREE‐based phylogenetic trees, particularly the sequence region encoding the structurally interior parts of the subunits (Figure S4). The sequence regions encoding subunit–subunit interfaces formed in the F_1_ complex also shows high consistency. The differences between sequences reconstructed on IQ‐TREE‐ or RAxML‐based phylogenic trees are mainly found in the structurally exterior parts and the N‐terminal domains that are thought not to be crucial for functionality. Based on these results, we concluded that the ancestral sequences inferred here represent the most plausible reconstructions achievable with contemporary computational methods. In followings, we focus the sequences of *α* ^*anc*^ and *β* ^*anc*^ derived from the IQ‐TREE‐based phylogenic tree, while the reconstructed sequences for *NC* ^*F/V*^, *C* ^*F* */V*^ and *C* ^*ancR*^ were also analyzed (Tables S1 and S5–S8).

**FIGURE 2:**
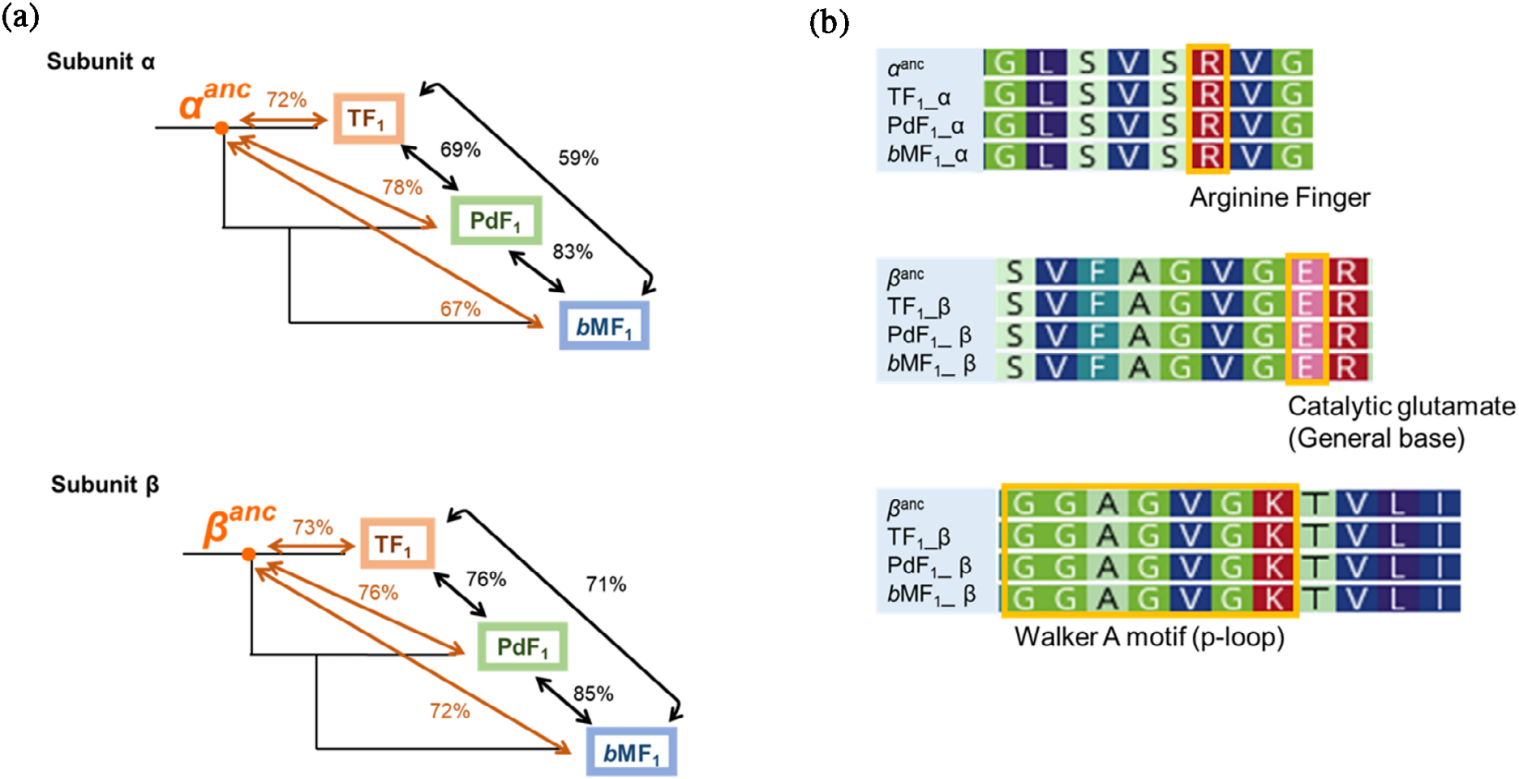
Sequence comparison between the common ancestor of F‐type ATPases and extant species. (a) Comparison of sequence identity between extant species and ancestral forms. The catalytic subunit β exhibits higher sequence identity across its full length compared to the non‐catalytic subunit α. Furthermore, the sequence identity between ancestral forms and extant species shows no substantial difference compared to the sequence identity among extant species. (b) Comparison of ATPase motifs. The conservation of key amino acid residues involved in ATP hydrolysis, including the arginine finger, catalytic glutamate, and p‐loop, was analyzed. *α* ^*anc*^ and *β* ^*anc*^ represent the ancestral sequences of the F‐type ATPase α and β subunits reconstructed in this study. The other three sequences correspond to the extant species TF_1_, PdF_1_, and *b*MF_1_. The ATPase motifs are well conserved across the compared sequences, suggesting that ancestral forms utilize the same key amino acid residues as extant species for ATP hydrolysis.

The sequences of the ancestral non‐catalytic and catalytic subunits, *α* ^*anc*^ and *β* ^*anc*^ were compared with ones of the extant F_1_‐ATPases, TF_1_, PdF_1_, and *b*MF_1_. The identities between the ancestral and the extant F_1_‐ATPases were approximately 70%, while sequence identities among extant F_1_‐ATPase ranged from 75% to 85% (Figure 2a). As predicted from the high sequence conservation, we found the perfect conservation of the catalytically crucial sequences among the ancestral sequences and the extant sequences: regions including arginine finger, catalytic glutamate, and Walker motif A (phosphate binding loop, p‐loop) (Figure 2b). Thus, the sequence comparison between the sequences of the ancestral subunits and the extant ones supports the plausibility of the reconstructed sequences of *α* ^*anc*^ and *β* ^*anc*^, suggesting the functionality of these subunits. Note that the catalytic residues are also fully conserved among *NC* ^*F/V*^, *C* ^*F* */V*^ and *C* ^*ancR*^ (Figure S7).

### 2.2 Biochemical analysis

**FIGURE 3:**
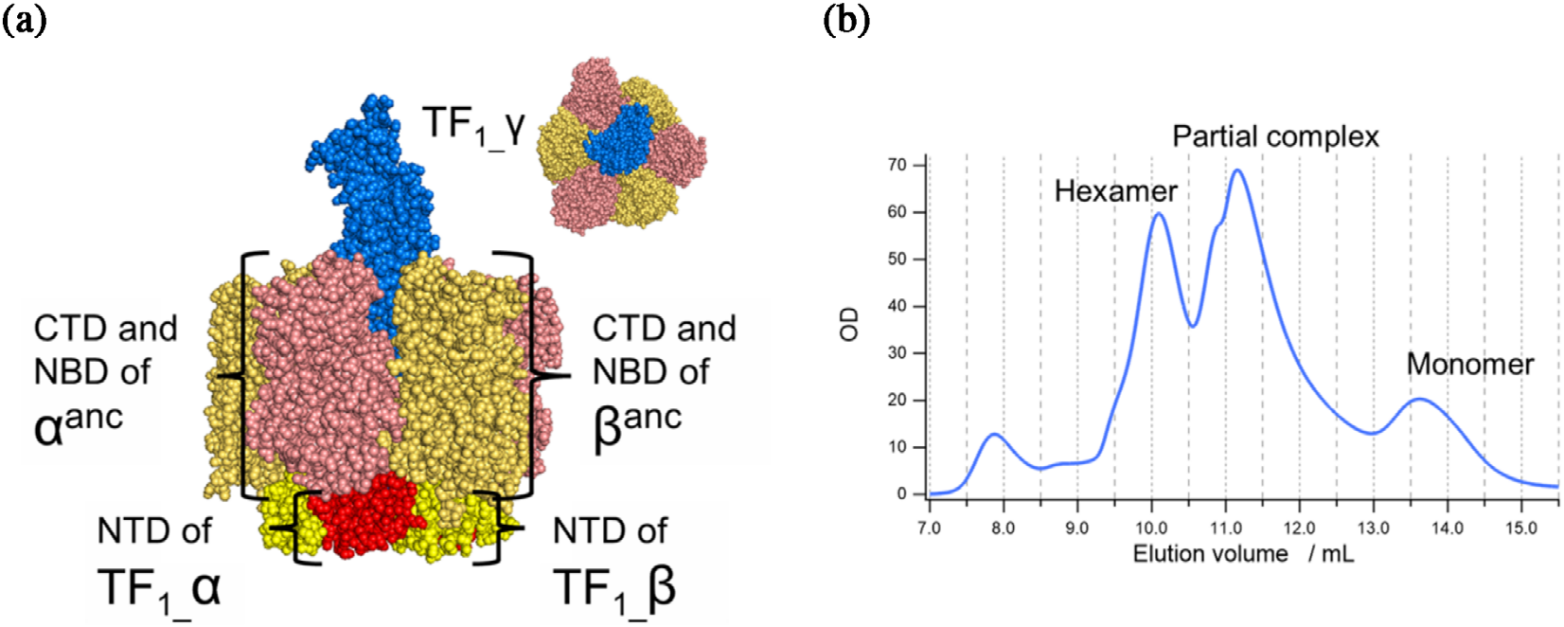
The design and purification of F_1_^anc_core^. (a) The design of F_1_^anc_core^. The hexameric ring consists of α and β subunits, where the N‐terminal β‐barrel domains (NTD), forming the structural foundation, are derived from the extant species TF_1_, while the remaining regions C‐terminal domain (CTD) and nucleotide‐binding domain (NBD) utilize the reconstructed ancestral sequences of the F‐type ATPase (*α* ^*anc*^, *β* ^*anc*^). The γ subunit forming the central stalk is based on the sequence of TF_1_. (b) Size‐exclusion HPLC chromatogram after Ni‐NTA purification of F_1_^anc_core^. The peak positions were estimated using a calibration curve generated with molecular weight markers. The peak corresponding to the hexameric complex aligns with the peak position observed during the purification of TF_1_, which has a comparable molecular weight. Peaks corresponding to partial complex and monomeric forms were also detected; however, the peak for the fully assembled hexameric complex is distinctly observed.

To test the functionality of the ancestral subunits, we attempted to express F_1_ composed of the ancestral subunits. ASR of enzymes with multis‐subunits is challenging, due to the difficulty of highly precise inference of residues forming subunit–subunit interfaces. To reinforce the structural integrity, TF_1_ was used as scaffold to accommodate the functionally crucial core domains of *α* ^*anc*^ and *β* ^*anc*^; nucleotide‐binding domain (NDB) and C‐terminal domain (CTD) of *α* ^*anc*^ and *β* ^*anc*^ were genetically fused with the N‐terminal domain (NTD) of TF_1_ that forms the stable hexameric scaffold (residues α1‐94 and β1‐78 in TF_1_). The chimeric *α* and *β* subunits were co‐expressed with the γ subunit of TF_1_ (Figure 3a). The resultant hybrid F_1_ with the ancestral core domains, referred hereafter to as F_1_^anc_core^ for simplicity, was successfully expressed as a stable complex as shown in the elution profile of size‐exclusion chromatography; it showed the distinctive peak of F_1_ complexes beside peaks presumably corresponding to partially formed complexes and monomers (Figure 3b).

The ATPase activity of F_1_^anc_core^ was assessed using an ATP regeneration system. F_1_^anc_core^ exhibited ATPase activity approximately one‐tenth that of TF_1_ (Table 1). Despite the low activity level, ATP hydrolysis was clearly evident. Notably, the ATPase activity after LDAO addition remained unchanged. LDAO is known to relieve ADP inhibition, a state in which ADP tightly remains bound on the catalytic site to prevent catalysis and rotation (Hirono‐Hara et al., 2001). These findings suggest that the low ATPase activity of F_1_^anc_core^ reflects intrinsically low activity.

**TABLE 1:** ATPase activity values.

Ancestral F_1_‐ATPase		TF_1_	
w/o LDAO	w/ LDAO	w/o LDAO	w/ LDAO
6.7 ± 0.1 s^−1^	7.5 ± 0.3 s^−1^	78 ± 3 s^−1^	140 ± 1 s^−1^

*Note*: The activity measured within the first 300 s of the experiment. Values are mean ± SD (*n* = 3).

### 2.3 Structural analysis

The structure of F_1_^anc_core^ was determined by Cryo‐EM, following our previous studies (Sobti et al., 2021). The purified sample was applied to EM grids at 22°C, vitrified in liquid ethane, and imaged using single‐particle analysis (SPA) at 300 kV. When the grids were prepared in the presence of AMP‐PNP—a nonhydrolyzable analog of ATP—only about 5% of the molecules were observed to form intact F_1_ complex, suggesting that the sample readily dissociates under those conditions (Table S2). To overcome this, we instead prepared EM grids without any added nucleotides. Under this condition, five distinct Cryo‐EM maps were obtained: two fully assembled F_1_ complexes with different γ‐subunit rotational angles, a hexameric ring lacking the central shaft, and we also partially assembled complexes (a tetramer with the central shaft and a tetramer without it; see Figures S9–S11). The resolutions of the cryo‐EM maps were determined to be 2.5 Å for the two fully assembled complexes, 2.8 Å for the hexameric ring without the shaft, 2.5 Å for the tetramer with the shaft, and 2.7 Å for the tetramer without the shaft, using the “gold standard” method.

**FIGURE 4:**
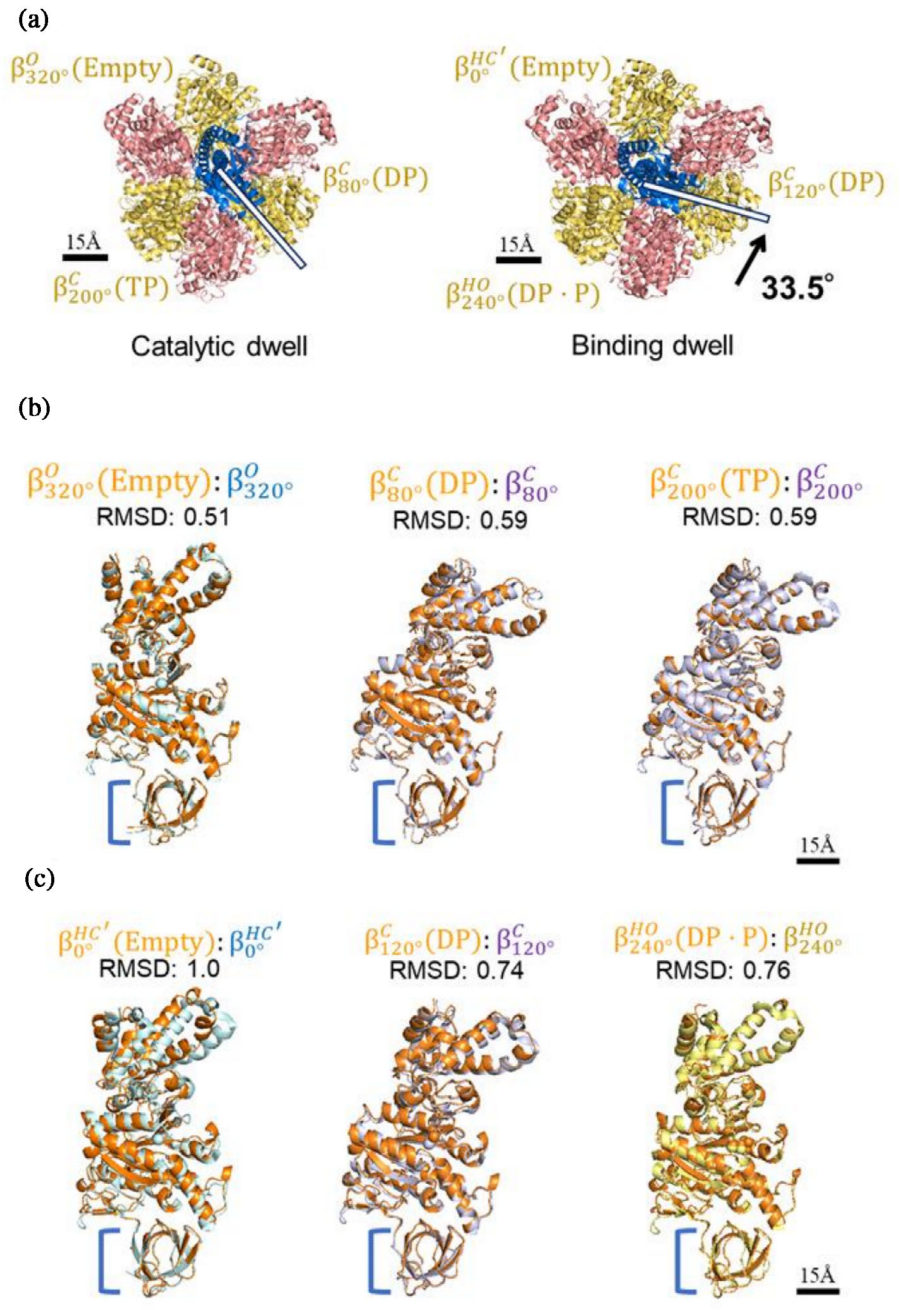
Structure of F_1_^anc_core^. (a) Structures of the two rotational dwells of F_1_^anc_core^. The structures from the top are shown, with subunits colored as in Figure 3a. Comparison of the Catalytic dwell and Binding dwell suggests that the γ subunit rotates counterclockwise between the two dwells (rotation highlighted with white bars and a black arrow). Each dwell exhibits three distinct conformations of the β‐subunits, illustrating the six sub‐states (termed $\beta_{0{}^{\circ}}^{{\mathrm{HC}}^{\prime }}\left(\text{Empty}\right)$, $\beta_{80{}^{\circ}}^{C}\left(\mathrm{DP}\right)$, $\beta_{120{}^{\circ}}^{C}\left(\mathrm{DP}\right)$, $\beta_{200{}^{\circ}}^{C}\left(\mathrm{TP}\right)$, $\beta_{240{}^{\circ}}^{\mathrm{HO}}\left(\mathrm{DP}\cdot P\right)$, $\beta_{320{}^{\circ}}^{O}\left(\text{Empty}\right)$) through which the enzyme progresses during its hydrolysis cycle. (b, c) Comparison of the β conformations of F_1_^anc_core^. The β subunit structure of F_1_^anc_core^ was superimposed onto the corresponding β subunit structures of TF_1_, with a focus on the N‐terminal 81 amino acid residues forming the β‐barrel structure. F_1_^anc_core^ is depicted in orange, TF_1_ in the open conformation in blue, TF_1_ in the closed conformation in purple, and TF_1_ in the half‐open conformation in yellow. RMSD values (Å) indicate the structural differences between the aligned models. (b) Superimposition of the three β subunits in the catalytic state. The TF_1_ structure is based on PDB entry 7L1R. (c) Superimposition of the three β subunits in the binding state. The TF_1_ structure is based on PDB entry 7L1Q.

A comparison of the two fully assembled complexes with previously published cryo‐EM structures of TF_1_ (PDB IDs: 7L1R for the catalytic dwell and 7L1Q for the binding dwell) showed a good overall match, with root mean square deviations (RMSDs) of 0.75 Å and 0.95 Å, respectively, allowing us to assign one complex as the catalytic dwell and the other as the binding dwell (Figure 4a). RMSD values (Å) indicate the structural differences between the aligned models. The rotational angle difference of the γ subunit between the binding dwell and catalytic dwell in F_1_^anc_core^ was approximately 34°, smaller than that of TF_1_ (Sobti et al., 2021) (~44°). Structural comparison of the β subunits between F_1_^anc_core^ and TF_1_ showed that they were highly similar (Figure 4b,c). At the ~80°, ~120°, and ~320° rotational states, the β subunits of F_1_^anc_core^ were bound to ADP, ADP, and no nucleotide, respectively, whereas in TF_1_ the equivalent sites carried ATP, ATP, and ADP (Figures S12–13). These differences in nucleotide occupancy likely arise from the absence of exogenous nucleotides during sample preparation. This suggests that the observed structures represent a state in which the enzyme re‐establishes equilibrium with nucleotides originally carried over in bound form, rather than an authentic catalytic intermediate. Consistent with this overall structural similarity, the three‐dimensional arrangement of conserved ATPase motif residues in the nucleotide‐binding site is also well preserved between the ancestral and extant *b*MF_1_ (Figure S14), in line with the sequence alignment shown in Figure 2b.

TF_1_ is known to operate via a six‐step rotational catalytic mechanism (Noji et al., 2017; Sobti et al., 2021). Given that the β subunit structures of F_1_^anc_core^ correspond one‐to‐one with those of TF_1_, we infer that F_1_^anc_core^ also operates via a six‐step rotational catalytic mechanism similar to TF_1_. Structural alignment of the hexameric ring components (chains A–F) between F_1_^anc_core^ lacking the γ subunit and the TF_1_ binding dwell state yielded an RMSD of 0.002 Å, indicating a high degree of structural conservation within the ring. Since the hexameric ring without the central γ subunit is considered unaffected by the central γ subunit derived from TF_1_, it is highly likely that the stator ring of F_1_^anc_core^ is prone to adopt the binding dwell structure without the γ subunit. Regarding the catalytic dwell, all structural analyses of extant species to date have consistently observed the catalytic dwell structure. Taking these points into account, it is highly likely that F_1_^anc_core^ had two distinct conformational states—binding dwell state and catalytic dwell state—as same as TF_1_. However, the angular orientation of the γ subunit in the binding dwell and catalytic dwell states is influenced by the species from which the γ subunit is derived (Watanabe et al., 2023). Therefore, the magnitude of the rotational angle difference between the two states in the F_1_ complex fully composed of the ancestral subunits may vary.

### 2.4 Rotation analysis

**FIGURE 5:**
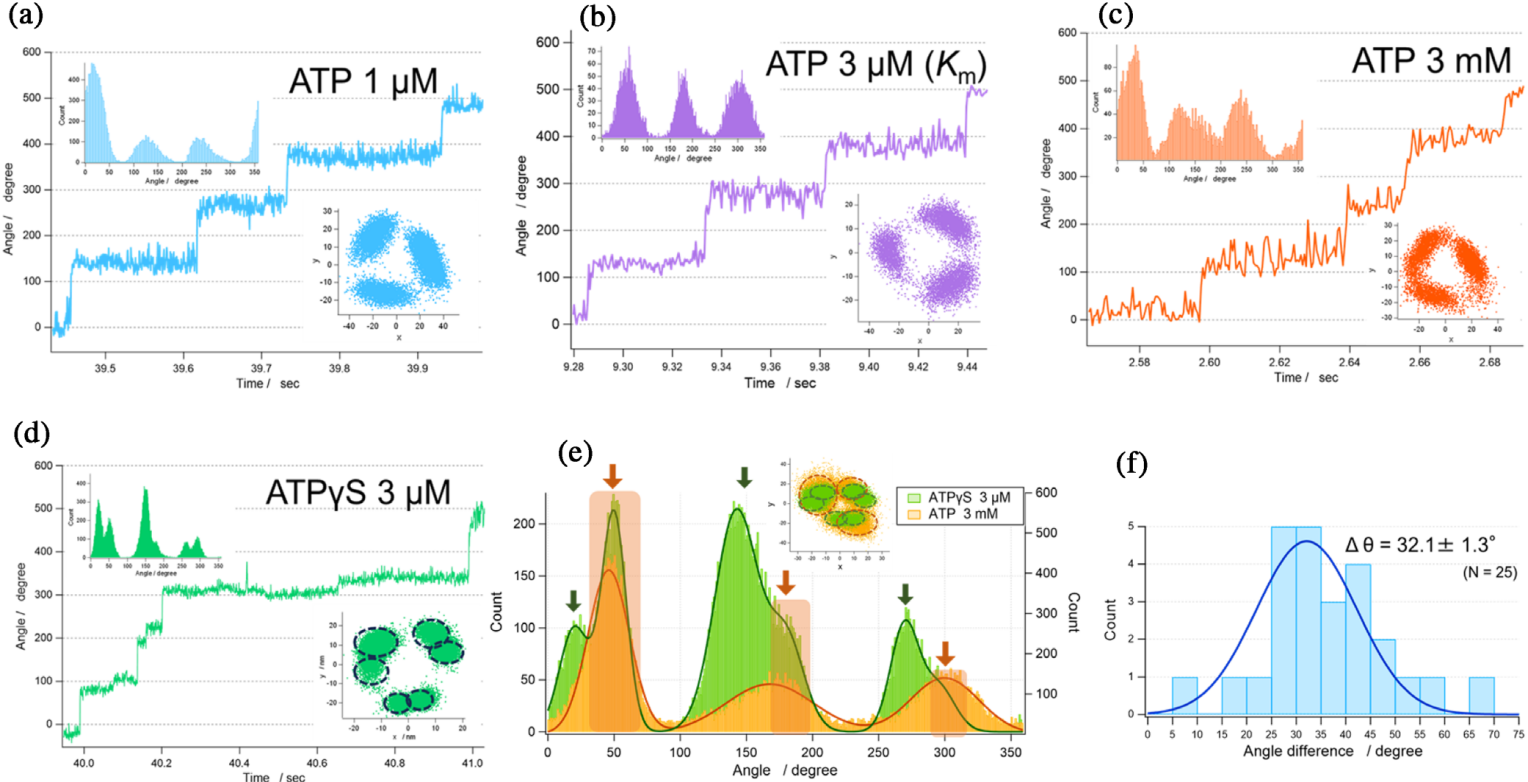
Single‐molecule rotation assay of F_1_^anc_core^. (a–d) Time courses of rotation. The angular position histograms (left), and the x–y plots of the centroid of a rotating gold colloid (right) are shown in inset. (a) 1 μM ATP. (b) 3 μM ATP (*K*_m_). (c) 3 mM ATP. (d) 3 μM ATPγS. (e) The angular position histograms of 3 mM ATP (orange) and 3 μM ATPγS (green). The x–y plots are shown in inset. The arrows show the angular position of catalytic dwell (green) and binding dwell (orange). (f) Histogram of angular differences between catalytic dwell and binding dwell positions. Values are mean ± SD (*n* = 25, 10 molecules).

Single‐molecule rotation assays of F_1_^anc_core^ were performed using 40 nm gold colloid particles as the rotational probe, observed with a laser dark‐field microscope at 2000 fps (frames per second) (Ueno et al., 2010). F_1_^anc_core^ rotated in a counterclockwise direction, exhibiting three distinct rotational pauses at mM level of [ATP] (Figure 5c). Single‐molecule rotation assays under various ATP concentrations allowed determination of the *V*_max_ and *K*_m_ values through Michaelis–Menten kinetics analysis, which were calculated to be 15.4 rps and 2.6 μM, respectively (Figure S15). These *V*_max_ and *K*_m_ values were approximately one‐tenth of those observed for TF_1_. The rate constant of ATP binding, *k*_on_, estimated as 3 × *V*_max_/*K*_m_, was determined to be 1.6 × 10^7^ M^−1^ s^−1^, which is very close to that of TF_1_ (1.8 × 10^7^ M^−1^ s^−1^). Thus, the major kinetic difference is the slow maximum rotation speed.

The maximum rotational velocity 15.4 rps corresponds to 46/s as *k*_cat_ of hydrolysis, that is significantly faster than the rate of hydrolysis estimated from biochemical ATPase activity assays performed with ATP regeneration system (Table 1). Such a discrepancy between the single‐molecule rotation assay and the biochemical assay has often been reported (Kobayashi et al., 2020; Yasuda et al., 2001; Zarco‐Zavala et al., 2020), and it is usually attributed to ADP‐inhibition. In the presence case, in addition to ADP‐inhibition, the apparently lower hydrolysis activity estimated from biochemical analysis can be attributed to the heterogeneity of the sample. Cryo‐EM analysis revealed that a significant fraction of the sample consisted of partial complexes lacking the γ subunit and/or αβ pair.

Another distinctive feature of F_1_^anc_core^ rotation is that it proceeds in three steps per turn at all of [ATP] examined (Figure 5a–c), whereas TF_1_ is known to make 80° and 40° substeps during each 120° rotation, particularly around *K*_m_ region making six steps per turn. The buffer exchange experiment where [ATP] was switched between high and low concentrations confirmed that the dwell positions observed at low and high [ATP]s were coincident with each other (Figure S16). This observation can be interpreted to mean that F_1_^anc_core^ performs both ATP binding and hydrolysis within the same dwell. However, such an interpretation contradicts the cryo‐EM analysis, which shows that F_1_^anc_core^ adopts two distinct conformational states—a binding dwell and a catalytic dwell—where the γ subunit's orientation differs by ~33.5° (Figure 4a). Another possibility is that F_1_^anc_core^ pauses for a prolonged period at the binding dwell angles while waiting on a reaction step other than ATP binding, and that the long pause dominates the overall reaction time, making the shorter catalytic dwells effectively negligible.

To test this hypothesis, we conducted the rotation assay in the presence of ATPγS, a slowly hydrolyzed ATP analog often used to identify the angular position of the catalytic dwell in the rotation assay of F_1_ (Kobayashi et al., 2020; Shimabukuro et al., 2003; Zarco‐Zavala et al., 2020). As expected, F_1_^anc_core^ rotated at significantly lower speed in ATPγS than in ATP; the maximum rate was 1.46 rps, which is around one‐tenth of ATP‐driven rotation (Figure S17). When the rotation was observed in the presence of 3 μM ATPγS (Figure 5d)—near the *K*_m_ range for ATPγS‐driven rotation (0.68 μM)—six rotational dwells were identified, separated by 32.1°, consistent with the observations from cryo‐EM analysis. To correlate these ATPγS‐based dwell positions with those observed under ATP, we performed solution exchange experiments, alternately supplying ATP and ATPγS (Figure 5e). By comparing the dwell positions in both conditions, we found that three of the six dwell points observed with ATPγS matched those under ATP. Based on this result, we assigned the three shared dwell points, seen with both ATP and ATPγS, to the binding dwell, whereas the dwell points observed exclusively under ATPγS were identified as the catalytic dwells. The analysis of the angular differences of the binding dwell from the catalytic dwell revealed a histogram centered at 32.1° (Figure 5f). This angular difference aligns closely with the rotational angle difference of ~34° between the binding and catalytic dwells of the γ subunit observed in Cryo‐EM structural analyses, supporting the above assignment. Thus, it was confirmed that F_1_^anc_core^ has two distinct states pausing at binding dwell angles or catalytic dwell angles as expected from Cryo‐EM analysis, while the motor pauses predominantly at binding dwell angles waiting for a long reaction step to occur under ATP conditions. TF_1_ is reported to make pauses at binding dwell angles when observed at low temperatures, due to temperature sensitive reaction (TS reaction). To test the possibility of TS reaction, we measured ATP hydrolysis activity at low temperatures. However, *Q*_10_ factor estimated from ATPase assay for F_1_^anc_core^ was 1.52, too low to attribute the rate‐limiting step as TS reaction (Table S3). Thus, the long pause found at binding dwell angles should be a new class of reaction or conformational state.

## 3 DISCUSSION

In this study, we focused on the phylogenetic tree of rotary ATPases and inferred ancestral sequences of the hexameric ring–forming subunits at multiple nodes: the ancestral F_1_‐ATPase, the common ancestral rotary ATPase of F_1_‐ and V_1_‐ATPases, and an even earlier rotary ATPase with a homo‐hexameric ring. As a result, we obtained subunit sequences for these ancestral rings—*α* ^*anc*^, *β* ^*anc*^, *C* ^*F* */V*^, *NC* ^*F/V*^, and *C* ^*ancR*^. Comparative analysis revealed that these ancestral rotary ATPases already possessed the key motifs required for ATP hydrolysis—namely, the arginine‐finger, catalytic glutamate, and Walker motif A—that are almost universally found in extant F_1_‐ATPases (Figure 2b, Figure S7). Although we did not perform a quantitative structural analysis, the predicted structures of these ancestral subunits closely resemble those of modern subunits (Figure S8).

Considering that LUCA employed either the ancestral rotary ATPase corresponding to F_1_ or V_1_‐ATPase (or both) (Mahendrarajah et al., 2023; Moody et al., 2024), the common ancestor of F_1_ and V_1_ must have already arisen as a rotary ATPase with a hexameric ring in the pre‐LUCA era. It is also plausible that the ancestral ATPase diverging from the type III secretion system (T3SS) ATPase possessed a hexameric ring structure, given that extant T3SS ATPases likewise form hexamers (Kibak et al., 1992; Majewski et al., 2019).

Before expressing F_1_^anc_core^, which carries *α* ^*anc*^ and *β* ^*anc*^ sequences in its core domains, we first attempted to produce several ancestral ATPases—namely *C* ^*F* */V*^, *NC* ^*F/V*^, and *C* ^*ancR*^. However, none of these proteins formed stable complexes, indicating that it remains challenging to obtain functional complexes solely from ancestral sequences with our current knowledge of rotary ATPase and the present ASR methodology. To address this issue, we constructed a chimeric ATPase using an extant F_1_ (TF_1_) as a structural scaffold. Specifically, we combined the TF_1_ N‐terminal domain (NTD), which forms the base of the hexameric ring, with the ancestral sequences for the functionally essential domains—the nucleotide‐binding domain (NBD) and the C‐terminal domain (CTD) of *α* ^*anc*^ and *β* ^*anc*^. When expressed with the γ subunit of TF_1_, this design yielded a stable F_1_ complex, termed F_1_^anc_core^.

F_1_^anc_core^ was expressed as a stable complex exhibiting ATPase activity although the activity is significantly lower than TF_1_. Cryo‐EM structural analysis revealed the significant fraction of molecules retained the α_3_β_3_γ complex although partial complexes such as α_3_β_3_, α_2_β_2_γ, or α_2_β_2_ subcomplexes were also found. The α_3_β_3_γ complex structure of F_1_^anc_core^ exhibited the two conformations. Comparison with TF_1_ structures identified the two conformational states as the binding dwell state and catalytic dwell state, indicating that F_1_^anc_core^ shares the same rotational catalytic mechanism as same as TF_1_. Interestingly, the conformational states of the α_3_β_3_ subcomplex corresponds to the binding dwell state as found in the α_3_β_3_ subcomplex of TF_1_, reinforcing the abovementioned idea that F_1_^anc_core^ operates the similar rotational catalytic mechanism to TF_1_, alternating the conformational state between binding dwell state and catalytic dwell.

The result of the rotation assay of F_1_^anc_core^ was apparently against this expectation. F_1_^anc_cor^ showed a three‐step rotation at all tested [ATP], indicating that it consistently pauses at binding dwell angles. Presuming that this apparent three‐step pattern arises from an extended binding dwell overshadowing a brief catalytic dwell, we conducted rotation assays with ATPγS. Under these conditions, we observed well‐defined stepping, with six pauses per turn. A subsequent buffer‐exchange experiment—switching between ATP and ATPγS—revealed that F_1_^anc_core^ pauses at both binding and catalytic dwell angles, which differ by 32.1°. This value closely matches the ~34° difference determined by Cryo‐EM analysis.

Thus, F_1_^anc_core^ essentially follows a reaction scheme similar to that of TF_1_, featuring two stable conformational states (binding dwell and catalytic dwell), even though the binding dwell of F_1_^anc_core^ is significantly longer than its catalytic dwell (Figure S18). The molecular mechanism behind this prolonged binding dwell remains unclear. We hypothesized it might stem from a temperature‐sensitive process, akin to what has been observed in TF_1_ rotation assays at low temperatures. However, the *Q*_10_ factor for the ATPase activity of F_1_^anc_core^ is only 1.52—too low for a typical temperature‐sensitive reaction. A candidate reaction responsible for the long‐lived dwell is the ADP release step that was suggested at or near the binding dwell angles (Adachi et al., 2007; Noji & Ueno, 2022). Further investigation remains to address this point.

**FIGURE 6:**
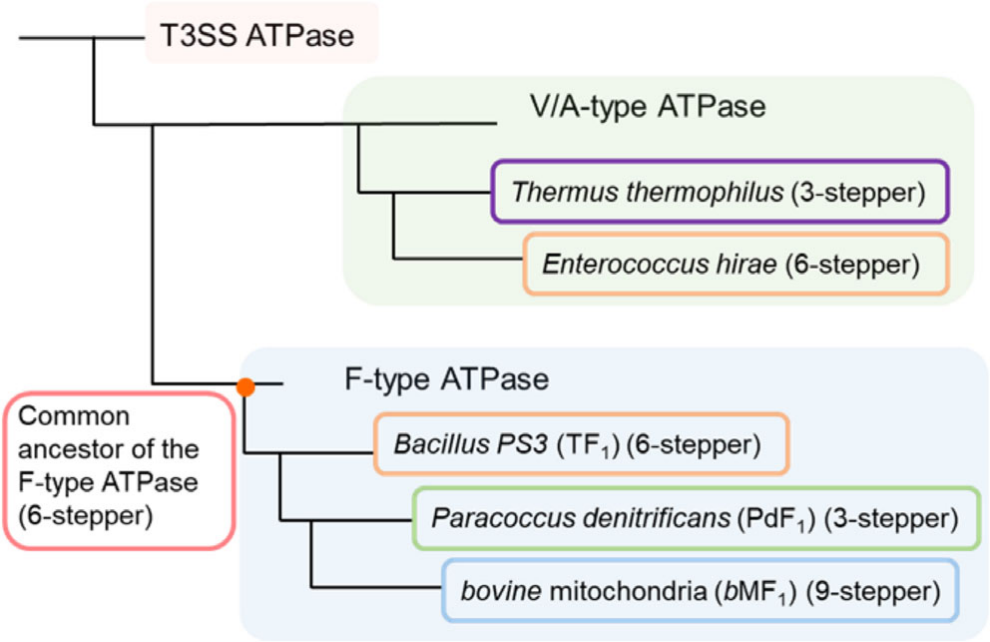
Differences in rotational catalytic mechanisms among species and their branching positions. The branching positions and unique features of the rotational catalytic mechanisms for species with well‐characterized mechanisms are presented.

Extant F_1_‐ATPases exhibit diverse rotary mechanisms, classified as three‐steppers, six‐steppers, or nine‐steppers. Our findings suggest that the ancestral F_1_‐ATPase was fundamentally a six‐stepper. One might argue that if the binding dwell is relatively longer than the catalytic dwell, the motor could effectively appear to be a three‐stepper. Yet, PdF_1_, the only known three‐stepper motor, still displays three‐step rotation even when measured with ATPγS, differing it from F_1_^anc_core^ described here. Consequently, it seems more appropriate to conclude that the ancestral F_1_ was intrinsically a six‐stepper, and it diversified into more three‐steppers, six‐steppers, and nine‐steppers (Figure 6).

We previously identified an intriguing correlation between the number of rotational steps of F_1_ and the number of the *c*‐subunits in the *c*‐ring (Noji et al., 2020), although more data is needed to confirm its generality. From this correlation, one empirical rule emerges: a 6‐stepper F_1_ pair with a 10‐stepper F_o_ (i.e., 10 *c*‐subunits). This rule suggests that ancestral F_1_ likely paired with a F_o_ containing a *c*_10_‐ring. If the free energy of ATP hydrolysis was comparable to that of extant cells, then LUCA and related lineages must have already possessed electrically tight plasma membranes capable of maintaining a sufficiently high *pmf*. This follows from the fact that the number of *c*‐subunits is one of the key factors determining the H^+^/ATP ratio and the equilibrium *pmf* between ATP hydrolysis and synthesis.

From an engineering perspective, this study highlights a new strategy: using TF_1_ as a scaffold for implementing various ancestral or designed catalytic domains, including the common subunits of F_1_‐ and V_1_‐ATPase, *C* ^*F* */* *V*^and *NC* ^F/V^. By extending this idea to the entire F_o_F_1_ complex, one could consider incorporating more ancestral‐sequence subunits into a stably structured F_o_F_1_—such as T F_o_F_1_ found in extant species—to more accurately estimate the function of ancestral F_o_F_1_. Currently, ancestral sequence reconstruction methods are largely restricted to highly conserved subunits, so beyond the α and β subunits of F_1_, they can only be applied to certain regions of the *c*‐subunit or the *a*‐subunit. Even so, studying chimeric or hybrid F_o_F_1_ complexes that include such ancestral functional units may yield insights into functions and dynamics that cannot be inferred from sequence analysis alone.

In future work, it will be important to extend our analysis in two directions. First, investigating the power‐stroke dynamics of ancestral F_1_ in comparison with thermophilic and mesophilic F_1_‐ATPases through torque measurements, although interpretations of the torque–angle relationship vary depending on measurement methodology and research groups (Kinosita et al., 2000; Saita et al., 2015; Sielaff et al., 2016). Second, testing the predicted inverse relationship between the number of rotational steps and the size of the c‐ring in photosynthetic F‐ATP synthases with c15‐rings would be an exciting avenue. Verifying whether such motors indeed exhibit fewer discrete steps per turn would deepen our understanding of the general principles governing rotary ATPases.

## 4 METHODS

### 4.1 Phylogenetic analysis and ancestral sequence inference

Protein sequences were retrieved from the NCBI (National Center for Biotechnology Information) database. In this study, the KF database ver.2021 (Furukawa et al., 2022) was further expanded for ATPase research, resulting in the KFS Database. The database comprised all protein sequences of 136 archaeal species, 594 bacterial species, and 77 eukaryotic species and was employed for BLAST searches.

Amino acid sequences for the α and β subunits of the F_1_‐ATPase from *bovine* mitochondria (ACCESSION: P19483, P00829), the A and B subunits of the V_1_‐ATPase from *Thermus thermophilus* (Q56403, Q56404), and the T3SS FliI protein from *E. coli* (NP_416451) were retrieved from NCBI as query sequences. The FliI family of the T3SS was incorporated into the phylogenetic tree as an outgroup to determine the root position of the F_1_‐ATPase and V_1_‐ATPase. These sequences were subjected to BLASTP searches (Altschul et al., 1997) against the KFS Database to collect homologous sequences. Redundant sequences and those with significantly different lengths were removed. The remaining 617 sequences were realigned using MAFFT (Katoh & Standley, 2013) with secondary structure considerations, followed by manual refinement to produce a multiple sequence alignment, which was subsequently used for phylogenetic analysis.

Alignment trimming was performed using TrimAl (Capella‐Gutiérrez et al., 2009) (version 1.4) in automated trimming mode (−automated1). Phylogenetic trees were constructed using IQ‐TREE (Nguyen et al., 2015) (version 2.1.3). The ModelFinder Plus (Kalyaanamoorthy et al., 2017) (MFP) module identified the LG + R10 model as the optimal amino acid substitution model. Ultra‐fast bootstrap analysis (Hoang et al., 2018) with 1000 replicates was conducted to evaluate branch confidence.

For additional reliability assessments, phylogenetic analysis was also conducted using RAxML (Stamatakis, 2014) (version 8.2.12). The LG + G + I model was selected, and bootstrap analysis with 1000 replicates was performed.

Phylogenetic analyses and ancestral sequence reconstruction were performed using the codeml program in the PAML package (version 4.9j, February 2020) (Yang, 2007) with the LG substitution model for amino acid sequences. Default parameters were used unless specified otherwise. The gap positions of ancestral sequences were inferred by GASP (Edwards & Shields, 2004) (version 2.0.0).

The ancestral sequences generated by PAML were compared with those by GASP. Regions identified as gaps in the ancestral sequence of GASP were excised from the PAML ancestral sequences to produce the final ancestral sequence set.

### 4.2 Preparation for F_1_^anc_core^

Genuine TF_1_ was prepared as described (Okuno et al., 2008). To visualize rotation, two cysteine residues were introduced as previously reported (Watanabe et al., 2023). The plasmid encoding F_1_^anc_core^ was constructed using the TF_1_ plasmid as a vector and PCR products encoding the C‐terminal and nucleotide‐binding domains of *α* ^anc^ and *β* ^anc^ (both with N‐terminal His‐tags) as insert DNAs. After ligation, the recombinant plasmid was introduced into the F_o_F_1_‐deficient *E. coli* strain JM103∆unc. F_1_^anc_core^ was then expressed in *E. coli*, purified, and biotinylated as described in ref (Rondelez et al., 2005). The protein concentration was determined by UV absorbance using a molar extinction coefficient of 182,500 M^−1^ cm^−1^, calculated from its amino acid sequence using the ProtParam tool (ExPASy).

### 4.3 Calibration curve for molecular weight determination by size‐exclusion HPLC

Molecular weight determination was performed using size‐exclusion high‐performance liquid chromatography (SEC‐HPLC). A calibration curve was generated using the MW‐Marker (HPLC) for Molecular Weight Determination (Oriental Yeast Co., Ltd.). Chromatographic analysis was conducted on an HPLC system equipped with a size‐exclusion column maintained at 25°C. The mobile phase consisted of 50 mM HEPES‐KOH (pH 7.5) containing 100 mM NaCl, delivered at a flow rate of 0.5 mL/min. UV absorbance was monitored at 280 nm.

Retention times of the marker proteins were recorded, and a calibration curve was constructed by plotting the logarithm of molecular weight against retention time. A linear regression analysis was performed to establish the calibration equation, which was subsequently used to estimate the molecular weight of F_1_^anc_core^. Calibration was validated through triplicate measurements, ensuring reproducibility within an acceptable standard deviation.

### 4.4 ATPase activity assay

ATPase activity was measured at 25°C in a buffer containing 50 mM Hepes‐KOH (pH 7.0), 50 mM KCl, 3 mM MgCl₂, and an ATP‐regenerating system (0.2 mM NADH, 2.5 mM phosphoenolpyruvate, 200 μg/mL pyruvate kinase, and 50 μg/mL lactate dehydrogenase). Activity was calculated from the slope of NADH absorbance during the first 300 s of measurement. LDAO‐stimulated activity was assessed by adding 0.3% LDAO to the reaction and calculating the slope of NADH absorbance thereafter.

### 4.5 Cryo‐EM grid preparation

A volume of 3.5 μL of purified F_1_^anc_core^ was applied to a glow‐discharged holey gold grid (UltrAufoil R 0.6/1.0, 200 mesh). The grids were blotted for 4 s at 22°C, blot force 0, and 100% humidity, then plunge‐frozen in liquid ethane using a FEI Vitrobot Mark IV.

### 4.6 Data collection

The grids were initially screened for ice thickness and particle density using a Thermo Fisher Scientific Talos Arctica transmission electron microscope (TEM) operating at 200 kV. Subsequently, the grids were transferred to a Thermo Fisher Scientific Titan Krios TEM operating at 300 kV, equipped with a Gatan BioQuantum energy filter (20 eV slit width) and a K3 camera. To mitigate orientation bias observed in an initial test sample, movie micrographs were recorded at tilt angles ranging from 20° to 40°, as these tilt angles had provided improved data on similar studies (Sobti et al., 2021) suggested by cryoEF (Naydenova & Russo, 2017), which indicated an optimal tilt angle of ~37°. Automatic data collection was performed using EPU (E Pluribus Unum, Thermo Fisher Scientific) at a nominal magnification of ×60,000 (displayed magnification of ×165,000 due to the energy filter), resulting in a pixel size of 0.84 Å. The total electron dose was set to 62 electrons per Å^2^, distributed over 80 frames with a total exposure time of 6.2 s. A total of 4373 movie micrographs were collected with a defocus range from −0.5 to −1.5 μm.

### 4.7 Data processing

All image processing and refinement were performed using CryoSPARC v4.4.1 (Punjani et al., 2017) using the default settings unless stated otherwise. Initially, micrographs were motion‐corrected, and defocus values were estimated using the patch‐based workflow with default parameters (5 Å max alignment, 500 B‐factor alignment, 1000–40,000 Å defocus search). Four hundred and two micrographs with ice thickness worse than 1.07 nm and CTFs worse than 4.0 Å were removed. Particles were automatically picked using Blob Picker (particle diameter 100–150 Å) on a sub‐set of the dataset (200 micrographs) to generate templates for picking. A total of 3,534,258 particles were picked using Template Picker on the entire dataset of 3971 micrographs. The picked particles were extracted with a box size of 256 pixels subjected to five rounds of two‐dimensional (2D) classification to exclude “junk” particles, such as those from aggregates or minor contaminants at the end of each round. After 2D classification, there were 690,155 particles that appeared to represent hexamers and 664,417 particles that appeared to represent tetramers. Hence, the 2D classes were separated into hexamers and tetramers, and the particles from each were processed separately.

For the hexamer particles, an initial model was generated with an Ab initio reconstruction in CryoSPARC and then used as an input for homogenous refinement. These aligned particles (from the homogenous refinement) were then subjected to 3D classification with five classes, using the solvent mask from the homogenous refinement. The 3D classification yielded four distinct objects: one class with 191,889 particles that was a hexamer with stalk in the binding dwell state, two classes that were merged into one with a total of 206,455 particles that was a hexamer with stalk in the hydrolysis dwell state, one class with 150,384 particles that was a hexamer without stalk, and one class with 141,427 particles that did not refine to a meaningful map (potentially junk or a mixture of conformations). Each of the three protein‐like maps was independently refined using non‐uniform refinement, yielding the final high‐resolution maps. Each class was independently processed through homogeneous refinement and non‐uniform refinements, yielding the final high‐resolution maps.

For the tetramer particles, an initial map was generated with Ab initio reconstruction, and this Ab initio map was then used as input for homogenous refinement. These aligned particles were then subjected to 3D classification with five classes, using the solvent mask from the homogenous refinement. The 3D classification yielded two different objects, representing tetramers with and without central stalks. Three classes were combined into a class representing a tetramer with a stalk (407,761 particles), and two classes were combined into a class representing a tetramer without a stalk (256,656 particles), and these were independently refined with non‐uniform refinement to yield the final maps. The number of particles at each stage is detailed in the supplementary flow chart (Figure S9), and the statistics for all the final maps and models generated in the study are listed in Figure S10 and Table S4. As some regions contained lower‐resolution features, DeepEMhancer (Sanchez‐Garcia et al., 2021) was applied to sharpen the maps, enhancing their interpretability in figures displaying the entire complex.

Non‐uniform refinement was repeated for all the maps with CryoSPARC v4.6.2, and maps were uploaded to the 3DFSC Processing Server (Tan et al., 2017) to obtain 3D FSC information for review.

### 4.8 Model building

Models were constructed for intact F_1_ complexes and refined using Coot (Emsley et al., 2010) and PHENIX (Afonine et al., 2018), with PDB structures 7L1Q (TF_1_ binding dwell cryo‐EM structure) and 7L1R (TF_1_ catalytic dwell cryo‐EM structure) serving as templates. Table S4 shows details of the refinement and validation statistics. Figures were made using PyMOL.

### 4.9 Estimation of γ‐subunit rotational angle

The rotational displacement of the γ subunit between the binding‐dwell and catalytic‐dwell conformations was measured in PyMOL (v4.6) using the “Angle Between Helices” plugin. The central γ‐helix axis was defined by a vector connecting the Cα atoms of residues 6 and 287 in chain G. The angle between the axes of the binding‐dwell and catalytic‐dwell structures yielded the reported ~34° rotational difference.

### 4.10 Single‐molecule rotation assay

Flow chambers were constructed using double‐sided tape as spacers and two cover glasses (18 × 18 mm^2^ and 24 × 32 mm^2^; Matsunami Glass). The bottom glass surface was coated with Ni‐NTA.

The basic assay buffer contained 50 mM Hepes‐KOH (pH 7.5), 100 mM KCl, and 5 mM MgCl₂. When ATP was used as the substrate, an ATP‐regenerating system (2 mM phosphoenolpyruvate and 100 μL/mL pyruvate kinase) was added.

The flow chamber was first incubated with basic buffer containing 5 mg/mL BSA (BSA buffer) for 5 min. F_1_ molecules (200–500 pM) in BSA buffer were then introduced and incubated for 10 min, followed by washing with BSA buffer to remove unbound molecules. Next, 40 nm gold nanoparticles (prepared as described in ref (Iida et al., 2019).) were introduced, incubated for 10 min, and unbound particles were removed by washing with substrate‐containing basic buffer.

Rotational assays were performed as described in ref (Iida et al., 2019). at room temperature. Recorded videos were analyzed using custom software.

## AUTHOR CONTRIBUTIONS

**Aya K. Suzuki:** Conceptualization; investigation; methodology; data curation; formal analysis; validation; visualization; writing – original draft; writing – review and editing; funding acquisition. **Ryutaro Furukawa:** Software; resources; data curation. **Meghna Sobti:** Formal analysis; investigation; resources; visualization; writing – review and editing; methodology; data curation. **Simon H. J. Brown:** Resources; formal analysis; data curation; investigation. **Alastair G. Stewart:** Methodology; formal analysis; visualization; resources; funding acquisition; writing – review and editing; data curation; investigation. **Satoshi Akanuma:** Writing – review and editing; resources; software; methodology. **Hiroshi Ueno:** Writing – review and editing; methodology; conceptualization; funding acquisition. **Hiroyuki Noji:** Writing – review and editing; funding acquisition; supervision; methodology; conceptualization; project administration.

## Supporting information


**Data S1:** Ancestral sequences and posterior probability.

**Data S2:** Cryosparc_Hexamer_nostalk_map.

**Data S3:** Hexamer_nostalk_pdb.

**Data S4:** Hexamer_withstalk_BindingDwell_map_sharp.

**Data S5:** Hexamer_withstalk_BindingDwell_map_sharp_deepEMhancer.

**Data S6:** Hexamer_withstalk_BindingDwell_pdb.

**Data S7:** Hexamer_withstalk_CatalyticDwell_map_sharp.

**Data S8:** Hexamer_withstalk_CatalyticDwell_map_sharp_deepEMhancer.

**Data S9:** Hexamer_withstalk_CatalyticDwell_pdb.

**Data S10:** Sequence_F‐type_alpha.

**Data S11:** Sequence_F‐type_beta.

**Data S12:** Sequence_T3SS_FliI.

**Data S13:** Sequence_V‐type_A.

**Data S14:** Sequence_V‐type_B.

**Data S15:** Tetramer_nostalk_BindingDwell_map_sharp.

**Data S16:** Tetramer_nostalk_BindingDwell_map_sharp_deepEMhancer.

**Data S17:** Tetramer_nostalk_BindingDwell_pdb.

**Data S18:** Supplementary information.

## Data Availability

The cryo‐EM density maps have been deposited in the Electron Microscopy Data Bank under accession codes EMD‐49841, EMD‐49839, EMD‐49840, EMD‐49843, and EMD‐49842. The corresponding atomic models have been deposited in the Protein Data Bank under accession codes 9NVL and 9NVM. Additional data are available within the article and its supplementary material.
