# Supplementary material for: Functional and structural characterization of F1 ‐ATPase with common ancestral core domains in stator ring
Source: Protein Sci. 2025 Oct 23;34(11):e70345. doi: 10.1002/pro.70345 (PMC12550136; doi:10.1002/pro.70345)
Supplement: Supplementary file 1 — Data S1: Ancestral sequences and posterior probability. [file PRO-34-e70345-s013.pdf]

Sequence	T	G	R	V	T	Q	I	V	G	P	V	V	E	V	E	G	P	S	A	A	S	V	G	E	L	L	Q	I	E	G	N	G	E	I	L	A	E	V	V	G	F
Posterior probability	0.381	0.998	0.689	0.573	0.619	0.355	0.637	0.298	0.999	0.96	0.69	0.767	0.709	0.725	0.218	0.993	0.424	0.231	0.349	0.689	0.512	0.554	0.758	0.842	0.43	0.427	0.32	0.592	0.472	0.261	0.609	0.937	0.698	0.417	0.739	0.776	0.314	0.642	0.527	0.314	0.638
Sequence	T	G	R	V	T	K	I	K	G	P	V	V	E	V	Q	G	G	Q	G	A	E	I	G	E	V	V	R	I	H	G	D	G	K	V	L	A	E	V	V	G	F
Posterior probability	0.728	0.998	0.568	0.526	0.556	0.377	0.524	0.476	0.999	0.968	0.346	0.603	0.619	0.608	0.349	0.999	0.318	0.556	0.316	0.715	0.34	0.426	0.692	0.586	0.409	0.609	0.352	0.545	0.366	0.307	0.674	0.982	0.649	0.265	0.652	0.746	0.234	0.611	0.401	0.366	0.563
Sequence	T	G	R	V	T	Q	I	A	G	P	V	V	E	V	E	G	P	S	A	A	S	V	G	E	L	L	E	I	S	N	G	E	V	L	G	K	V	L	G	F	R
Posterior probability	0.285	0.982	0.504	0.547	0.458	0.3	0.597	0.266	0.982	0.954	0.522	0.771	0.497	0.722	0.183	0.979	0.336	0.209	0.364	0.414	0.392	0.366	0.75	0.843	0.372	0.448	0.585	0.468	0.213	0.578	0.982	0.716	0.427	0.739	0.721	0.251	0.614	0.469	0.205	0.404	0.187
Sequence	T	G	K	V	T	K	I	K	G	P	V	V	E	V	Q	G	G	Q	G	A	E	V	G	E	V	V	E	I	G	D	G	K	T	L	G	E	V	L	G	F	K
Posterior probability	0.689	0.979	0.458	0.518	0.476	0.421	0.519	0.5	0.986	0.962	0.33	0.535	0.442	0.713	0.282	0.999	0.324	0.439	0.315	0.506	0.402	0.279	0.687	0.651	0.424	0.625	0.502	0.453	0.23	0.712	0.994	0.537	0.252	0.646	0.695	0.165	0.595	0.413	0.255	0.382	0.173
Sequence	T	G	R	I	T	S	I	V	G	P	V	V	E	V	S	G	A	E	A	A	S	I	Y	E	V	L	K	V	E	G	D	K	E	L	V	A	E	V	A	Q	H
Posterior probability	0.368	0.999	0.675	0.717	0.555	0.469	0.782	0.248	0.999	0.998	0.822	0.851	0.526	0.74	0.18	0.954	0.253	0.305	0.346	0.6	0.369	0.436	0.821	0.719	0.425	0.499	0.525	0.613	0.366	0.802	0.484	0.365	0.425	0.909	0.71	0.153	0.882	0.682	0.363	0.53	0.286
Sequence	T	G	K	I	T	R	I	R	G	P	V	V	E	V	K	G	G	Q	G	A	E	I	Y	E	V	V	K	V	K	G	D	K	K	L	V	A	E	V	A	Q	F
Posterior probability	0.698	0.999	0.503	0.717	0.511	0.39	0.707	0.316	0.999	0.998	0.672	0.781	0.487	0.63	0.326	0.967	0.4	0.557	0.32	0.673	0.285	0.484	0.824	0.556	0.532	0.495	0.477	0.616	0.319	0.623	0.664	0.335	0.513	0.869	0.695	0.176	0.871	0.665	0.657	0.521	0.29
Sequence	D	V	S	E	V	G	K	V	V	S	V	G	D	G	I	A	R	I	Y	G	L	D	N	V	M	A	G	E	M	L	E	F	P	N	G	V	F	G	M	A	L
Posterior probability	0.969	0.762	0.633	0.992	0.968	1	0.615	0.956	0.528	0.888	0.992	0.596	0.999	0.997	0.772	0.998	0.696	0.947	0.974	1	0.998	0.708	0.993	0.964	0.999	0.993	0.996	0.999	0.744	0.923	0.999	0.999	0.669	0.998	1	0.897	0.701	1	0.99	0.999	0.995
Sequence	L	E	E	T	G	T	V	I	K	V	G	D	G	V	A	R	V	Y	G	L	E	N	V	E	A	G	E	V	V	E	F	E	D	G	T	M	G	M	A	L	N
Posterior probability	0.359	0.459	0.998	0.868	1	0.464	0.984	0.985	0.647	0.992	0.985	0.998	0.999	0.883	0.997	0.955	0.989	0.998	1	0.996	0.936	0.997	0.989	0.699	0.616	1	0.998	0.71	0.992	0.998	0.998	0.992	0.632	1	0.755	0.506	1	0.54	0.997	0.723	0.997
Sequence	N	K	G	K	I	T	S	I	I	G	P	V	V	D	V	K	F	P	E	G	E	L	P	A	I	Y	Q	A	L	K	V	K	R	G	D	K	K	L	V	L	E
Posterior probability	0.25	0.722	1	0.578	0.979	0.555	0.899	0.96	0.465	1	1	0.99	0.991	0.99	0.991	0.6	0.996	0.999	0.629	0.976	0.572	0.989	0.998	0.346	0.738	0.902	0.443	0.992	0.987	0.879	0.769	0.526	0.819	0.825	0.611	0.99	0.452	0.996	0.981	0.988	0.998
Sequence	N	T	G	K	I	T	S	I	R	G	P	V	V	D	V	K	F	P	E	G	Q	L	P	P	I	Y	N	A	L	K	V	K	N	G	D	K	R	L	V	L	E
Posterior probability	0.505	0.495	1	0.706	0.989	0.522	0.889	0.889	0.562	1	1	0.988	0.99	0.991	0.991	0.582	0.997	0.999	0.376	0.987	0.627	0.99	0.998	0.432	0.759	0.913	0.565	0.993	0.987	0.881	0.774	0.727	0.442	0.616	0.698	0.987	0.475	0.996	0.986	0.984	0.998
Sequence	M	T	T	E	M	T	Q	Q	T	G	R	I	E	R	I	A	G	P	V	V	Q	A	S	G	M	K	G	A	K	M	Y	E	V	V	R	V	G	E	E	Q	L
Posterior probability	0.784	0.432	0.5	0.922	0.973	0.942	0.559	0.818	0.537	1	0.956	0.987	0.57	0.493	0.955	0.597	1	1	0.989	0.99	0.383	0.699	0.716	0.999	0.785	0.556	0.543	0.71	0.719	0.981	0.999	0.977	0.986	0.977	0.93	0.986	1	0.795	0.668	0.474	0.997
Sequence	M	T	T	M	M	T	Q	Q	T	G	R	I	E	R	I	S	G	P	V	V	K	A	S	G	M	R	G	A	K	M	Y	E	V	V	R	V	G	E	E	R	L
Posterior probability	0.79	0.289	0.577	0.469	0.99	0.912	0.623	0.849	0.72	1	0.527	0.987	0.524	0.703	0.984	0.72	1	1	0.986	0.988	0.842	0.705	0.665	0.999	0.797	0.502	0.634	0.832	0.769	0.982	0.999	0.963	0.987	0.981	0.977	0.985	0.999	0.848	0.678	0.437	0.996
Sequence	M	T	T	E	G	G	V	Q	Y	S	K	I	A	E	I	K	G	P	L	V	V	V	D	D	V	E	N	A	A	F	D	E	L	V	E	I	E	T	T	E	G
Posterior probability	1	1	0.925	1	1	1	1	1	1	1	1	1	1	1	1	1	1	1	1	1	0.998	1	1	1	1	1	1	1	1	1	1	1	1	1	1	1	1	1	0.896	1	1
Sequence	M	M	M	S	S	M	S	G	V	E	Y	S	K	I	A	E	I	K	G	P	L	M	V	V	D	G	V	S	G	A	A	Y	D	E	L	V	E	I	E	T	A
Posterior probability	0.947	0.999	0.751	0.441	0.449	0.387	0.864	0.993	0.86	0.983	0.999	0.534	0.685	0.923	0.576	0.998	0.994	0.982	1	1	0.998	0.94	0.899	0.961	0.657	1	0.958	0.711	0.711	0.813	0.461	0.562	0.997	0.999	0.979	0.997	0.999	0.639	0.999	0.977	0.685

Sequence	R	D	D	R	V	V	V	M	V	L	E	D	T	T	G	I	A	T	E	G	S	K	V	V	A	T	G	K	A	L	E	V	P	V	G	E	E	L	L	G	R
Posterior probability	0.284	0.477	0.884	0.316	0.726	0.389	0.343	0.524	0.524	0.717	0.586	0.92	0.68	0.707	0.973	0.406	0.457	0.354	0.276	0.987	0.55	0.405	0.629	0.735	0.461	0.434	0.947	0.765	0.763	0.658	0.681	0.781	1	0.834	0.997	0.701	0.976	0.821	0.949	1	0.987

Sequence	K	G	D	N	V	V	V	M	V	F	E	D	S	T	G	I	Q	T	A	G	S	K	A	V	A	M	G	K	A	L	E	V	P	V	G	E	G	L	L	G	R
Posterior probability	0.203	0.886	0.698	0.585	0.704	0.449	0.348	0.434	0.539	0.573	0.698	0.794	0.328	0.716	0.986	0.398	0.411	0.446	0.331	0.986	0.552	0.69	0.542	0.629	0.495	0.327	0.948	0.509	0.658	0.776	0.684	0.561	0.999	0.829	0.965	0.762	0.554	0.861	0.957	1	0.987

Sequence	D	D	K	V	V	V	M	V	L	E	D	T	T	G	I	A	T	E	G	S	K	V	K	A	T	G	K	V	M	E	V	P	V	G	E	E	L	L	G	R	V
Posterior probability	0.469	0.869	0.311	0.705	0.382	0.447	0.44	0.534	0.708	0.579	0.887	0.651	0.686	0.978	0.446	0.352	0.265	0.299	0.995	0.465	0.457	0.606	0.577	0.333	0.46	0.979	0.739	0.473	0.485	0.79	0.695	1	0.829	0.98	0.713	0.968	0.768	0.885	0.999	0.981	0.77

Sequence	G	D	N	V	V	V	M	V	F	E	D	S	T	G	I	Q	T	A	G	S	K	A	K	A	T	G	R	A	L	E	V	P	V	G	E	G	L	L	G	R	V
Posterior probability	0.412	0.714	0.583	0.686	0.452	0.461	0.37	0.552	0.635	0.695	0.737	0.321	0.699	0.994	0.43	0.317	0.375	0.386	0.995	0.573	0.766	0.6	0.422	0.375	0.29	0.981	0.527	0.478	0.683	0.761	0.467	1	0.828	0.952	0.759	0.53	0.837	0.906	0.999	0.983	0.737

Sequence	L	G	D	N	T	V	T	V	V	V	M	D	T	T	G	G	L	T	E	G	G	A	V	A	A	T	G	A	P	L	S	V	P	V	G	P	E	L	L	G	R
Posterior probability	0.715	0.764	0.878	0.511	0.526	0.595	0.282	0.29	0.514	0.386	0.275	0.879	0.512	0.921	0.452	0.887	0.78	0.646	0.215	0.323	0.926	0.24	0.854	0.346	0.214	0.638	0.934	0.888	0.974	0.607	0.544	0.854	0.991	0.722	0.999	0.672	0.915	0.669	0.952	0.997	0.936

Sequence	L	G	D	N	T	V	T	V	V	V	M	D	S	T	G	G	L	T	G	G	Q	A	V	A	A	T	G	A	P	L	S	V	P	V	G	P	G	L	L	G	R
Posterior probability	0.708	0.939	0.743	0.688	0.429	0.531	0.273	0.318	0.45	0.324	0.248	0.784	0.448	0.913	0.511	0.875	0.827	0.658	0.375	0.912	0.357	0.365	0.834	0.387	0.216	0.601	0.929	0.784	0.976	0.693	0.446	0.76	0.991	0.731	0.996	0.643	0.539	0.732	0.968	0.999	0.944

Sequence	N	L	E	E	D	S	V	G	V	I	L	L	G	D	D	K	G	I	K	E	G	D	T	V	K	R	T	G	R	I	M	E	V	P	V	G	E	E	L	I	G
Posterior probability	0.999	0.998	0.998	0.998	0.993	0.936	0.997	0.999	0.493	0.556	0.652	0.987	1	0.944	0.762	0.384	0.841	0.97	0.998	0.999	1	0.594	0.775	0.995	0.995	0.997	0.993	0.984	0.803	0.838	0.986	0.884	0.996	1	0.997	1	0.991	0.828	0.993	0.958	1

Sequence	L	E	E	D	N	V	G	V	V	L	L	G	E	G	R	G	I	K	E	G	S	K	A	K	R	T	G	R	I	L	E	V	P	V	G	E	G	L	I	G	R
Posterior probability	0.996	0.997	0.997	0.999	0.992	0.995	0.998	0.763	0.978	0.586	0.87	0.999	0.594	0.987	0.941	0.996	0.988	0.527	0.997	1	0.872	0.839	0.572	0.997	0.997	0.997	1	0.981	0.966	0.602	0.651	0.587	1	0.994	1	0.59	0.634	0.998	0.989	1	0.999

Sequence	V	A	Q	H	L	G	D	N	T	V	R	C	V	A	M	D	S	T	D	G	L	T	R	G	Q	E	V	V	D	T	G	A	P	I	S	V	P	V	G	P	E
Posterior probability	0.986	0.463	0.996	0.978	0.911	0.999	0.998	0.767	0.609	0.988	0.994	0.659	0.741	0.991	0.996	0.995	0.558	0.998	0.994	0.999	0.995	0.693	0.994	1	0.474	0.604	0.991	0.677	0.992	0.994	1	0.907	1	0.981	0.62	0.991	1	0.987	1	0.676	0.998

Sequence	V	A	Q	H	L	G	D	N	T	V	R	C	I	A	M	D	S	T	D	G	L	T	R	G	Q	E	V	V	D	T	G	A	P	I	T	V	P	V	G	P	E
Posterior probability	0.988	0.983	0.996	0.982	0.947	1	0.997	0.861	0.61	0.991	0.995	0.694	0.63	0.993	0.997	0.997	0.732	0.998	0.995	0.999	0.997	0.699	0.995	1	0.693	0.659	0.992	0.763	0.993	0.995	1	0.806	1	0.984	0.571	0.99	1	0.989	1	0.647	0.995

Sequence	I	G	E	V	I	R	L	G	E	D	T	A	T	I	Q	V	Y	E	E	T	S	G	L	T	P	G	E	P	V	E	A	T	G	A	P	L	S	V	E	L	G
Posterior probability	0.919	0.998	0.998	0.578	0.971	0.541	0.798	0.712	0.425	0.832	0.902	0.675	0.995	0.646	0.996	0.985	0.996	0.991	0.967	0.998	0.976	1	0.961	0.893	0.999	1	0.995	0.999	0.99	0.877	0.496	0.996	0.999	0.907	0.97	0.994	0.993	0.986	0.994	0.989	1

Sequence	I	G	E	V	I	R	L	G	E	D	T	A	T	I	Q	V	Y	E	E	T	S	G	L	T	P	G	E	P	V	E	A	T	G	A	P	L	S	V	E	L	G
Posterior probability	0.921	0.998	0.998	0.855	0.966	0.533	0.802	0.848	0.415	0.865	0.527	0.691	0.995	0.651	0.996	0.984	0.996	0.991	0.489	0.998	0.977	1	0.995	0.891	0.999	1	0.995	0.999	0.989	0.873	0.493	0.995	0.999	0.827	0.972	0.995	0.992	0.983	0.994	0.988	1

Sequence	E	R	R	L	G	K	V	L	E	V	G	N	G	K	A	I	V	Q	V	F	E	G	T	T	G	L	S	I	S	G	T	K	A	K	F	V	G	K	V	M	E
Posterior probability	1	1	1	1	1	1	1	1	1	1	1	1	1	1	1	1	1	1	1	1	1	1	1	1	1	1	1	1	1	0.999	1	1	0.937	1	1	1	1	1	1	1	1

Sequence	D	G	E	R	R	L	G	R	V	L	E	V	S	E	G	K	A	V	V	Q	V	F	E	G	T	T	G	L	S	V	A	G	T	K	A	R	F	L	G	R	T
Posterior probability	0.831	1	0.999	0.971	0.999	0.994	1	0.538	0.997	0.942	0.998	0.993	0.68	0.589	0.999	0.576	0.998	0.968	0.995	0.999	0.996	0.999	0.999	1	0.998	0.97	1	0.992	0.939	0.641	0.554	1	0.998	0.997	0.961	0.576	0.999	0.976	1	0.579	0.99

Sequence	V	F	D	G	L	G	R	P	I	D	G	K	G	P	I	K	A	N	E	Y	R	P	I	H	R	E	P	P	N	P	M	E	R	K	P	V	K	E	P	L	Q
Posterior probability	0.749	0.595	0.957	0.743	0.944	0.999	0.564	1	0.807	0.991	0.976	0.842	0.983	0.993	0.6	0.859	0.815	0.3	0.752	0.487	0.852	0.999	0.568	0.818	0.372	0.821	0.836	0.999	0.842	0.884	0.265	0.685	0.972	0.316	0.754	0.363	0.555	0.886	0.967	0.936	0.569
Sequence	V	F	D	G	L	G	R	P	I	D	G	K	G	P	I	K	A	K	E	R	Y	P	I	H	G	A	P	P	N	P	V	E	R	K	P	V	E	E	P	L	Q
Posterior probability	0.71	0.746	0.981	0.822	0.844	0.999	0.949	1	0.881	0.991	0.832	0.833	0.978	0.832	0.473	0.811	0.871	0.387	0.79	0.958	0.585	0.997	0.568	0.737	0.564	0.246	0.978	0.999	0.833	0.782	0.3	0.652	0.977	0.321	0.571	0.348	0.349	0.895	0.792	0.77	0.533
Sequence	F	D	G	L	G	R	P	I	D	G	K	G	P	I	K	A	D	E	F	R	P	I	H	R	E	P	P	N	P	M	E	R	Q	P	V	K	E	P	L	Q	T
Posterior probability	0.594	0.918	0.744	0.949	0.999	0.468	1	0.714	0.986	0.992	0.797	0.964	0.982	0.536	0.695	0.848	0.332	0.734	0.652	0.827	0.99	0.574	0.563	0.359	0.804	0.835	0.991	0.816	0.881	0.238	0.627	0.973	0.291	0.751	0.416	0.711	0.831	0.962	0.876	0.748	0.982
Sequence	F	D	G	L	G	R	P	I	D	G	K	G	P	I	K	A	K	E	R	Y	P	I	H	G	E	P	P	N	P	V	E	R	K	P	V	E	E	P	L	Q	T
Posterior probability	0.745	0.97	0.819	0.882	0.999	0.946	1	0.858	0.987	0.947	0.794	0.962	0.684	0.442	0.658	0.893	0.373	0.827	0.885	0.452	0.96	0.53	0.537	0.564	0.257	0.973	0.992	0.818	0.781	0.34	0.593	0.977	0.404	0.587	0.383	0.26	0.845	0.798	0.737	0.72	0.985
Sequence	I	F	D	G	L	G	R	P	I	D	E	K	G	P	V	K	A	K	E	R	W	P	I	H	R	P	P	P	N	L	K	E	R	S	P	V	D	E	P	L	E
Posterior probability	0.435	0.834	0.927	0.704	0.746	0.976	0.52	1	0.745	0.946	0.615	0.719	0.943	0.984	0.548	0.923	0.75	0.464	0.513	0.864	0.895	1	0.628	0.869	0.633	0.492	0.777	1	0.453	0.472	0.204	0.833	0.809	0.348	0.742	0.169	0.399	0.904	0.946	0.962	0.413
Sequence	I	F	D	G	I	G	R	P	I	D	E	K	G	P	V	K	A	K	E	R	W	P	I	H	R	P	P	P	N	L	T	E	R	S	P	P	D	E	P	L	E
Posterior probability	0.459	0.863	0.947	0.782	0.55	0.979	0.826	1	0.794	0.953	0.481	0.742	0.941	0.831	0.444	0.897	0.816	0.554	0.448	0.973	0.489	1	0.598	0.801	0.592	0.758	0.907	1	0.487	0.543	0.297	0.844	0.875	0.309	0.591	0.202	0.478	0.901	0.797	0.908	0.354
Sequence	R	V	V	N	A	L	G	Q	P	I	D	G	K	G	P	I	N	A	K	E	F	R	P	V	E	R	K	A	P	G	V	V	D	R	Q	P	V	K	E	P	L
Posterior probability	1	0.997	0.952	0.991	0.998	0.999	1	0.848	1	0.995	1	0.999	0.999	1	0.989	0.996	0.713	0.871	0.526	0.999	0.969	0.998	0.995	0.777	0.998	0.48	0.985	0.999	1	1	0.847	0.564	0.962	1	0.743	0.882	0.996	0.878	0.999	1	0.999
Sequence	V	L	D	A	L	G	R	P	I	D	G	K	G	P	I	E	A	K	E	R	R	P	V	E	R	I	A	P	G	L	V	D	R	K	P	V	H	E	P	L	Q
Posterior probability	0.994	0.656	0.763	0.795	0.998	1	0.801	1	0.995	0.999	1	0.998	1	0.605	0.992	0.784	0.894	0.545	0.999	0.866	0.998	0.862	0.666	0.996	0.975	0.764	0.997	1	0.999	0.521	0.968	0.608	0.999	0.966	0.995	0.993	0.999	0.999	1	0.998	0.999
Sequence	T	L	G	R	M	F	D	V	L	G	E	P	I	D	E	K	G	P	V	K	A	K	K	R	W	P	I	H	R	P	P	P	S	L	S	E	Q	S	T	E	D
Posterior probability	0.893	0.998	1	0.998	0.952	0.997	0.715	0.599	0.755	1	0.506	1	0.987	0.999	0.992	0.782	0.957	0.999	0.902	0.94	0.839	0.993	0.607	0.996	0.862	1	0.985	0.999	0.995	0.517	0.749	1	0.634	0.819	0.321	0.831	0.852	0.989	0.771	0.983	0.494
Sequence	T	L	G	R	M	F	D	V	I	G	E	P	I	D	E	K	G	P	V	K	A	K	K	R	Y	P	I	H	R	P	P	P	S	L	T	E	Q	S	T	E	D
Posterior probability	0.928	0.998	1	0.999	0.891	0.998	0.688	0.694	0.831	1	0.504	1	0.983	0.999	0.995	0.806	0.96	0.993	0.744	0.931	0.886	0.994	0.853	0.999	0.545	1	0.986	0.999	0.996	0.811	0.852	1	0.521	0.968	0.484	0.845	0.87	0.991	0.826	0.992	0.537
Sequence	P	G	L	L	G	S	I	Y	D	G	I	Q	R	P	L	P	E	I	R	E	M	S	G	D	F	I	S	R	G	I	T	V	P	G	L	D	R	E	K	K	W
Posterior probability	1	0.998	0.993	0.853	0.876	0.674	0.984	0.948	0.999	0.999	0.984	0.995	0.995	1	0.99	0.999	0.677	0.625	0.935	0.996	0.701	0.985	0.999	0.996	0.997	0.983	0.704	0.996	0.999	0.636	0.799	0.944	0.711	0.921	0.749	0.945	0.585	0.81	0.995	0.986	1
Sequence	P	G	L	L	G	S	I	Y	D	G	I	Q	R	P	L	P	E	I	R	E	M	S	G	D	F	I	S	R	G	I	T	V	P	G	L	D	R	E	K	K	W
Posterior probability	1	0.999	0.995	0.968	0.994	0.861	0.983	0.94	0.999	0.999	0.986	0.995	0.997	1	0.989	0.999	0.496	0.634	0.826	0.995	0.668	0.991	0.999	0.996	0.997	0.982	0.845	0.995	0.999	0.675	0.502	0.948	0.714	0.894	0.756	0.962	0.653	0.875	0.994	0.986	1
Sequence	M	P	V	S	K	E	V	L	G	R	V	F	D	G	L	G	K	P	K	D	G	L	P	D	P	I	A	D	K	F	V	D	I	N	G	E	P	M	N	P	E
Posterior probability	1	1	1	1	0.999	1	1	1	1	1	1	1	1	1	1	1	0.845	1	1	1	1	1	1	1	1	1	1	1	1	1	0.907	1	1	1	1	1	1	1	1	1	1
Sequence	M	E	M	P	V	S	E	E	M	L	G	R	V	F	D	G	L	G	R	P	I	D	G	L	P	D	P	I	A	D	E	R	L	D	V	N	G	E	P	I	N
Posterior probability	0.968	0.999	0.953	1	0.997	0.998	0.546	0.995	0.66	0.999	1	1	0.943	1	0.991	1	0.999	1	0.992	1	0.936	1	1	0.981	1	0.797	0.987	0.994	0.999	0.495	0.77	0.264	0.976	0.999	0.55	0.999	1	0.998	1	0.563	1

Sequence	T	G	V	K	V	I	D	G	L	L	P	I	G	K	G	Q	R	I	G	I	F	G	G	S	G	V	G	K	T	V	L	L	A	Q	I	A	R	N	A	N	A
Posterior probability	0.992	1	0.592	0.536	0.907	0.885	0.995	0.776	0.806	0.448	0.999	0.506	0.635	0.787	1	0.939	0.698	0.408	0.843	0.853	0.981	0.83	0.999	0.765	0.998	0.721	0.998	0.957	0.58	0.544	0.898	0.521	0.419	0.615	0.666	0.679	0.928	0.936	0.465	0.582	0.459
Sequence	T	G	I	R	A	I	D	G	L	L	P	I	G	K	G	Q	R	V	G	I	F	G	G	S	G	V	G	K	T	T	L	L	A	Q	I	A	R	N	T	N	A
Posterior probability	0.992	1	0.578	0.559	0.662	0.846	0.995	0.726	0.866	0.652	1	0.595	0.582	0.779	1	0.931	0.638	0.521	0.806	0.828	0.98	0.828	0.999	0.743	0.998	0.723	0.998	0.955	0.641	0.537	0.894	0.483	0.434	0.648	0.604	0.71	0.931	0.933	0.427	0.512	0.641
Sequence	G	V	K	V	I	D	G	L	L	P	I	G	R	G	Q	R	I	G	I	F	G	G	S	G	V	G	K	T	V	L	A	A	Q	I	A	R	N	A	N	G	D
Posterior probability	0.999	0.563	0.576	0.852	0.859	0.989	0.778	0.779	0.374	0.991	0.534	0.633	0.502	0.999	0.956	0.68	0.303	0.414	0.837	0.951	0.831	0.982	0.728	0.979	0.542	0.982	0.883	0.586	0.47	0.842	0.466	0.49	0.594	0.619	0.635	0.898	0.894	0.497	0.486	0.505	0.28
Sequence	G	I	K	A	I	D	G	L	L	P	I	G	K	G	Q	R	V	G	I	F	G	G	S	G	V	G	K	T	T	L	A	A	Q	I	A	R	N	T	N	K	E
Posterior probability	0.999	0.606	0.482	0.711	0.838	0.99	0.732	0.815	0.611	0.992	0.586	0.582	0.516	0.999	0.951	0.626	0.451	0.432	0.824	0.958	0.825	0.986	0.721	0.984	0.587	0.98	0.891	0.635	0.53	0.852	0.445	0.489	0.629	0.585	0.674	0.912	0.898	0.333	0.432	0.166	0.201
Sequence	T	G	I	K	V	I	D	A	L	M	P	I	A	K	G	G	K	V	G	I	F	G	G	A	G	V	G	K	T	V	L	L	M	Q	L	A	R	N	A	N	A
Posterior probability	0.985	0.999	0.509	0.568	0.902	0.885	0.99	0.24	0.776	0.314	1	0.613	0.522	0.916	0.999	0.923	0.612	0.424	0.856	0.628	0.945	0.984	0.999	0.481	0.999	0.725	0.999	0.971	0.876	0.816	0.827	0.439	0.291	0.557	0.731	0.537	0.869	0.815	0.367	0.293	0.442
Sequence	T	G	I	R	V	I	D	A	L	L	P	I	A	K	G	G	K	V	G	I	F	G	G	A	G	V	G	K	T	V	L	L	M	Q	L	A	R	N	T	N	A
Posterior probability	0.987	0.999	0.592	0.515	0.789	0.877	0.991	0.258	0.817	0.404	1	0.641	0.549	0.905	0.999	0.913	0.61	0.529	0.82	0.663	0.95	0.981	0.999	0.45	0.999	0.733	0.999	0.971	0.879	0.756	0.833	0.432	0.283	0.598	0.705	0.594	0.874	0.839	0.372	0.292	0.6
Sequence	Q	T	G	I	K	A	I	D	A	M	I	P	I	G	R	G	Q	R	E	L	I	I	G	D	R	Q	T	G	K	T	A	I	A	I	D	T	I	I	N	Q	K
Posterior probability	0.995	1	1	0.996	0.999	0.998	0.997	1	0.998	0.694	0.831	1	0.996	1	0.999	1	1	0.999	0.999	0.998	0.997	0.996	1	0.999	0.988	0.997	0.999	1	0.999	0.999	0.699	0.845	0.999	0.933	0.998	0.995	0.993	0.995	0.997	0.999	0.918
Sequence	T	G	I	K	A	I	D	A	L	I	P	I	G	R	G	Q	R	E	L	I	I	G	D	R	Q	T	G	K	T	T	I	A	I	D	T	I	L	N	Q	K	R
Posterior probability	0.999	1	0.993	0.997	0.998	0.995	0.999	0.997	0.92	0.959	1	0.993	0.999	0.997	1	1	0.998	0.997	0.995	0.994	0.992	1	0.998	0.996	0.998	0.998	1	0.999	0.998	0.98	0.961	0.998	0.974	0.996	0.997	0.986	0.531	0.999	0.998	0.995	0.558
Sequence	E	I	L	E	T	G	I	K	V	I	D	L	L	A	P	I	P	K	G	G	K	I	G	L	F	G	G	A	G	V	G	K	T	V	L	I	M	E	L	I	R
Posterior probability	0.998	0.511	0.986	0.993	0.999	1	0.982	0.991	0.992	0.991	0.999	0.988	0.858	0.992	1	0.659	0.998	0.997	1	1	0.992	0.633	0.999	0.959	0.999	1	1	0.992	1	0.99	1	0.998	0.997	0.991	0.99	0.872	0.997	0.992	0.994	0.963	0.997
Sequence	E	I	L	E	T	G	I	K	V	I	D	L	L	A	P	I	P	K	G	G	K	I	G	L	F	G	G	A	G	V	G	K	T	V	L	I	M	E	L	I	R
Posterior probability	0.998	0.566	0.987	0.994	0.999	1	0.987	0.991	0.993	0.993	0.999	0.99	0.885	0.993	1	0.635	0.998	0.998	1	1	0.993	0.648	0.999	0.969	0.999	1	1	0.993	1	0.991	1	0.999	0.998	0.992	0.996	0.893	0.997	0.993	0.995	0.968	0.991
Sequence	E	F	T	P	T	V	K	V	G	D	K	V	V	G	G	D	I	L	G	E	V	P	E	T	P	I	I	E	H	R	I	M	V	P	P	G	V	K	G	K	I
Posterior probability	0.627	0.997	0.539	1	0.849	0.843	0.995	0.981	0.999	0.996	0.732	0.985	0.49	0.673	0.999	0.996	0.977	0.599	0.999	0.921	0.944	1	0.996	0.995	0.403	0.893	0.863	0.557	0.554	0.686	0.96	0.686	0.693	1	0.716	0.989	0.931	0.691	0.999	0.698	0.85
Sequence	E	F	T	P	T	V	K	V	G	D	K	V	V	G	G	D	I	I	G	E	V	P	E	T	P	I	I	E	H	K	I	M	V	P	P	G	V	K	G	K	I
Posterior probability	0.6	0.997	0.586	1	0.954	0.867	0.994	0.918	0.999	0.996	0.752	0.983	0.552	0.631	0.999	0.996	0.977	0.932	0.999	0.913	0.948	0.998	0.995	0.995	0.448	0.769	0.948	0.576	0.573	0.571	0.961	0.691	0.704	1	0.719	0.993	0.925	0.673	0.999	0.704	0.664
Sequence	Q	R	E	Y	P	K	D	F	I	Q	T	G	V	S	V	I	D	G	L	M	T	L	V	R	G	Q	K	L	P	I	F	S	G	S	G	M	S	H	N	L	L
Posterior probability	1	1	1	1	1	1	1	1	1	1	1	1	1	1	1	1	1	1	0.901	0.887	1	1	1	1	1	1	1	1	1	1	1	1	1	1	1	1	1	1	1	0.857	1
Sequence	P	E	Q	R	E	Y	P	K	D	F	I	Q	T	G	I	S	A	I	D	G	M	L	T	L	V	R	G	Q	K	L	P	I	F	S	G	S	G	L	P	H	N
Posterior probability	1	0.965	0.979	1	0.998	0.999	1	0.325	0.992	0.999	0.996	1	1	1	0.605	0.998	0.723	0.997	1	1	0.999	0.636	0.938	0.997	0.996	0.999	1	1	0.998	0.997	1	0.997	1	0.998	1	0.976	1	0.582	0.99	0.999	0.999

Sequence	D	V	S	V	F	A	A	I	G	E	R	G	R	E	V	N	E	F	I	E	H	L	E	E	S	G	A	L	K	R	T	V	L	V	V	A	T	S	D	E	P
Posterior probability	0.316	0.694	0.296	0.851	0.755	0.929	0.69	0.715	1	0.94	0.915	0.749	0.764	0.98	0.632	0.321	0.894	0.759	0.496	0.564	0.185	0.523	0.378	0.724	0.279	0.932	0.774	0.789	0.419	0.871	0.516	0.854	0.535	0.783	0.539	0.493	0.949	0.524	0.722	0.692	1
Sequence	D	V	S	V	F	A	A	I	G	E	R	G	R	E	V	N	E	F	I	E	E	L	K	E	S	G	A	L	K	R	T	V	L	V	V	S	T	S	D	E	P
Posterior probability	0.34	0.664	0.281	0.883	0.759	0.93	0.692	0.674	1	0.94	0.911	0.747	0.692	0.981	0.59	0.282	0.886	0.737	0.503	0.649	0.247	0.529	0.435	0.796	0.344	0.946	0.798	0.755	0.437	0.91	0.588	0.859	0.588	0.683	0.519	0.436	0.928	0.487	0.674	0.652	1
Sequence	D	V	V	C	V	F	A	A	I	G	E	R	G	S	E	V	N	E	F	I	K	S	L	E	E	S	G	A	L	K	R	T	V	L	V	V	A	T	A	D	D
Posterior probability	0.528	0.146	0.647	0.377	0.758	0.727	0.882	0.793	0.743	0.999	0.736	0.715	0.382	0.402	0.933	0.623	0.202	0.612	0.746	0.398	0.421	0.225	0.695	0.679	0.836	0.312	0.979	0.848	0.753	0.411	0.858	0.521	0.764	0.517	0.697	0.447	0.473	0.923	0.605	0.716	0.527
Sequence	A	A	V	C	V	F	A	A	I	G	E	R	G	R	E	V	N	E	F	I	E	Q	L	K	E	S	G	A	L	K	R	T	V	L	V	V	S	T	A	D	D
Posterior probability	0.485	0.138	0.635	0.367	0.831	0.736	0.889	0.784	0.706	0.999	0.765	0.738	0.419	0.374	0.945	0.586	0.201	0.643	0.73	0.451	0.501	0.2	0.676	0.411	0.869	0.37	0.982	0.859	0.729	0.431	0.894	0.584	0.79	0.567	0.577	0.455	0.399	0.879	0.609	0.677	0.517
Sequence	G	V	S	V	F	A	G	V	G	E	R	G	R	E	V	N	E	L	L	E	E	M	K	E	S	G	V	L	E	R	T	V	L	V	V	G	T	S	N	E	P
Posterior probability	0.267	0.423	0.39	0.88	0.743	0.88	0.923	0.657	0.999	0.976	0.967	0.717	0.756	0.984	0.293	0.509	0.926	0.543	0.484	0.541	0.809	0.464	0.373	0.647	0.344	0.923	0.554	0.617	0.547	0.751	0.858	0.877	0.849	0.719	0.327	0.533	0.816	0.432	0.802	0.784	1
Sequence	D	V	S	V	F	A	G	V	G	E	R	G	R	E	V	N	E	L	L	E	E	M	K	E	S	G	V	L	E	R	T	V	L	V	V	G	T	S	N	E	P
Posterior probability	0.292	0.435	0.352	0.895	0.741	0.887	0.909	0.645	0.999	0.976	0.964	0.725	0.694	0.985	0.308	0.436	0.92	0.513	0.505	0.617	0.803	0.44	0.547	0.745	0.388	0.935	0.533	0.601	0.595	0.805	0.861	0.878	0.849	0.663	0.329	0.344	0.833	0.434	0.789	0.756	1
Sequence	G	Q	D	V	I	C	I	Y	V	A	I	G	Q	K	Q	S	T	V	A	Q	V	V	K	T	L	E	E	H	G	A	M	E	Y	T	I	V	V	A	A	T	A
Posterior probability	1	0.997	0.844	0.996	0.993	1	0.995	0.999	0.997	0.999	0.997	1	0.999	0.998	0.556	0.998	0.996	0.997	0.998	0.999	0.994	0.987	0.923	0.739	0.999	0.999	0.988	0.995	1	0.999	0.999	0.601	0.999	0.988	0.952	0.995	0.979	0.626	0.999	0.56	0.999
Sequence	E	G	V	I	C	V	Y	C	A	I	G	Q	K	R	S	T	V	A	Q	V	I	Q	K	L	K	E	H	G	A	M	E	Y	T	I	V	I	A	A	T	A	S
Posterior probability	0.646	0.916	0.993	0.977	1	0.536	0.998	0.487	0.999	0.994	1	0.998	0.995	0.88	0.996	0.997	0.994	0.996	0.998	0.718	0.526	0.586	0.633	0.997	0.601	0.994	0.999	1	0.999	0.998	0.682	0.998	0.997	0.867	0.99	0.542	0.988	0.996	0.918	0.998	0.995
Sequence	N	I	A	Y	E	H	K	G	F	S	V	F	A	G	V	G	E	R	S	R	E	G	N	E	L	W	L	E	M	K	E	S	G	V	L	D	N	T	V	L	V
Posterior probability	0.876	0.965	0.987	0.353	0.995	0.999	0.905	0.997	0.995	0.988	0.991	0.996	0.998	1	0.986	1	0.999	0.999	0.557	0.997	0.999	0.995	0.996	0.86	0.992	0.653	0.99	0.997	0.995	0.895	0.739	0.674	1	0.987	0.53	0.464	0.857	0.997	0.962	0.996	0.987
Sequence	N	I	A	I	E	H	K	G	F	S	V	F	A	G	V	G	E	R	S	R	E	G	N	E	L	W	L	E	M	K	E	S	G	V	L	D	N	T	V	L	V
Posterior probability	0.9	0.972	0.989	0.457	0.995	0.999	0.803	0.998	0.996	0.989	0.992	0.996	0.998	1	0.988	1	0.999	0.999	0.512	0.997	0.999	0.996	0.996	0.833	0.993	0.693	0.991	0.997	0.996	0.956	0.832	0.727	1	0.989	0.487	0.83	0.812	0.998	0.955	0.997	0.987
Sequence	E	E	I	A	K	K	G	E	Y	T	V	E	E	T	I	A	K	I	K	T	D	N	G	D	K	A	D	I	K	M	P	M	Q	R	W	P	V	R	K	P	R
Posterior probability	0.37	0.957	0.939	0.712	0.934	0.384	0.999	0.545	0.997	0.995	0.973	0.529	0.731	0.982	0.953	0.688	0.914	0.676	0.435	0.594	0.261	0.399	0.999	0.833	0.719	0.355	0.784	0.602	0.949	0.992	0.092	0.631	0.99	0.991	0.966	1	0.981	0.995	0.562	0.777	0.995
Sequence	T	E	I	A	K	K	G	E	Y	T	V	E	D	T	I	A	T	I	E	T	D	N	G	D	K	H	D	I	K	M	M	Q	R	W	P	V	R	K	P	R	P
Posterior probability	0.557	0.987	0.94	0.684	0.874	0.379	0.999	0.934	0.997	0.995	0.973	0.542	0.68	0.987	0.955	0.695	0.678	0.69	0.478	0.57	0.378	0.491	0.999	0.789	0.717	0.226	0.776	0.493	0.947	0.992	0.602	0.99	0.911	0.903	1	0.981	0.994	0.41	0.877	0.994	1
Sequence	A	A	Q	I	A	R	Q	A	S	V	V	G	T	S	D	D	F	A	V	V	F	A	A	I	G	V	Q	Y	S	E	A	E	Y	F	R	R	S	L	E	E	S
Posterior probability	1	1	1	1	1	1	1	1	1	1	1	1	1	0.97	1	1	1	1	1	1	1	1	1	1	1	1	1	1	1	1	1	1	1	1	1	1	1	1	1	1	1
Sequence	I	L	A	A	Q	I	A	R	Q	A	T	V	V	G	T	E	E	E	F	A	V	V	F	A	A	I	G	V	Q	Y	S	E	A	Q	Y	F	R	R	S	L	E
Posterior probability	0.462	0.999	0.999	0.999	0.999	0.996	0.999	0.999	0.999	0.998	0.557	0.995	0.897	1	0.522	0.568	0.986	0.611	0.999	0.998	0.996	0.943	0.999	0.999	0.999	0.92	1	0.976	0.962	0.462	0.491	1	0.999	0.625	0.985	1	0.635	0.954	0.992	0.978	0.999

Sequence	P	A	A	R	F	K	A	A	Y	V	G	V	T	I	A	E	Y	F	R	D	Q	G	K	D	V	L	L	M	M	D	N	L	T	R	F	A	Q	A	L	R	E
Posterior probability	0.895	0.318	0.525	0.978	0.406	0.472	0.526	0.754	0.713	0.594	0.704	0.378	0.957	0.441	0.968	0.992	0.997	0.992	0.98	0.971	0.868	1	0.925	0.537	0.924	0.931	0.821	0.367	0.347	0.982	0.344	0.491	0.758	0.895	0.667	0.988	0.413	0.991	0.319	0.982	0.986
Sequence	A	V	A	R	Y	R	A	A	Y	T	A	V	T	M	A	E	Y	F	R	D	Q	G	K	D	V	L	L	L	F	D	N	I	T	R	F	A	Q	A	L	R	E
Posterior probability	0.9	0.412	0.869	0.98	0.769	0.433	0.609	0.728	0.667	0.586	0.836	0.464	0.884	0.413	0.986	0.993	0.997	0.992	0.98	0.974	0.905	1	0.817	0.777	0.927	0.868	0.826	0.404	0.641	0.982	0.363	0.592	0.728	0.891	0.658	0.987	0.462	0.992	0.505	0.982	0.993
Sequence	P	P	V	A	R	F	I	A	P	Y	V	G	V	T	I	A	E	Y	F	R	D	Q	G	K	H	V	L	L	V	I	D	D	L	T	R	Y	A	E	A	L	R
Posterior probability	1	0.483	0.318	0.436	0.929	0.388	0.342	0.552	0.844	0.78	0.578	0.709	0.355	0.892	0.379	0.898	0.985	0.993	0.962	0.936	0.915	0.733	0.999	0.882	0.662	0.867	0.941	0.485	0.34	0.294	0.932	0.654	0.546	0.728	0.657	0.587	0.979	0.354	0.981	0.37	0.979
Sequence	P	A	V	A	R	Y	L	A	P	Y	T	A	V	T	M	A	E	Y	F	R	D	Q	G	K	H	V	L	L	I	F	D	D	I	T	R	Y	A	E	A	L	R
Posterior probability	1	0.926	0.403	0.732	0.941	0.751	0.263	0.607	0.828	0.74	0.503	0.827	0.446	0.905	0.386	0.966	0.987	0.995	0.966	0.942	0.925	0.854	0.999	0.723	0.495	0.882	0.899	0.56	0.368	0.611	0.938	0.636	0.445	0.711	0.684	0.557	0.981	0.401	0.984	0.524	0.98
Sequence	P	A	A	R	F	R	A	A	Y	T	G	V	T	M	A	E	Y	F	R	D	Q	G	K	D	V	L	L	M	I	D	N	I	S	R	F	A	Q	A	L	R	E
Posterior probability	0.906	0.637	0.883	0.984	0.506	0.454	0.489	0.778	0.637	0.649	0.928	0.398	0.976	0.596	0.978	0.987	0.994	0.99	0.984	0.985	0.72	0.999	0.843	0.904	0.909	0.871	0.929	0.399	0.416	0.987	0.546	0.519	0.406	0.962	0.733	0.949	0.582	0.984	0.324	0.882	0.985
Sequence	A	A	A	R	Y	R	A	A	Y	T	A	V	T	M	A	E	Y	F	R	D	Q	G	K	D	V	L	L	M	I	D	N	I	T	R	F	A	Q	A	L	R	E
Posterior probability	0.652	0.687	0.954	0.985	0.598	0.547	0.536	0.774	0.618	0.787	0.708	0.413	0.955	0.702	0.985	0.988	0.995	0.99	0.985	0.986	0.774	0.999	0.792	0.943	0.916	0.838	0.926	0.358	0.3	0.988	0.543	0.623	0.427	0.958	0.716	0.954	0.556	0.986	0.484	0.904	0.988
Sequence	S	D	P	A	A	L	Q	Y	I	A	P	Y	A	G	C	A	M	G	E	Y	F	R	D	K	G	K	H	A	L	V	V	Y	D	D	L	S	K	H	A	V	A
Posterior probability	0.998	0.999	1	0.999	0.885	0.541	0.999	0.994	0.785	0.999	1	1	0.744	1	0.533	0.95	0.998	0.999	1	0.996	1	1	0.999	0.546	0.973	0.953	1	0.998	0.999	0.705	0.975	0.998	1	0.999	0.998	0.997	0.998	0.999	1	0.533	1
Sequence	D	P	A	A	L	Q	Y	L	A	P	Y	A	A	V	T	M	A	E	Y	F	R	D	N	G	R	H	A	L	I	I	Y	D	D	L	S	K	H	A	V	A	Y
Posterior probability	0.998	1	0.999	0.99	0.929	0.997	0.999	0.938	0.998	1	0.999	0.484	0.644	0.982	0.939	0.588	0.737	0.999	0.999	0.999	0.999	0.999	0.588	0.999	0.678	0.767	0.997	0.998	0.91	0.557	0.997	0.999	0.999	0.996	0.99	0.996	0.867	0.999	0.957	0.999	0.997
Sequence	F	G	Q	M	N	E	P	P	G	A	R	F	R	V	A	L	T	G	L	T	M	A	E	Y	F	R	D	E	E	G	K	D	V	L	L	F	I	D	N	I	F
Posterior probability	0.816	0.998	0.995	0.996	0.997	0.996	1	1	0.996	0.998	0.999	0.994	0.993	0.985	0.885	0.985	0.8	0.984	0.455	0.999	0.996	0.999	0.999	1	0.999	0.999	0.999	0.989	0.959	1	0.958	0.998	0.992	0.996	0.997	0.995	0.981	0.999	0.996	0.984	0.995
Sequence	Y	G	Q	M	N	E	P	P	G	A	R	F	R	V	A	L	T	A	L	T	M	A	E	Y	F	R	D	E	E	G	K	D	V	L	L	F	I	D	N	I	F
Posterior probability	0.552	0.998	0.996	0.997	0.998	0.997	1	0.999	0.996	0.999	0.999	0.991	0.995	0.988	0.933	0.988	0.883	0.72	0.476	0.999	0.981	0.999	0.999	1	0.999	0.999	0.999	0.969	0.974	1	0.986	0.999	0.994	0.996	0.998	0.996	0.983	0.999	0.996	0.988	0.996
Sequence	P	F	K	E	R	L	P	P	D	T	P	L	I	T	G	Q	R	V	I	D	T	F	M	P	I	A	K	G	G	T	G	A	I	P	G	G	F	G	T	G	K
Posterior probability	1	0.58	0.979	0.968	0.869	0.522	0.954	0.276	0.705	0.41	1	0.998	0.8	0.999	1	0.996	0.994	0.962	0.968	0.999	0.993	0.985	0.641	1	0.748	0.996	0.998	1	1	0.994	0.634	0.993	0.988	0.999	1	1	0.996	1	0.995	1	0.998
Sequence	Y	K	E	R	L	P	P	D	V	P	L	I	T	G	Q	R	V	I	D	T	F	M	P	I	A	K	G	G	T	G	A	I	P	G	G	F	G	T	G	K	T
Posterior probability	0.503	0.979	0.979	0.988	0.547	0.944	0.324	0.623	0.483	1	0.997	0.846	0.999	1	0.996	0.994	0.968	0.983	0.999	0.992	0.985	0.589	1	0.76	0.995	0.997	1	1	0.994	0.603	0.992	0.987	0.999	1	1	0.996	1	0.995	1	0.998	0.998
Sequence	G	A	L	K	R	S	V	L	F	L	N	T	A	D	D	P	A	I	E	R	I	I	T	P	R	V	A	L	T	V	A	E	Y	L	A	F	D	L	G	M	H
Posterior probability	1	1	1	1	1	1	1	1	1	1	1	1	1	1	1	1	1	1	1	1	1	1	1	1	1	1	1	1	1	1	1	1	1	1	1	1	1	1	1	1	1
Sequence	E	S	G	A	L	K	R	S	V	L	F	L	N	L	A	D	D	P	A	I	E	R	I	V	T	P	R	V	A	L	T	L	A	E	Y	L	A	F	E	Q	G
Posterior probability	0.999	0.962	1	0.999	0.963	0.981	0.999	0.945	0.903	0.954	0.999	0.994	0.999	0.997	0.999	0.999	0.997	1	0.999	0.989	0.999	1	0.995	0.542	0.998	1	0.999	0.855	0.999	0.997	0.999	0.612	1	1	1	0.998	0.998	0.998	0.633	0.857	1

Sequence	I	S	L	A	L	G	E	P	P	G	R	K	G	Y	P	P	S	V	F	T	E	L	S	K	L	L	E	R	A	G	K	T	N	R	K	G	S	I	T	A	L
Posterior probability	0.773	0.874	0.487	0.566	0.788	0.963	0.75	0.845	1	0.518	0.237	0.683	0.998	0.997	0.997	0.861	0.512	0.378	0.853	0.467	0.411	0.826	0.421	0.461	0.973	0.54	0.992	0.992	0.958	0.975	0.43	0.335	0.328	0.498	0.736	1	0.974	0.766	0.992	0.436	0.398
Sequence	I	S	A	A	L	G	E	P	P	G	R	K	G	Y	P	P	S	L	F	T	E	L	A	K	L	L	E	R	A	G	R	T	K	S	L	G	S	I	T	A	L
Posterior probability	0.423	0.896	0.393	0.542	0.952	0.958	0.742	0.782	1	0.73	0.263	0.441	0.998	0.997	1	0.823	0.445	0.363	0.827	0.543	0.353	0.85	0.7	0.587	0.936	0.45	0.993	0.993	0.932	0.975	0.453	0.373	0.624	0.247	0.198	0.217	0.238	0.798	0.992	0.467	0.312
Sequence	E	I	S	L	A	L	G	E	P	P	G	R	K	G	Y	P	G	S	L	F	T	E	L	S	K	L	L	E	R	A	G	K	L	N	G	R	K	G	S	I	T
Posterior probability	0.959	0.775	0.884	0.473	0.549	0.79	0.579	0.714	0.843	1	0.84	0.603	0.666	0.98	0.994	0.999	0.74	0.378	0.377	0.81	0.492	0.372	0.811	0.581	0.413	0.964	0.51	0.985	0.985	0.962	0.96	0.682	0.522	0.451	0.27	0.531	0.718	0.999	0.952	0.635	0.982
Sequence	E	I	S	A	A	L	G	E	P	P	G	R	K	G	Y	P	G	S	L	F	T	E	L	A	K	L	L	E	R	A	G	R	V	K	G	K	K	G	S	I	T
Posterior probability	0.986	0.42	0.901	0.387	0.532	0.918	0.617	0.716	0.782	1	0.903	0.586	0.484	0.984	0.995	1	0.721	0.347	0.338	0.792	0.56	0.325	0.836	0.69	0.51	0.89	0.43	0.987	0.987	0.945	0.963	0.506	0.274	0.629	0.277	0.557	0.825	0.999	0.953	0.704	0.985
Sequence	I	S	A	L	L	G	E	M	P	G	E	E	G	Y	P	P	T	L	A	T	E	L	A	E	L	Y	E	R	A	G	R	T	K	R	K	G	S	I	T	A	V
Posterior probability	0.564	0.931	0.743	0.354	0.923	0.983	0.548	0.708	1	0.465	0.547	0.379	0.999	0.994	0.974	0.873	0.344	0.803	0.769	0.591	0.449	0.665	0.658	0.718	0.9	0.341	0.987	0.987	0.742	0.889	0.275	0.503	0.294	0.638	0.558	0.999	0.961	0.809	0.985	0.397	0.662
Sequence	I	S	A	L	L	G	E	M	P	G	E	E	G	Y	P	P	T	L	A	T	E	L	A	E	L	Y	E	R	A	G	R	T	K	K	K	G	S	I	T	A	V
Posterior probability	0.484	0.939	0.756	0.361	0.969	0.978	0.582	0.694	1	0.658	0.522	0.372	0.999	0.995	0.982	0.837	0.297	0.775	0.753	0.614	0.356	0.714	0.799	0.707	0.89	0.399	0.988	0.989	0.772	0.909	0.499	0.467	0.661	0.482	0.7	0.999	0.963	0.82	0.987	0.417	0.617
Sequence	Y	R	Q	I	S	L	L	L	R	R	P	P	G	R	E	A	Y	P	G	D	V	F	Y	L	H	S	R	L	L	E	R	A	A	K	L	S	D	E	K	G	G
Posterior probability	0.999	1	0.505	0.898	0.999	0.998	0.737	0.999	0.999	0.998	1	1	1	0.999	0.999	0.998	0.998	1	1	0.999	0.987	0.999	0.999	0.987	0.999	0.998	0.998	0.999	0.998	1	1	1	0.97	0.999	0.997	0.573	0.999	0.997	0.783	1	0.742
Sequence	R	E	M	S	L	L	L	R	R	P	P	G	R	E	A	Y	P	G	D	V	F	Y	L	H	S	R	L	L	E	R	A	A	K	L	N	D	E	L	G	A	G
Posterior probability	0.999	0.985	0.996	0.998	0.996	0.96	0.998	0.997	0.996	1	1	1	0.998	0.997	0.996	1	1	1	0.997	0.991	0.999	0.998	0.994	0.999	0.995	0.997	0.998	0.997	0.999	0.999	0.999	0.994	0.996	0.991	0.906	0.998	0.997	0.993	0.999	0.967	1
Sequence	R	F	A	Q	A	G	S	E	V	S	A	L	L	G	R	M	P	S	E	V	G	Y	Q	P	T	L	A	T	E	M	A	E	L	Q	E	R	I	T	S	T	R
Posterior probability	0.999	0.996	0.765	0.996	0.999	0.996	0.99	0.999	0.978	0.997	0.996	0.991	0.997	1	0.989	0.997	1	0.992	0.583	0.986	1	1	0.995	1	0.992	0.995	0.996	0.994	0.719	0.993	0.602	0.979	0.997	0.994	0.999	0.999	0.958	0.982	0.989	0.995	0.502
Sequence	R	F	A	Q	A	G	S	E	V	S	A	L	L	G	R	M	P	S	E	V	G	Y	Q	P	T	L	A	T	D	M	A	E	L	Q	E	R	I	T	S	T	K
Posterior probability	0.999	0.996	0.737	0.996	0.999	0.997	0.991	0.999	0.981	0.998	0.997	0.992	0.998	1	0.99	0.998	1	0.991	0.556	0.988	1	1	0.996	1	0.993	0.996	0.997	0.995	0.591	0.994	0.627	0.982	0.997	0.994	0.999	0.999	0.964	0.985	0.991	0.996	0.421
Sequence	T	V	T	L	H	Q	L	A	K	W	S	D	A	Q	I	V	V	Y	V	G	C	G	E	R	G	N	E	M	T	E	V	L	E	E	F	P	K	L	K	D	P
Posterior probability	0.998	0.99	0.983	0.698	0.998	0.997	0.873	0.996	0.989	0.999	0.598	0.995	0.995	0.64	0.603	0.986	0.99	0.995	0.916	1	0.999	1	0.999	0.999	0.999	0.996	0.999	0.996	0.993	0.986	0.861	0.993	0.98	0.996	0.839	0.999	0.467	0.991	0.872	0.996	1
Sequence	V	T	L	H	Q	L	A	K	W	S	D	A	Q	I	V	V	Y	V	G	C	G	E	R	G	N	E	M	T	E	V	L	E	E	F	P	H	L	K	D	P	R
Posterior probability	0.988	0.983	0.686	0.998	0.997	0.876	0.996	0.988	0.999	0.657	0.994	0.996	0.405	0.646	0.985	0.99	0.994	0.777	1	0.999	1	0.999	0.998	0.999	0.995	0.999	0.996	0.993	0.988	0.862	0.993	0.983	0.997	0.844	0.999	0.316	0.99	0.405	0.996	1	0.637
Sequence	V	L	V	V	I	T	D	M	T	N	Y	A	E	A	L	R	E	I	S	A	A	R	E	E	V	P	G	R	K	G	Y	P	G	Y	L	Y	T	D	L	S	T
Posterior probability	1	1	1	0.875	0.877	1	1	1	1	1	1	1	1	1	1	1	1	1	1	1	1	1	1	1	1	1	1	1	1	1	1	1	1	1	1	1	1	1	1	1	1
Sequence	M	H	V	L	V	I	L	T	D	M	T	N	Y	A	E	A	L	R	E	I	S	A	A	R	E	E	V	P	G	R	K	G	Y	P	G	Y	M	Y	T	D	L
Posterior probability	0.997	1	0.997	0.999	0.99	0.996	0.961	0.998	0.999	0.977	0.999	0.999	0.999	0.991	0.999	1	0.998	1	1	0.948	0.999	0.999	0.999	0.999	0.998	0.999	0.996	1	1	0.999	0.995	1	1	1	1	0.999	0.582	0.999	0.996	0.998	0.999

Sequence	P	T	V	S	V	P	G	D	D	L	T	E	P	I	P	D	T	V	L	S	I	L	D	G	H	I	V	L	S	R	D	L	A	N	R	G	I	Y	P	A	I
Posterior probability	0.411	0.357	0.602	0.244	0.765	0.865	0.765	0.955	0.995	0.653	0.586	0.638	1	0.618	0.95	0.733	0.292	0.519	0.29	0.613	0.795	0.606	0.936	0.976	0.601	0.86	0.769	0.963	0.505	0.951	0.754	0.969	0.6	0.466	0.81	0.999	0.558	0.984	1	0.965	0.769
Sequence	P	V	V	S	V	P	G	D	D	L	T	E	P	V	P	D	T	T	L	A	I	L	D	G	H	I	V	L	S	R	D	L	A	N	R	G	I	Y	P	A	I
Posterior probability	0.456	0.302	0.572	0.285	0.747	0.914	0.737	0.951	0.995	0.39	0.617	0.74	1	0.552	0.961	0.7	0.3	0.459	0.32	0.419	0.813	0.552	0.924	0.971	0.563	0.816	0.801	0.986	0.688	0.938	0.788	0.969	0.663	0.231	0.651	0.999	0.531	0.983	1	0.967	0.768
Sequence	A	L	P	V	I	S	V	P	G	D	D	L	T	E	P	I	P	D	T	V	L	S	I	T	D	G	Q	I	V	L	S	R	D	L	F	N	R	G	I	Y	P
Posterior probability	0.492	0.407	0.91	0.404	0.45	0.298	0.575	0.864	0.55	0.919	0.989	0.432	0.582	0.47	0.992	0.676	0.99	0.716	0.264	0.498	0.238	0.618	0.798	0.448	0.89	0.992	0.583	0.842	0.696	0.908	0.357	0.913	0.848	0.964	0.776	0.333	0.599	0.999	0.559	0.962	1
Sequence	A	L	P	V	I	S	V	P	G	D	D	I	T	E	P	I	P	D	T	T	L	A	I	T	D	G	Q	I	V	L	S	R	D	L	F	H	R	G	I	Y	P
Posterior probability	0.507	0.377	0.904	0.422	0.458	0.321	0.585	0.912	0.554	0.923	0.99	0.389	0.615	0.561	0.994	0.536	0.991	0.693	0.275	0.455	0.27	0.344	0.814	0.432	0.883	0.989	0.582	0.816	0.73	0.977	0.625	0.905	0.861	0.966	0.581	0.249	0.498	0.999	0.539	0.966	1
Sequence	Q	A	V	S	V	P	G	D	D	L	T	E	P	V	P	E	T	T	L	A	I	L	D	A	H	I	V	L	S	R	D	L	A	E	R	G	I	Y	P	A	I
Posterior probability	0.314	0.792	0.578	0.3	0.777	0.995	0.736	0.918	0.99	0.736	0.646	0.546	1	0.654	0.949	0.241	0.464	0.679	0.469	0.493	0.598	0.633	0.867	0.73	0.322	0.681	0.767	0.971	0.486	0.887	0.648	0.89	0.907	0.279	0.76	0.982	0.584	0.929	1	0.977	0.559
Sequence	Q	A	V	S	V	P	G	D	D	L	T	E	P	V	P	D	T	T	L	A	I	L	D	A	H	I	V	L	S	R	D	L	A	E	R	G	I	Y	P	A	I
Posterior probability	0.285	0.776	0.573	0.344	0.767	0.996	0.72	0.919	0.991	0.609	0.656	0.651	1	0.709	0.957	0.246	0.444	0.719	0.483	0.542	0.646	0.586	0.866	0.703	0.34	0.671	0.797	0.98	0.636	0.882	0.699	0.899	0.913	0.305	0.653	0.984	0.542	0.934	1	0.979	0.595
Sequence	G	S	L	T	A	L	P	I	I	E	T	Q	A	G	D	V	S	A	Y	I	P	T	N	V	I	S	I	T	D	G	Q	I	Y	L	E	S	D	L	F	Y	S
Posterior probability	1	0.999	0.997	1	0.999	0.998	1	0.995	0.977	0.998	0.999	0.999	0.999	1	1	0.995	0.998	0.998	0.999	0.991	1	0.998	0.999	0.982	0.995	0.998	0.997	0.999	1	1	0.999	0.997	0.629	0.999	0.998	0.794	0.999	0.999	1	0.985	0.757
Sequence	S	L	T	A	L	P	I	I	E	T	Q	A	G	D	I	S	A	Y	I	P	T	N	V	I	S	I	T	D	G	Q	I	F	L	E	T	D	L	F	Y	K	G
Posterior probability	0.998	0.995	0.999	0.998	0.996	1	0.991	0.99	0.997	0.998	0.592	0.997	0.999	0.999	0.55	0.995	0.154	0.998	0.993	1	0.996	0.998	0.992	0.99	0.996	0.994	0.998	0.999	1	0.999	0.994	0.97	0.999	0.996	0.931	0.999	0.999	0.998	0.998	0.774	1
Sequence	R	G	S	I	T	S	V	Q	A	I	Y	V	P	A	D	D	L	T	D	P	A	P	A	T	T	F	A	H	L	D	A	T	I	V	L	S	R	E	L	A	E
Posterior probability	0.556	1	0.998	0.99	0.999	0.988	0.986	0.995	0.997	0.63	0.996	0.991	1	0.99	0.999	0.999	0.946	0.994	0.992	1	0.99	1	0.991	0.993	0.737	0.996	0.992	0.997	0.992	0.998	0.996	0.992	0.798	0.873	0.998	0.815	0.998	0.653	0.613	0.998	0.99
Sequence	K	G	S	I	T	S	V	Q	A	I	Y	V	P	A	D	D	L	T	D	P	A	P	A	T	T	F	A	H	L	D	A	T	I	V	L	S	R	E	L	A	E
Posterior probability	0.578	1	0.998	0.992	0.999	0.99	0.987	0.996	0.997	0.63	0.996	0.992	1	0.992	0.999	0.999	0.946	0.995	0.992	1	0.991	1	0.992	0.994	0.778	0.996	0.994	0.997	0.993	0.998	0.996	0.994	0.767	0.899	0.999	0.85	0.998	0.613	0.572	0.998	0.992
Sequence	R	T	G	R	P	L	M	E	R	T	V	L	I	A	N	T	S	N	M	P	V	A	A	R	E	A	S	I	Y	T	G	I	T	L	A	E	Y	F	R	D	Q
Posterior probability	0.604	0.973	0.999	0.641	0.999	0.99	0.995	0.887	0.997	0.998	0.985	0.996	0.981	0.994	0.996	0.998	0.992	0.998	0.997	1	0.988	0.996	0.998	0.999	0.993	0.993	0.989	0.89	0.997	0.778	0.925	0.683	0.999	0.519	0.999	0.999	0.996	0.988	0.999	0.999	0.776
Sequence	T	G	R	P	L	M	E	R	T	V	L	I	A	N	T	S	N	M	P	V	A	A	R	E	A	S	I	Y	T	G	I	T	M	A	E	Y	F	R	D	Q	G
Posterior probability	0.974	0.999	0.955	0.999	0.989	0.995	0.97	0.997	0.998	0.958	0.996	0.98	0.994	0.996	0.998	0.991	0.998	0.997	1	0.988	0.996	0.998	0.999	0.993	0.993	0.988	0.894	0.997	0.85	0.538	0.683	0.998	0.696	0.999	0.999	0.996	0.982	0.999	0.999	0.791	1
Sequence	L	Y	E	R	A	G	K	L	N	G	R	K	G	S	V	T	Q	V	P	I	L	S	M	P	S	D	D	I	T	H	P	I	P	D	L	T	G	Y	I	T	E
Posterior probability	0.953	1	1	1	1	1	1	1	1	1	0.998	1	1	1	1	1	1	1	1	1	1	1	1	1	1	1	1	1	1	1	1	1	1	1	1	1	1	1	1	1	1
Sequence	A	S	I	Y	E	R	A	G	R	I	K	G	K	K	G	S	I	T	Q	M	P	I	L	S	M	P	S	D	D	I	T	H	P	I	P	D	L	T	G	Y	I
Posterior probability	0.982	0.696	0.886	0.999	1	1	1	1	0.998	0.954	0.991	1	0.944	0.996	1	0.999	0.524	1	0.999	0.574	1	0.995	0.997	0.833	0.999	1	0.933	1	1	0.996	0.999	0.999	1	0.996	1	0.999	0.997	0.999	0.999	0.999	0.997

Sequence	D	V	L	Q	S	V	S	R	L	M	S	P	S	I	V	S	E	E	H	Q	E	V	A	Q	K	V	R	E	L	L	A	R	Y	R	E	L	Q	D	L	V	N
Posterior probability	0.618	0.788	0.961	0.57	0.974	0.626	0.974	0.987	0.486	0.85	0.266	0.379	0.384	0.74	0.537	0.505	0.665	0.782	0.97	0.534	0.544	0.627	0.869	0.463	0.755	0.657	0.909	0.48	0.523	0.956	0.696	0.579	0.965	0.65	0.909	0.608	0.778	0.803	0.798	0.474	0.314

Sequence	D	V	L	S	S	V	S	R	L	M	D	P	R	I	V	S	P	E	H	R	E	V	A	R	K	V	R	A	L	L	A	R	Y	Q	E	L	E	D	L	V	Q
Posterior probability	0.567	0.849	0.963	0.282	0.976	0.616	0.975	0.987	0.77	0.829	0.265	0.425	0.319	0.679	0.689	0.256	0.691	0.704	0.975	0.297	0.797	0.725	0.93	0.626	0.518	0.614	0.883	0.531	0.539	0.958	0.685	0.792	0.963	0.575	0.761	0.838	0.524	0.82	0.77	0.489	0.564

Sequence	A	I	N	V	L	M	S	V	S	R	L	M	S	P	A	I	V	S	E	E	H	Q	E	V	A	Q	K	V	R	E	A	L	A	R	Y	R	E	L	Q	A	L
Posterior probability	0.908	0.779	0.596	0.723	0.897	0.323	0.952	0.579	0.952	0.982	0.478	0.831	0.212	0.27	0.372	0.643	0.502	0.344	0.529	0.665	0.947	0.405	0.547	0.683	0.8	0.258	0.714	0.602	0.884	0.427	0.282	0.881	0.681	0.6	0.941	0.656	0.931	0.59	0.77	0.395	0.786

Sequence	A	I	N	V	L	M	S	V	S	R	L	M	D	P	A	I	V	E	P	E	H	P	E	V	A	R	K	V	R	A	L	L	A	R	Y	Q	E	L	E	D	L
Posterior probability	0.916	0.756	0.611	0.848	0.912	0.307	0.958	0.575	0.958	0.983	0.738	0.821	0.197	0.334	0.24	0.615	0.628	0.267	0.567	0.615	0.957	0.283	0.758	0.755	0.88	0.391	0.497	0.367	0.865	0.536	0.262	0.892	0.682	0.832	0.943	0.548	0.827	0.855	0.518	0.442	0.773

Sequence	D	V	L	Q	S	V	S	R	L	M	D	P	S	I	V	S	E	E	H	Q	E	V	A	R	K	V	R	E	L	L	Q	R	Y	K	E	L	Q	D	I	I	Q
Posterior probability	0.702	0.275	0.971	0.58	0.961	0.253	0.959	0.943	0.423	0.581	0.596	0.636	0.368	0.636	0.593	0.408	0.635	0.754	0.946	0.233	0.817	0.591	0.862	0.361	0.467	0.69	0.679	0.405	0.47	0.97	0.842	0.667	0.858	0.456	0.723	0.874	0.922	0.812	0.644	0.546	0.341

Sequence	D	V	L	T	S	V	S	R	L	M	D	P	R	I	V	S	P	E	H	P	E	V	A	R	Q	V	R	A	L	L	Q	R	Y	Q	E	L	Q	D	I	I	Q
Posterior probability	0.639	0.337	0.973	0.327	0.966	0.296	0.965	0.945	0.615	0.59	0.654	0.633	0.401	0.54	0.688	0.272	0.641	0.693	0.951	0.311	0.883	0.668	0.904	0.635	0.47	0.703	0.673	0.632	0.497	0.972	0.828	0.787	0.883	0.565	0.646	0.933	0.811	0.827	0.622	0.514	0.584

Sequence	G	I	R	P	A	I	N	V	G	L	S	V	S	R	V	G	G	A	A	Q	I	K	A	M	K	Q	V	A	G	K	L	R	L	D	L	A	Q	Y	R	E	L
Posterior probability	1	0.991	0.999	1	0.999	0.997	0.999	0.997	0.999	0.979	0.999	0.996	0.999	1	0.995	0.999	1	0.597	0.999	0.999	0.995	0.999	0.998	0.713	0.998	0.999	0.99	0.858	1	0.846	0.997	1	0.994	0.998	0.996	0.999	0.999	0.997	0.999	1	0.998

Sequence	I	R	P	A	I	N	V	G	L	S	V	S	R	V	G	G	A	A	Q	I	K	A	E	K	Q	V	A	G	K	L	R	L	E	L	A	R	Y	R	E	L	E
Posterior probability	0.518	0.998	1	0.999	0.994	0.998	0.995	0.999	0.995	0.998	0.993	0.998	0.999	0.989	0.999	0.999	0.993	0.997	0.998	0.99	0.168	0.099	0.211	0.214	0.998	0.994	0.983	0.999	0.742	0.996	0.999	0.995	0.577	0.998	0.998	0.752	1	0.997	0.999	0.998	0.956

Sequence	K	G	I	Y	P	A	V	D	P	L	Q	S	T	S	R	I	M	D	P	R	I	V	S	E	E	H	Y	E	V	A	R	R	V	R	E	I	L	Q	R	Y	K
Posterior probability	0.53	1	0.922	0.999	1	0.999	0.978	0.996	0.999	0.998	0.571	0.998	0.571	0.998	0.973	0.596	0.505	0.996	0.999	0.861	0.698	0.741	0.477	0.605	0.995	1	0.995	0.877	0.984	0.997	0.601	0.554	0.847	0.59	0.426	0.498	0.998	0.999	0.994	0.86	0.695

Sequence	K	G	I	Y	P	A	V	D	P	L	D	S	T	S	R	I	L	D	P	R	V	V	G	E	E	H	Y	E	V	A	R	Q	V	R	A	V	L	Q	R	Y	K
Posterior probability	0.59	1	0.935	0.999	1	0.999	0.981	0.996	0.999	0.998	0.464	0.998	0.643	0.998	0.962	0.526	0.508	0.997	0.999	0.954	0.596	0.831	0.489	0.359	0.994	1	0.996	0.882	0.988	0.998	0.827	0.727	0.88	0.558	0.579	0.58	0.998	0.999	0.997	0.887	0.512

Sequence	G	Y	D	V	A	L	M	A	D	S	T	S	R	W	A	E	A	L	R	E	I	S	G	R	L	G	E	M	P	G	E	E	G	Y	P	A	Y	L	A	S	R
Posterior probability	1	0.884	0.998	0.99	0.993	0.982	0.996	0.993	0.999	0.992	0.995	0.991	0.999	0.999	0.998	0.995	0.999	0.938	0.998	0.999	0.985	0.997	0.998	0.995	0.997	0.888	0.711	0.823	1	0.654	0.997	0.996	0.999	0.996	1	0.994	0.997	0.996	0.878	0.99	0.992

Sequence	Y	D	V	A	L	M	A	D	S	T	S	R	W	A	E	A	L	R	E	I	S	G	R	L	G	E	M	P	G	E	E	G	Y	P	A	Y	L	A	S	R	L
Posterior probability	0.889	0.999	0.99	0.993	0.984	0.996	0.992	0.999	0.991	0.995	0.99	0.998	0.999	0.998	0.995	0.999	0.928	0.998	0.999	0.984	0.997	0.997	0.995	0.998	0.885	0.73	0.823	1	0.761	0.996	0.995	1	0.996	1	0.993	0.997	0.995	0.881	0.989	0.991	0.983

Sequence	G	Q	I	V	V	G	R	D	L	F	R	Q	G	V	Y	P	P	I	N	I	L	M	S	L	S	R	L	M	K	D	G	I	G	E	G	S	T	R	A	D	H
Posterior probability	1	1	0.998	1	0.7	1	1	1	1	1	1	1	1	1	1	1	1	0.997	1	1	1	1	1	1	1	1	1	1	1	1	1	1	1	1	1	1	1	1	1	1	1

Sequence	T	E	G	Q	I	V	L	S	R	D	L	Y	R	K	G	I	Y	P	P	I	N	V	L	M	S	L	S	R	L	M	K	D	G	I	G	E	G	K	T	R	A
Posterior probability	0.999	0.998	1	0.999	0.997	0.996	0.999	0.802	0.999	0.998	0.999	0.51	0.995	0.992	1	0.666	1	1	1	0.696	0.999	0.967	0.999	0.995	0.999	0.997	0.999	1	0.999	1	0.976	0.962	1	0.956	0.965	0.751	0.996	0.791	0.999	0.999	0.803

Sequence	I	A	G	Y	E	K	L	N	E	E	D	R	K	A	I	E	R	A	Q	K	I	Q	E	F	L	R	Q	P	I	H	E	S	S	P	I	E	E	T	V	K	M
Posterior probability	0.779	0.5	0.823	0.202	0.369	0.367	0.884	0.559	0.854	0.435	0.375	0.401	0.325	0.418	0.477	0.87	0.507	0.666	0.428	0.761	0.717	0.33	0.456	0.991	0.962	0.327	0.996	0.733	0.372	0.498	0.945	0.268	0.29	0.897	0.526	0.979	0.73	0.961	0.573	0.208	0.269
Sequence	I	A	G	Y	A	R	L	N	E	E	D	R	K	A	L	E	R	A	R	K	I	Q	E	F	L	R	Q	P	I	H	E	A	S	P	I	E	E	T	I	R	A
Posterior probability	0.709	0.462	0.872	0.151	0.383	0.178	0.907	0.472	0.819	0.661	0.423	0.461	0.539	0.356	0.46	0.928	0.61	0.602	0.56	0.69	0.784	0.455	0.353	0.992	0.975	0.455	0.997	0.598	0.252	0.728	0.948	0.256	0.202	0.981	0.405	0.965	0.807	0.962	0.504	0.273	0.206
Sequence	A	Q	I	A	G	N	A	K	G	L	E	A	D	R	K	A	L	E	R	G	Q	K	I	Q	E	F	L	K	Q	P	I	H	E	S	S	P	I	E	E	T	V
Posterior probability	0.379	0.288	0.702	0.53	0.825	0.153	0.355	0.265	0.728	0.914	0.857	0.321	0.406	0.478	0.432	0.282	0.59	0.909	0.557	0.741	0.445	0.636	0.483	0.273	0.52	0.96	0.96	0.286	0.99	0.742	0.203	0.461	0.896	0.287	0.192	0.895	0.546	0.976	0.755	0.905	0.527
Sequence	V	Q	I	A	G	K	A	G	L	N	E	E	D	R	K	A	L	E	R	G	R	K	I	Q	E	F	L	K	Q	P	P	H	E	S	A	P	I	E	E	T	I
Posterior probability	0.364	0.578	0.669	0.481	0.871	0.152	0.396	0.245	0.926	0.42	0.821	0.411	0.449	0.526	0.545	0.257	0.622	0.926	0.64	0.751	0.494	0.58	0.558	0.412	0.383	0.969	0.97	0.584	0.991	0.603	0.215	0.665	0.909	0.237	0.155	0.974	0.42	0.943	0.787	0.917	0.497
Sequence	I	V	G	M	E	E	L	S	E	E	D	R	T	I	L	E	R	A	R	K	I	Q	E	F	L	S	Q	P	F	H	E	A	E	Q	F	T	P	I	E	E	T
Posterior probability	0.721	0.393	0.983	0.18	0.647	0.458	0.951	0.627	0.96	0.514	0.589	0.426	0.199	0.396	0.362	0.754	0.651	0.9	0.801	0.793	0.755	0.501	0.322	0.991	0.941	0.321	0.992	0.771	0.8	0.9	0.881	0.569	0.539	0.192	0.873	0.414	0.992	0.621	0.925	0.56	0.953
Sequence	I	V	G	M	E	E	L	S	E	E	D	R	L	V	L	E	R	A	R	K	I	Q	E	F	L	K	Q	P	F	H	E	A	E	Q	F	T	P	I	E	E	T
Posterior probability	0.698	0.435	0.985	0.156	0.621	0.34	0.955	0.645	0.951	0.67	0.575	0.474	0.415	0.378	0.423	0.829	0.703	0.869	0.889	0.741	0.854	0.561	0.362	0.992	0.956	0.24	0.993	0.65	0.806	0.959	0.9	0.566	0.559	0.212	0.86	0.395	0.998	0.479	0.951	0.635	0.956
Sequence	Q	A	F	A	Q	F	A	S	D	L	D	E	A	T	R	A	Q	L	E	R	G	Q	R	M	T	E	I	L	K	Q	P	Q	Y	S	P	M	P	V	E	K	Q
Posterior probability	0.977	0.999	0.999	0.993	0.999	0.999	0.996	0.998	0.998	0.999	0.999	0.562	0.999	0.747	0.98	0.485	0.999	0.998	0.971	0.999	1	0.999	0.993	0.99	0.907	0.999	0.88	0.999	0.998	1	0.997	0.792	0.999	0.988	1	0.86	0.997	0.969	0.999	0.971	0.999
Sequence	A	F	A	Q	F	A	S	D	L	D	E	A	T	R	A	Q	L	E	R	G	R	R	M	M	E	L	L	K	Q	P	P	Y	S	P	I	P	V	E	K	Q	I
Posterior probability	0.996	0.998	0.68	0.999	0.998	0.998	0.995	0.997	0.998	0.997	0.609	0.997	0.964	0.984	0.958	0.998	0.997	0.995	0.998	1	0.559	0.997	0.998	0.525	0.998	0.995	0.999	0.997	1	0.959	0.993	0.998	0.995	1	0.402	1	0.986	0.999	0.997	0.998	0.606
Sequence	D	L	Q	D	I	I	A	I	L	G	M	E	E	L	S	E	E	D	K	L	I	V	Q	R	A	R	K	I	Q	R	F	L	S	Q	P	F	H	V	A	E	H
Posterior probability	0.99	0.996	0.999	0.997	0.988	0.982	0.991	0.989	0.642	1	0.996	0.895	0.995	0.998	0.993	0.998	0.995	0.994	0.733	0.408	0.606	0.976	0.519	0.994	0.998	0.997	0.948	0.99	0.996	0.989	0.999	0.87	0.77	1	1	0.998	0.893	0.989	0.994	0.993	0.406
Sequence	D	L	Q	D	I	I	A	I	L	G	M	E	E	L	S	E	E	D	K	L	I	V	Q	R	A	R	K	I	Q	R	F	L	S	Q	P	F	H	V	A	E	Q
Posterior probability	0.992	0.998	0.998	0.998	0.989	0.984	0.993	0.99	0.682	1	0.997	0.97	0.995	0.998	0.995	0.999	0.997	0.995	0.77	0.606	0.552	0.979	0.572	0.996	0.998	0.998	0.962	0.993	0.997	0.991	0.999	0.89	0.778	1	0.999	0.999	0.998	0.99	0.995	0.994	0.465
Sequence	L	A	E	F	Y	E	R	A	G	R	V	K	T	L	G	S	D	E	R	V	G	S	I	T	V	V	G	A	V	S	P	P	G	G	D	F	S	E	P	V	T
Posterior probability	0.981	0.996	0.795	0.99	0.996	0.999	0.999	0.904	1	0.994	0.986	0.977	0.584	0.953	0.999	0.991	0.69	0.567	0.995	0.439	0.999	0.997	0.736	0.999	0.805	0.895	0.998	0.997	0.985	0.992	0.999	1	0.999	0.998	0.999	0.997	0.99	0.995	1	0.988	0.992
Sequence	A	E	F	Y	E	R	A	G	R	V	K	T	L	G	S	D	E	R	V	G	S	I	T	V	V	G	A	V	S	P	P	G	G	D	F	S	E	P	V	T	Q
Posterior probability	0.997	0.804	0.991	0.996	0.999	0.999	0.908	1	0.995	0.986	0.991	0.683	0.971	0.998	0.991	0.798	0.617	0.994	0.441	0.999	0.997	0.709	0.999	0.683	0.879	0.998	0.997	0.984	0.991	0.999	1	0.999	0.998	0.999	0.996	0.989	0.995	1	0.988	0.992	0.995
Sequence	S	E	V	S	N	Q	V	Y	D	A	Y	S	R	A	Q	E	V	R	A	L	A	G	I	V	G	K	A	G	L	T	E	I	D	L	K	Y	L	E	V	G	D
Posterior probability	0.943	1	0.997	1	1	1	1	1	1	1	1	1	1	1	1	1	1	1	1	1	1	1	1	1	1	1	1	1	1	1	1	1	1	1	1	1	0.92	0.948	1	1	1
Sequence	D	H	P	E	V	S	N	Q	L	Y	A	A	Y	S	R	A	Q	E	L	R	S	L	A	E	I	V	G	K	A	G	L	S	E	T	D	L	K	Y	L	E	F
Posterior probability	0.999	1	0.818	0.579	0.968	0.954	0.999	0.999	0.701	0.999	0.745	0.983	0.998	0.866	0.999	0.998	0.809	0.984	0.801	0.998	0.671	0.999	0.999	0.951	0.992	0.997	1	0.924	0.653	0.984	0.999	0.576	0.977	0.986	0.999	0.913	0.999	0.999	0.977	0.739	0.43

Sequence	L	K	E	I	L	N	G	Y	L	D	D	I	P	E	E	E	F	K	E	F	K	D	G	Y	I	E	Q	V	Q	V	E										
Posterior probability	0.783	0.371	0.507	0.774	0.39	0.364	0.859	0.317	0.682	0.717	0.755	0.435	0.997	0.355	0.331	0.524	0.332	0.212	0.685	0.291	0.352	0.25	0.792	0.312	0.531	0.842	0.33	0.189	0.153	0.207	0.515										
Sequence	L	M	E	I	L	N	G	Y	L	D	D	P	E	E	A	F	T	K	R	E	T	I	E	K	V	L	E	K	A	K	E	M	L								
Posterior probability	0.63	0.301	0.867	0.691	0.893	0.461	0.663	0.331	0.676	0.718	0.761	0.999	0.84	0.462	0.437	0.467	0.371	0.63	0.225	0.55	0.282	0.605	0.569	0.449	0.149	0.208	0.535	0.654	0.192	0.365	0.38	0.169	0.499								
Sequence	T	L	L	W	E	I	V	N	G	Y	L	D	D	P	K	E	E	I	K	E	F	K	D	G	Y	I	E	Q	V	Y	E	K	E	P	E						
Posterior probability	0.315	0.413	0.655	0.309	0.406	0.629	0.347	0.405	0.855	0.34	0.706	0.734	0.764	0.997	0.328	0.311	0.557	0.41	0.267	0.683	0.329	0.413	0.447	0.786	0.467	0.466	0.845	0.397	0.172	0.17	0.39	0.463	0.551	0.231	0.241						
Sequence	R	V	L	W	E	I	L	N	G	Y	L	D	D	I	P	E	E	L	T	K	F	K	K	E	T	I	E	K	M	Y	E	K	H	K	E	V	L				
Posterior probability	0.18	0.208	0.513	0.289	0.853	0.524	0.848	0.501	0.663	0.395	0.697	0.731	0.767	0.467	1	0.831	0.484	0.408	0.498	0.76	0.315	0.475	0.246	0.683	0.199	0.573	0.513	0.442	0.184	0.211	0.436	0.679	0.185	0.361	0.386	0.162	0.5				
Sequence	I	R	M	L	K	E	I	L	N	G	Y	L	D	D	I	P	E	E	E	F	E	K	V	G	T	I	E	E	A	V	E	K	E	K	K	M	L	K	E		
Posterior probability	0.424	0.719	0.367	0.704	0.769	0.516	0.848	0.605	0.309	0.877	0.346	0.65	0.714	0.77	0.404	1	0.582	0.378	0.478	0.797	0.2	0.247	0.27	0.818	0.552	0.764	0.873	0.434	0.325	0.366	0.861	0.464	0.593	0.286	0.33	0.322	0.522	0.424	0.407		
Sequence	I	R	M	L	K	E	I	L	N	G	Y	L	D	D	I	P	E	E	A	F	T	K	R	D	T	I	E	K	V	L	E	K	A	K	E	M	L	K	E		
Posterior probability	0.528	0.704	0.265	0.595	0.707	0.815	0.813	0.915	0.427	0.713	0.332	0.642	0.711	0.771	0.422	1	0.893	0.418	0.494	0.843	0.179	0.402	0.231	0.418	0.557	0.759	0.715	0.488	0.243	0.356	0.792	0.632	0.365	0.387	0.367	0.239	0.515	0.403	0.558		
Sequence	V	V	I	I	Y	A	G	T	N	G	Y	L	D	D	I	P	V	E	K	V	K	E	F	E	D	G	F	L	E	Y	I	E	S	K	H	P	D	I	L	E	E
Posterior probability	0.977	0.945	0.966	0.996	0.978	0.998	0.999	0.994	0.838	1	0.56	0.999	0.999	0.998	0.968	0.908	0.992	0.55	0.974	0.571	0.88	0.967	0.999	0.999	0.554	0.945	0.898	0.854	0.525	0.832	0.603	0.994	0.739	0.992	0.989	1	0.898	0.976	0.984	0.994	0.98
Sequence	V	V	I	F	A	A	I	N	G	Y	L	D	D	I	P	V	E	A	V	T	K	F	E	R	E	L	Y	E	F	M	E	A	K	H	S	E	V	L	K	E	I
Posterior probability	0.993	0.331	0.481	0.951	0.997	0.795	0.69	0.995	1	0.982	0.998	0.999	0.999	0.988	1	0.994	0.992	0.752	0.978	0.635	0.986	0.999	0.997	0.457	0.995	0.975	0.93	0.393	0.914	0.964	0.112	0.929	0.998	0.637	0.552	0.579	0.559	0.996	0.463	0.997	0.987
Sequence	F	T	G	R	P	G	K	Y	V	P	I	E	D	T	I	R	G	F	K	E	I	L	D	G	K	L	D	D	V	P	E	Q	A	F	Y	M	V	G	T	I	E
Posterior probability	0.998	0.993	0.999	0.494	0.686	0.999	0.515	0.997	0.985	0.991	0.975	0.998	0.841	0.999	0.677	0.99	0.996	0.995	0.995	0.703	0.991	0.744	0.637	0.999	0.697	0.77	0.996	0.997	0.528	1	0.995	0.994	0.993	0.997	0.987	0.997	0.586	0.999	0.993	0.989	0.886
Sequence	F	T	G	R	P	G	K	Y	V	P	I	E	D	T	I	R	G	F	K	E	I	L	D	G	K	L	D	D	V	P	E	Q	A	F	Y	M	V	G	T	I	E
Posterior probability	0.998	0.994	0.999	0.879	0.723	0.999	0.572	0.998	0.987	0.995	0.829	0.999	0.872	0.999	0.752	0.99	0.997	0.996	0.995	0.887	0.991	0.947	0.779	0.999	0.66	0.756	0.997	0.997	0.417	1	0.998	0.994	0.996	0.998	0.989	0.997	0.497	0.998	0.99	0.991	0.754
Sequence	Q	N	T	L	R	I	V	K	V	F	W	A	L	D	K	D	L	A	S	R	R	F	F	P	A	I	N	T	L	Q	S	Y	S	L	Y	F	D	P	S	L	E
Posterior probability	0.995	0.506	0.997	0.994	0.995	0.986	0.978	0.993	0.988	0.994	1	0.994	0.998	0.977	0.948	0.99	0.997	0.998	0.301	0.996	0.995	0.499	0.991	1	0.998	0.985	0.661	0.246	0.998	0.471	0.997	0.996	0.985	0.993	0.994	0.464	0.535	0.45	0.599	0.641	0.994
Sequence	N	T	L	R	I	V	K	V	F	W	A	L	D	K	D	L	A	S	R	R	F	F	P	A	I	N	T	L	T	S	Y	S	L	Y	F	D	S	L	E	E	W
Posterior probability	0.493	0.997	0.984	0.994	0.985	0.975	0.99	0.985	0.993	0.999	0.993	0.998	0.974	0.984	0.993	0.997	0.998	0.296	0.995	0.995	0.464	0.989	1	0.997	0.985	0.72	0.259	0.996	0.769	0.997	0.995	0.987	0.991	0.993	0.435	0.662	0.491	0.575	0.995	0.654	0.998
Sequence	T	F	E	K	E	F	L	T	Q	A	T	D	E	N	R	T	I	E	E	T	L	T	L	M	W	K	I	V	S	K	L	P	K	N	E	I	T	K	I	K	D
Posterior probability	0.963	1	1	1	1	1	1	0.913	1	1	1	1	1	1	1	1	1	1	1	1	1	1	0.998	0.913	1	1	1	1	1	1	1	1	1	1	1	1	1	1	1	1	1
Sequence	G	D	M	F	E	Q	R	F	L	K	Q	A	S	N	E	N	R	T	I	E	E	T	L	E	I	A	W	E	V	L	S	T	L	P	E	S	E	L	T	K	I
Posterior probability	0.999	0.997	0.461	0.999	0.998	0.719	0.761	1	0.999	0.89	1	0.505	0.425	0.576	0.999	0.999	0.998	0.84	0.59	0.645	0.884	0.986	0.997	0.4	0.892	0.973	1	0.97	0.687	0.992	0.998	0.749	0.997	1	0.996	0.666	0.999	0.998	0.999	0.991	0.988

Sequence
Posterior probability

Sequence
Posterior probability

Sequence
Posterior probability

Sequence
Posterior probability

Sequence
Posterior probability

Sequence
Posterior probability

Sequence	I	R	E	K	K	A	L	D	D	E	L	E	E	K	L	K	K	A	I	K	E	F	K	A	T	F	K
Posterior probability	0.994	0.973	0.527	0.999	0.999	0.534	0.998	0.997	0.709	0.604	0.554	0.997	0.996	0.991	0.983	0.887	0.998	0.99	0.972	0.678	0.999	0.999	0.998	0.809	0.988	0.801	0.59

Sequence	R	E	K	K	Q	L	D	D	E	L	T	Q	K	L	H	K	A	I	K	E	F	K	A	T	F	T	A
Posterior probability	0.537	0.981	0.998	0.998	0.475	0.996	0.997	0.992	0.964	0.993	0.638	0.723	0.997	0.964	0.561	0.501	0.997	0.99	0.514	0.998	0.998	0.997	0.967	0.996	0.978	0.929	0.756

Sequence	E	A	V	E	K	A	K	K	M	K	K	E
Posterior probability	0.994	0.631	0.609	0.997	0.63	0.993	0.667	0.774	0.704	0.505	0.961	0.403

Sequence	E	V	L	E	K	A	K	K	M	R	K	E
Posterior probability	0.994	0.555	0.572	0.997	0.775	0.994	0.691	0.808	0.598	0.495	0.761	0.653

Sequence	W	H	S	E	N	V	S	D	D	W	P	E	L	R	K	E	A	M	A	L	L	Q	K	D	Q	E	L	Q	E	I	V	Q	L	V	G	P	D	A	L	P	E
Posterior probability	0.998	0.41	0.273	0.752	0.997	0.688	0.592	0.321	0.957	0.996	0.787	0.977	0.99	0.389	0.618	0.994	0.993	0.911	0.991	0.986	0.998	0.999	0.981	0.825	0.526	0.996	0.997	0.912	0.924	0.988	0.983	0.987	0.99	0.985	1	0.999	0.994	0.994	0.997	0.999	0.996

Sequence	H	Q	E	N	V	S	P	D	W	P	E	L	R	K	E	A	M	A	L	L	Q	K	D	Q	E	L	Q	E	I	V	Q	L	V	G	P	D	A	L	P	E	S
Posterior probability	0.403	0.328	0.881	0.996	0.699	0.616	0.693	0.962	0.996	0.799	0.984	0.988	0.383	0.555	0.977	0.992	0.911	0.967	0.987	0.998	0.999	0.964	0.796	0.661	0.995	0.997	0.907	0.926	0.987	0.982	0.989	0.989	0.985	1	0.999	0.993	0.994	0.997	0.999	0.978	0.986

Sequence	K	Y	V	E	Q	Y	Y	K	E	E
Posterior probability	1	1	0.99	0.941	1	1	1	0.999	1	0.996

Sequence	K	E	E	H	I	K	K	Y	Y	K	K	S	K	S
Posterior probability	0.999	0.986	0.871	0.667	0.969	0.881	0.739	0.98	0.979	0.713	0.477	0.438	0.516	0.579

Sequence																																									
Posterior probability																																									
Sequence																																									
Posterior probability																																									
Sequence																																									
Posterior probability																																									
Sequence																																									
Posterior probability																																									
Sequence																																									
Posterior probability																																									
Sequence																																									
Posterior probability																																									
Sequence																																									
Posterior probability																																									
Sequence																																									
Posterior probability																																									
Sequence																																									
Posterior probability																																									
Sequence																																									
Posterior probability																																									
Sequence	S	E	R	V	I	L	E	T	A	R	M	I	K	E	D	F	L	Q	Q	N	A	F	H	E	V	D	T	F	C	P	L	D	K	T	Y	R	M	L	K	T	I
Posterior probability	0.848	0.993	0.948	0.506	0.913	0.992	0.997	0.583	0.998	0.997	0.997	0.574	0.76	0.995	0.996	0.999	0.997	0.569	0.999	0.711	0.994	0.863	1	0.997	0.719	0.994	0.972	0.826	0.99	1	0.372	0.589	0.994	0.733	0.994	0.78	0.997	0.995	0.774	0.465	0.989
Sequence	E	R	L	I	L	E	A	A	R	M	I	K	E	D	F	L	Q	Q	N	A	F	H	E	V	D	T	F	C	P	L	E	K	T	Y	R	M	L	K	V	I	L
Posterior probability	0.992	0.969	0.529	0.909	0.992	0.998	0.48	0.998	0.998	0.997	0.978	0.967	0.995	0.994	0.999	0.997	0.689	0.999	0.712	0.994	0.869	1	0.998	0.74	0.993	0.974	0.799	0.989	0.999	0.528	0.726	0.994	0.736	0.994	0.696	0.997	0.994	0.761	0.399	0.988	0.856
Sequence																																									
Posterior probability																																									
Sequence																																									
Posterior probability																																									

Sequence																																									
Posterior probability																																									
Sequence																																									
Posterior probability																																									
Sequence																																									
Posterior probability																																									
Sequence																																									
Posterior probability																																									
Sequence																																									
Posterior probability																																									
Sequence																																									
Posterior probability																																									
Sequence																																									
Posterior probability																																									
Sequence																																									
Posterior probability																																									
Sequence																																									
Posterior probability																																									
Sequence																																									
Posterior probability																																									
Sequence	L	K	F	Y	E	K	A	A	E	A	V	E	R	G	V	Q	V	E	K	I	L	E	L	S	V	L	E	N	I	A	R	M	K	Y	I	P	E	E	E	Y	E
Posterior probability	0.72	0.439	0.996	0.56	0.543	0.984	0.761	0.421	0.965	0.99	0.954	0.41	0.56	0.999	0.965	0.745	0.963	0.401	0.527	0.983	0.896	0.949	0.932	0.836	0.957	0.461	0.628	0.593	0.948	0.995	0.996	0.995	0.993	0.744	0.734	1	0.416	0.628	0.678	0.736	0.956
Sequence	N	F	Y	E	K	A	Q	E	A	V	K	K	G	V	Q	V	A	E	I	L	E	L	P	V	I	E	N	I	A	R	M	K	Y	I	P	E	D	E	F	E	E
Posterior probability	0.494	0.996	0.615	0.447	0.944	0.844	0.761	0.982	0.985	0.739	0.515	0.482	0.999	0.967	0.709	0.887	0.411	0.494	0.982	0.685	0.987	0.746	0.518	0.954	0.417	0.471	0.508	0.956	0.994	0.995	0.995	0.993	0.781	0.681	1	0.686	0.617	0.586	0.531	0.947	0.99
Sequence																																									
Posterior probability																																									
Sequence																																									
Posterior probability																																									

Sequence																						
Posterior probability																						
Sequence																						
Posterior probability																						
Sequence																						
Posterior probability																						
Sequence																						
Posterior probability																						
Sequence																						
Posterior probability																						
Sequence																						
Posterior probability																						
Sequence																						
Posterior probability																						
Sequence																						
Posterior probability																						
Sequence																						
Posterior probability																						
Sequence																						
Posterior probability																						
Sequence	E	T	L	N	T	I	E	K	K	I	E	G	E	F	E	S	L	F	Q	E	A	S
Posterior probability	0.994	0.533	0.6	0.521	0.372	0.976	0.741	0.992	0.498	0.715	0.868	0.534	0.982	0.951	0.515	0.912	0.983	0.678	0.904	0.985	0.774	0.959
Sequence	T	L	D	T	I	E	K	E	I	E	G	E	F	E	S	L	F	Q	E	A	S	
Posterior probability	0.448	0.62	0.697	0.351	0.866	0.69	0.994	0.626	0.529	0.542	0.655	0.946	0.969	0.577	0.946	0.983	0.643	0.902	0.978	0.82	0.931	
Sequence																						
Posterior probability																						
Sequence																						
Posterior probability																						
