# Supplementary material for: Functional and structural characterization of F1 ‐ATPase with common ancestral core domains in stator ring
Source: Protein Sci. 2025 Oct 23;34(11):e70345. doi: 10.1002/pro.70345 (PMC12550136; doi:10.1002/pro.70345)
Supplement: Supplementary file 10 — Data S10: Sequence_F‐type_alpha. [file PRO-34-e70345-s008.pdf]

Subunit	Sequence Names	Domain	Phylum or Kingdom	Species
F_alpha	Homo_sapiens_NP_001001937	Bacteria	proteobacteria	Agrobacterium fabrum
F_alpha	Danio_rerio_NP_001070823	Bacteria	Cyanobacteria	Anabaena variabilis
F_alpha	Branchiostoma_floridae_XP_002601598	Bacteria	Aquificae	Aquifex aeolicus
F_alpha	Hydra_vulgaris_XP_002163780	Eukarya	Viridiplantae	Arabidopsis thaliana
F_alpha	Caenorhabditis_elegans_Q9XXK1	Eukarya	Opisthokonta	Aspergillus niger
F_alpha	Brugia_malayi_XP_001900975	Eukarya	stramenopiles	Aureococcus anophagefferens
F_alpha	Ciona_intestinalis_NP_001027729	Eukarya	Opisthokonta	Botrytis cinerea
F_alpha	Paracoccus_denitrificans_WP_104491104.1	Eukarya	Opisthokonta	Branchiostoma floridae
F_alpha	Agrobacterium_fabrum_str._C58_Q8UC74.1	Eukarya	Opisthokonta	Brugia malayi
F_alpha	Rhodospirillum_rubrum_WP_011388979	Eukarya	Opisthokonta	Caenorhabditis elegans
F_alpha	Porphyra_umbilicalis_OSX79899.1	Bacteria	Deferribacteres	Calditerrivibrio nitroreducens
F_alpha	Chondrus_crispus_XP_005718938	Bacteria	Calditrichaeota	Caldithrix abyssi
F_alpha	Neurospora_crassa_OR74A_XP_965645	Bacteria	Calditrichaeota	Caldithrix sp.
F_alpha	Botrytis_cinerea_T4_CCD47511	Bacteria	proteobacteria	Campylobacter jejuni
F_alpha	Aspergillus_niger_CBS_513.88_XP_001398041	Bacteria	unclassified Bacteria	candidate division CPR2 bacterium GW2011 GWC2 39 10
F_alpha	Ixodes_scapularis_XP_002408500	Bacteria	unclassified Bacteria	candidate division CPR2 bacterium GW2011 GWC2 39 35
F_alpha	Rickettsia_prowazekii_WP_004599658	Bacteria	unclassified Bacteria	candidate division Hyd24-12 bacterium Vib 1
F_alpha	Kosmotoga_olearia_WP_012744732	Bacteria	unclassified Bacteria	candidate division KSB1 4572 119
F_alpha	Mesoaciditoga_lauensis_WP_036221456	Bacteria	unclassified Bacteria	candidate division KSB1 bacterium 4484 87
F_alpha	Candidatus_Marinimicrobia_bacterium_TMED108_OUV42920.1	Bacteria	unclassified Bacteria	candidate division KSB1 bacterium RBG 16 48 16
F_alpha	Candidatus_Marinimicrobia_bacterium_CG_4_10_14_0_2_um_filter_48_9_PIZ69981.1	Bacteria	unclassified Bacteria	candidate division NC10 bacterium CSP1-5
F_alpha	Thermosulfidibacter_takaii_WP_068549668.1	Bacteria	unclassified Bacteria	candidate division NC10 bacterium RIFCSPLOWO2 02
F_alpha	Nitrospinae_bacterium_RIFCSPHIGHO2_02_39_11_OGV97759.1	Bacteria	unclassified Bacteria	candidate division WOR-1 bacterium RIFOXYA12 FULL 52 29
F_alpha	Candidatus_Schekmanbacteria_bacterium_RIFCSPLOWO2_02_FULL_38_14_OGL49358.1	Bacteria	unclassified Bacteria	candidate division WOR-1 bacterium RIFOXYA2 FULL 36 21
F_alpha	Nitrospinae_bacterium_CG11_big_fil_rev_8_21_14_0_20_45_15_PIQ99635.1	Bacteria	unclassified Bacteria	candidate division WOR-1 bacterium RIFOXYC2 FULL 41 25
F_alpha	Nitrospina_gracilis_WP_005011070	Bacteria	unclassified Bacteria	candidate division Zixibacteria bacterium CG 4 9 14 3 um filter 46 8
F_alpha	Denitrovibrio_acetiphilus_WP_013010192	Bacteria	unclassified Bacteria	candidate division Zixibacteria bacterium HGW-Zixibacteria-1
F_alpha	Deferribacter_desulfuricans_WP_013008585	Bacteria	unclassified Bacteria	candidate division Zixibacteria bacterium RBG-1
F_alpha	Calditerrivibrio_nitroreducens_WP_013451708	Bacteria	unclassified Bacteria	candidate division Zixibacteria bacterium SM23 73 2
F_alpha	Desulfurispirillum_indicum_WP_013506843	Bacteria	unclassified Bacteria	candidate division Zixibacteria bacterium SM23 81
F_alpha	Chrysiogenes_arsenatis_WP_027389803	Bacteria	unclassified Bacteria	Candidatus Aminicenantes bacterium RBG 16
F_alpha	Candidatus_Rokubacteria_bacterium_GWA2_70_23_OGK81177.1	Bacteria	unclassified Bacteria	Candidatus Aminicenantes bacterium RBG 19FT COMBO 58 17
F_alpha	Candidatus_Rokubacteria_bacterium_13_2_20CM_2_64_8_OLB36527.1	Bacteria	unclassified Bacteria	Candidatus Beckwithbacteria bacterium CG 4
F_alpha	Candidatus_Rokubacteria_bacterium_13_1_40CM_68_15_OLC01262.1	Bacteria	unclassified Bacteria	Candidatus Beckwithbacteria bacterium RIFCSPLOWO2
F_alpha	Candidatus_Methylomirabilis_oxyfera_CBE70035	Bacteria	unclassified Bacteria	Candidatus Blackallbacteria bacterium
F_alpha	candidate_division_NC10_bacterium_RIFCSPLOWO2_02_FULL_66_22_OGB88881.1	Bacteria	unclassified Bacteria	Candidatus Buchananbacteria bacterium RIFCSPHIGHO2 01 FULL 44 11
F_alpha	candidate_division_NC10_bacterium_CSP1-5_KRT70774	Bacteria	unclassified Bacteria	Candidatus Buchananbacteria bacterium RIFCSPHIGHO2 02 FULL 39 17
F_alpha	candidate_division_Zixibacteria_bacterium_SM23_81_KPL16455	Bacteria	Cloacimonetes	Candidatus Cloacimonas sp. SDB
F_alpha	Thermodesulfovibrio_yellowstonii_WP_012545369	Bacteria	unclassified Bacteria	Candidatus Dadabacteria bacterium CSP1-2
F_alpha	Candidatus_Nitrospira_defluvii_WP_013246975	Bacteria	unclassified Bacteria	Candidatus Dadabacteria bacterium RIFCSPHIGHO2 12 FULL 53 21
F_alpha	Candidatus_Dadabacteria_bacterium_RIFCSPHIGHO2_12_FULL_53_21_OGE18462.1	Bacteria	unclassified Bacteria	Candidatus Daviesbacteria bacterium GW2011 GWA1 41 61
F_alpha	Candidatus_Dadabacteria_bacterium_CSP1-2_KRT65371.1	Bacteria	unclassified Bacteria	Candidatus Daviesbacteria bacterium GW2011 GWA2 38 24
F_alpha	Desulfovibrio_vulgaris_WP_010938078	Bacteria	unclassified Bacteria	Candidatus Desantisbacteria bacterium
F_alpha	candidate_division_Zixibacteria_bacterium_SM23_73_2_KPL03769	Bacteria	unclassified Bacteria	Candidatus Doudnabacteria bacterium RIFCSPHIGHO2 01 FULL 41 86
F_alpha	candidate_division_Zixibacteria_bacterium_RBG-1_EQB62571	Bacteria	unclassified Bacteria	Candidatus Doudnabacteria bacterium RIFCSPHIGHO2 12 FULL 48 11
F_alpha	candidate_division_Zixibacteria_bacterium_HGW-Zixibacteria-1_PKK82302.1	Bacteria	unclassified Bacteria	Candidatus Eisenbacteria bacterium RBG 16 71 46
F_alpha	candidate_division_Zixibacteria_bacterium_CG_4_9_14_3_um_filter_46_8_PJA29144.1	Bacteria	unclassified Bacteria	Candidatus Goldbacteria bacterium HGW-Goldbacteria-1
F_alpha	Candidatus_Eisenbacteria_bacterium_RBG_16_71_46_OGF04986.1	Bacteria	unclassified Bacteria	Candidatus Gottesmanbacteria bacterium CG11

F_alpha	Limnochorda_pilosa_BAS28917	Bacteria	unclassified Bacteria	Candidatus Gottesmanbacteria bacterium GW2011 GWA2 42 18
F_alpha	candidate_division_KSB1_bacterium_RBG_16_48_16_OGC09761	Bacteria	unclassified Bacteria	Candidatus Gottesmanbacteria bacterium GW2011 GWA2 43 14
F_alpha	candidate_division_KSB1_bacterium_4484_87_OQX83156	Bacteria	unclassified Bacteria	Candidatus Gottesmanbacteria bacterium GW2011 GWB1 43 11
F_alpha	candidate_division_KSB1_4572_119_OQX96026	Bacteria	unclassified Bacteria	Candidatus Gottesmanbacteria bacterium RBG 16
F_alpha	Candidatus_Cloacimonas_sp._SDB_KQC03754	Bacteria	unclassified Bacteria	Candidatus Gribaldobacteria bacterium CG 4
F_alpha	Caldithrix_sp._RBG_13_44_9_OGB69842.1	Bacteria	unclassified Bacteria	Candidatus Howlettbacteria bacterium CG23 combo of CG06-09 8 20 14 all 37 9
F_alpha	Caldithrix_abyssi_DSM_13497_EHO39882	Bacteria	unclassified Bacteria	Candidatus Komeilibacteria bacterium RIFOXYC2 FULL 45 12
F_alpha	Omnitrophica_bacterium_RIFCSPLOWO2_02_FULL_44_11_OGX04505.1	Bacteria	proteobacteria	Candidatus Lambdaproteobacteria bacterium RIFOXYC1 FULL 56 13
F_alpha	Candidatus_Omnitrophica_bacterium_CG11_big_fil_rev_8_21_14_0_20_45_26_PIQ85514.1	Bacteria	proteobacteria	Candidatus Lambdaproteobacteria bacterium RIFOXYD2 FULL 50 16
F_alpha	Omnitrophica_WOR_2_bacterium_GWA2_63_20_OGX12395.1	Bacteria	unclassified Bacteria	Candidatus Lindowbacteria bacterium RIFCSPLOWO2
F_alpha	Candidatus_Aminicenantes_bacterium_RBG_19FT_COMBO_58_17_OGD40035.1	Bacteria	unclassified Bacteria	Candidatus Lloydbacteria bacterium
F_alpha	Candidatus_Aminicenantes_bacterium_RBG_16_63_16_OGD19775.1	Bacteria	Fibrobacteres/Acidobacteria	Candidatus Marinimicrobia bacterium CG 4
F_alpha	Campylobacter_jejuni_subsp._jejuni_NCTC_11168-mfK12E5_AHK56461	Bacteria	Fibrobacteres/Acidobacteria	Candidatus Marinimicrobia bacterium TMED108
F_alpha	Helicobacter_pylori_WP_046656269	Bacteria	unclassified Bacteria	Candidatus Methylomirabilis oxyfera
F_alpha	Hydrogenobacter_thermophilus_WP_012962902	Bacteria	unclassified Bacteria	Candidatus Moranbacteria bacterium CG08
F_alpha	Aquifex_aeolicus_WP_010880405	Bacteria	unclassified Bacteria	Candidatus Moranbacteria bacterium GW2011
F_alpha	Desulfurobacterium_thermolithotrophum_WP_013638838	Bacteria	Nitrospirae	Candidatus Nitrospira defluvii
F_alpha	Chloracidobacterium_thermophilum_WP_014100113	Bacteria	unclassified Bacteria	Candidatus Nomurabacteria bacterium GW2011 GWB1 40 11
F_alpha	Candidatus_Lambdaproteobacteria_bacterium_RIFOXYD2_FULL_50_16_OGG96484.1	Bacteria	unclassified Bacteria	Candidatus Nomurabacteria bacterium GW2011 GWD2 36 14
F_alpha	Candidatus_Lambdaproteobacteria_bacterium_RIFOXYC1_FULL_56_13_OGH03654.1	Bacteria	unclassified Bacteria	Candidatus Nomurabacteria bacterium GW2011 GWF2 30 133
F_alpha	Candidatus_Goldbacteria_bacterium_HGW-Goldbacteria-1_PKL90500.1	Bacteria	unclassified Bacteria	Candidatus Nomurabacteria bacterium GW2011 GWF2 40 12
F_alpha	Zea_mays_subsp._mays_AGV02737	Bacteria	unclassified Bacteria	Candidatus Nomurabacteria bacterium RIFCSPHIGHO2 02 41 18
F_alpha	Oryza_sativa_Japonica_Group_XP_015613749	Bacteria	Omnitrophica	Candidatus Omnitrophica bacterium
F_alpha	Spinacia_oleracea_P06450.1	Bacteria	Peregrinibacteria	Candidatus Peregrinibacteria bacterium CG 4
F_alpha	Populus_trichocarpa_YP_001109484	Bacteria	unclassified Bacteria	Candidatus Raymondbacteria bacterium
F_alpha	Arabidopsis_thaliana_NP_051044	Bacteria	unclassified Bacteria	Candidatus Roizmanbacteria bacterium CG 4 9 14 0 2 um filter 38 17
F_alpha	Physcomitrella_patens_subsp._patens_NP_904216	Bacteria	unclassified Bacteria	Candidatus Roizmanbacteria bacterium RIFCSPHIGHO2
F_alpha	Chlorella_variabilis_YP_004347819	Bacteria	unclassified Bacteria	Candidatus Rokubacteria bacterium 13 1 40CM
F_alpha	Thermosynechococcus_elongatus_WP_011056290	Bacteria	unclassified Bacteria	Candidatus Rokubacteria bacterium 13 2 20CM
F_alpha	Synechococcus_elongatus_PCC_6301_P08449	Bacteria	unclassified Bacteria	Candidatus Rokubacteria bacterium GWA2
F_alpha	Prochlorococcus_marinus_subsp._marinus_str._CCMP1375_Q7VA63	Bacteria	unclassified Bacteria	Candidatus Saganbacteria bacterium
F_alpha	Anabaena_variabilis_ATCC_29413_Q3M9W0	Bacteria	unclassified Bacteria	Candidatus Schekmanbacteria bacterium RIFCSPLOWO2
F_alpha	Chlamydomonas_reinhardtii_P26526	Bacteria	unclassified Bacteria	Candidatus Taylorbacteria bacterium RIFCSPLOWO2 12
F_alpha	Thalassiosira_pseudonana_CCMP1335_XP_002297546	Bacteria	unclassified Bacteria	Candidatus Uhrbacteria bacterium CG10
F_alpha	Porphyra_umbilicalis_YP_009413255.1	Bacteria	unclassified Bacteria	Candidatus Uhrbacteria bacterium GW2011 GWC2
F_alpha	Chondrus_crispus_YP_007627435	Bacteria	unclassified Bacteria	Candidatus Uhrbacteria bacterium GW2011 GWE2
F_alpha	Guillardia_theta_O78475	Bacteria	unclassified Bacteria	Candidatus Uhrbacteria bacterium RIFCSPHIGHO2
F_alpha	Chrysochromulina_sp._CCMP291_AHY04322	Bacteria	unclassified Bacteria	Candidatus Uhrbacteria bacterium RIFOXYC2
F_alpha	Galdieria_sulphuraria_YP_009051122	Bacteria	unclassified Bacteria	Candidatus Wallbacteria bacterium GWC2
F_alpha	Galdieria_sulphuraria_P35009	Bacteria	unclassified Bacteria	Candidatus Wolfebacteria bacterium GW2011 GWD2 47 17
F_alpha	Aureococcus_anophagefferens_YP_003002038	Bacteria	unclassified Bacteria	Candidatus Wolfebacteria bacterium GW2011 GWE2 44 13
F_alpha	Candidatus_Blackallbacteria_bacterium_CG13_big_fil_rev_8_21_14_2_50_49_14_PIW49899.1	Bacteria	unclassified Bacteria	Candidatus Woykebacteria bacterium GWA1
F_alpha	Candidatus_Desantisbacteria_bacterium_CG_4_8_14_3_um_filter_40_12_PIX16795.1	Eukarya	Viridiplantae	Chlamydomonas reinhardtii
F_alpha	Clostridium_acetobutylicum_WP_010966151	Bacteria	Fibrobacteres/Acidobacteria	Chloracidobacterium thermophilum
F_alpha	Candidatus_Wallbacteria_bacterium_GWC2_49_35_OGM05274	Eukarya	Viridiplantae	Chlorella variabilis
F_alpha	candidate_division_Hyd24-12_bacterium_Vib_1_KZD18817	Eukarya	Rhodphyta	Chondrus crispus
F_alpha	Candidatus_Lindowbacteria_bacterium_RIFCSPLOWO2_12_FULL_62_27_OGH61313.1	Eukarya	Rhodphyta	Chondrus crispus
F_alpha	Candidatus_Raymondbacteria_bacterium_RIFOXYA2_FULL_49_16_OGJ89254.1	Bacteria	Chrysiogenetes	Chrysiogenes arsenatis
F_alpha	Lentisphaerae_bacterium_RIFOXYC12_FULL_60_16_OGV66388	Eukarya	Haptophyceae	Chrysochromulina sp.
F_alpha	Candidatus_Daviesbacteria_bacterium_GW2011_GWA2_38_24_KKQ66449.1	Eukarya	Opisthokonta	Ciona intestinalis

F_alpha	Candidatus_Daviesbacteria_bacterium_GW2011_GWA1_41_61_KKS15022.1	Bacteria	Firmicutes	Clostridium acetobutylicum
F_alpha	Candidatus_Woykebacteria_bacterium_GWA1_44_8_OGY21193.1	Eukarya	Opisthokonta	Danio rerio
F_alpha	Candidatus_Wolfebacteria_bacterium_GW2011_GWE2_44_13_KKT43603.1	Bacteria	Deferribacteres	Deferribacter desulfuricans
F_alpha	Candidatus_Wolfebacteria_bacterium_GW2011_GWD2_47_17_KKU66431.1	Bacteria	Deferribacteres	Denitrovibrio acetiphilus
F_alpha	Candidatus_Moranbacteria_bacterium_CG08_land_8_20_14_0_20_34_16_PIU08345.1	Bacteria	proteobacteria	Desulfovibrio vulgaris str. Hildenborough
F_alpha	Candidatus_Moranbacteria_bacterium_GW2011_GWF1_34_10_KKP58980.1	Bacteria	Chrysiogenetes	Desulfurispirillum indicum
F_alpha	Candidatus_Doudnabacteria_bacterium_RIFCSPHIGHO2_12_FULL_48_11_OGE95452.1	Bacteria	Aquificae	Desulfurobacterium thermolithotrophum
F_alpha	Candidatus_Doudnabacteria_bacterium_RIFCSPHIGHO2_01_FULL_41_86_OGE74118.1	Eukarya	Rhodphyta	Galdieria sulphuraria
F_alpha	Candidatus_Komeilibacteria_bacterium_RIFOXYC2_FULL_45_12_OGY95607.1	Eukarya	Rhodphyta	Galdieria sulphuraria
F_alpha	Candidatus_Buchananbacteria_bacterium_RIFCSPHIGHO2_02_FULL_39_17_OGY48702.1	Eukarya	Cryptophyta	Guillardia theta
F_alpha	Candidatus_Buchananbacteria_bacterium_RIFCSPHIGHO2_01_FULL_44_11_OGY45438.1	Bacteria	proteobacteria	Helicobacter pylori J99
F_alpha	Candidatus_Uhrbacteria_bacterium_RIFOXYC2_FULL_47_19_OGM00383.1	Eukarya	Opisthokonta	Homo sapiens
F_alpha	Candidatus_Uhrbacteria_bacterium_RIFCSPHIGHO2_01_FULL_63_20_OGL66462.1	Eukarya	Opisthokonta	Hydra vulgaris
F_alpha	Candidatus_Uhrbacteria_bacterium_GW2011_GWC2_41_11_KKR86811.1	Bacteria	Aquificae	Hydrogenobacter thermophilus
F_alpha	Candidatus_Uhrbacteria_bacterium_GW2011_GWE2_46_68_KKU40986.1	Eukarya	Opisthokonta	Ixodes scapularis
F_alpha	Candidatus_Lloydbacteria_bacterium_CG22_combo_CG10-13_8_21_14_all_47_15_PIP73635.1	Bacteria	Thermotogae	Kosmotoga olearia
F_alpha	Candidatus_Howlettbacteria_bacterium_CG23_combo_of_CG06-09_8_20_14_all_37_9_PIP30538.1	Bacteria	Lentisphaerae	Lentisphaerae bacterium
F_alpha	candidate_division_CPR2_bacterium_GW2011_GWC2_39_10_KKQ95282	Bacteria	Nitrospirae	Leptospirillum ferrooxidans
F_alpha	candidate_division_CPR2_bacterium_GW2011_GWC2_39_35_KKR18685	Bacteria	Firmicutes	Limnochorda pilosa
F_alpha	Candidatus_Peregrinibacteria_bacterium_CG_4_9_14_0_2_um_filter_53_11_PJC36773.1	Bacteria	Thermotogae	Mesoaciditoga lauensis
F_alpha	Candidatus_Taylorbacteria_bacterium_RIFCSPLOWO2_12_FULL_43_20_OHA41483.1	Bacteria	Microgenomates	Microgenomates bacterium OLB23
F_alpha	Candidatus_Nomurabacteria_bacterium_RIFCSPHIGHO2_02_41_18_OGI72502.1	Eukarya	Opisthokonta	Neurospora crassa
F_alpha	Candidatus_Nomurabacteria_bacterium_GW2011_GWF2_40_12_KKR43900.1	Bacteria	Nitrospinae/Tectomicrobia group	Nitrospina gracilis
F_alpha	Candidatus_Nomurabacteria_bacterium_GW2011_GWB1_40_11_KKR38904.1	Bacteria	Nitrospinae/Tectomicrobia group	Nitrospinae bacterium CG11 big fil rev 8 21 14 0 20 45 16
F_alpha	Candidatus_Nomurabacteria_bacterium_GW2011_GWF2_30_133_KKP24631.1	Bacteria	Nitrospinae/Tectomicrobia group	Nitrospinae bacterium RIFCSPHIGHO2 02 39 11
F_alpha	Candidatus_Nomurabacteria_bacterium_GW2011_GWD2_36_14_KKP96649.1	Bacteria	Omnitrophica	Omnitrophica bacterium RIFCSPLOWO2
F_alpha	Candidatus_Gribaldobacteria_bacterium_CG_4_10_14_0_2_um_filter_41_16_PJA01437.1	Bacteria	Omnitrophica	Omnitrophica WOR 2 bacterium
F_alpha	Candidatus_Uhrbacteria_bacterium_CG10_big_fil_rev_8_21_14_0_10_50_16_PIR47915.1	Eukarya	Viridiplantae	Oryza sativa japonica
F_alpha	Candidatus_Gottesmanbacteria_bacterium_GW2011_GWA2_43_14_KKS98509.1	Bacteria	proteobacteria	Paracoccus denitrificans
F_alpha	Candidatus_Gottesmanbacteria_bacterium_GW2011_GWA2_42_18_KKS46806.1	Eukarya	Viridiplantae	Physcomitrella patens subsp. patens
F_alpha	Candidatus_Gottesmanbacteria_bacterium_CG11_big_fil_rev_8_21_14_0_20_37_11_PIR07964.1	Eukarya	Viridiplantae	Populus trichocarpa
F_alpha	Candidatus_Gottesmanbacteria_bacterium_GW2011_GWB1_43_11_KKS86374.1	Eukarya	Rhodphyta	Porphyra umbilicalis
F_alpha	Candidatus_Gottesmanbacteria_bacterium_RBG_16_52_11_OGG02393.1	Eukarya	Rhodphyta	Porphyra umbilicalis
F_alpha	Candidatus_Roizmanbacteria_bacterium_CG_4_9_14_0_2_um_filter_38_17_PJC31479.1	Bacteria	Cyanobacteria	Prochlorococcus marinus subsp. Marinus
F_alpha	Leptospirillum_ferrooxidans_WP_014448291	Bacteria	Planctomycetes	Rhodopirellula baltica
F_alpha	Rhodopirellula_baltica_WP_007334261	Bacteria	proteobacteria	Rhodospirillum rubrum
F_alpha	Candidatus_Beckwithbacteria_bacterium_RIFCSPLOWO2_02_FULL_47_23_OGD61454.1	Bacteria	proteobacteria	Rickettsia prowazekii
F_alpha	Candidatus_Beckwithbacteria_bacterium_CG_4_10_14_0_2_um_filter_47_25_PJA22937.1	Eukarya	Viridiplantae	Spinacia oleracea
F_alpha	Microgenomates_bacterium_OLB23_KXK11275	Bacteria	Cyanobacteria	Synechococcus elongatus
F_alpha	Candidatus_Roizmanbacteria_bacterium_RIFCSPHIGHO2_02_FULL_38_11_OGK23344.1	Eukarya	stramenopiles	Thalassiosira pseudonana
F_alpha	Candidatus_Saganbacteria_bacterium_CG08_land_8_20_14_0_20_45_16_PIS31072.1	Bacteria	Nitrospirae	Thermodesulfovibrio yellowstonii
F_alpha	candidate_division_WOR-1_bacterium_RIFOXYC2_FULL_41_25_OGC32824.1	Bacteria	Aquificae	Thermosulfidibacter takaii
F_alpha	candidate_division_WOR-1_bacterium_RIFOXYA2_FULL_36_21_OGC05663.1	Bacteria	Cyanobacteria	Thermosynechococcus elongatus
F_alpha	candidate_division_WOR-1_bacterium_RIFOXYA12_FULL_52_29_OGC18099.1	Eukarya	Viridiplantae	Zea mays
