# Supplementary material for: Functional and structural characterization of F1 ‐ATPase with common ancestral core domains in stator ring
Source: Protein Sci. 2025 Oct 23;34(11):e70345. doi: 10.1002/pro.70345 (PMC12550136; doi:10.1002/pro.70345)
Supplement: Supplementary file 11 — Data S11: Sequence_F‐type_beta. [file PRO-34-e70345-s016.pdf]

Subunit	Sequence Names	Domain	Phylum or Kingdom	Species
F_beta	Homo_sapiens_NP_001677	Bacteria	Acetothermia	Acetothermia bacterium
F_beta	Danio_rerio_NP_001019600	Bacteria	Fibrobacteres/Acidobacteria	Acidobacterium capsulatum
F_beta	Danio_rerio_NP_001083034	Eukarya	Opisthokonta	Amphimedon queenslandica
F_beta	Branchiostoma_floridae_XP_002595117	Bacteria	Cyanobacteria	Anabaena variabilis
F_beta	Strongylocentrotus_purpuratus_NP_001116974	Eukarya	Viridiplantae	Arabidopsis thaliana
F_beta	Amphimedon_queenslandica_XP_003383663	Eukarya	stramenopiles	Aureococcus anophagefferens
F_beta	Schistosoma_mansoni_CCD79896	Eukarya	Opisthokonta	Branchiostoma floridae
F_beta	Rhodospirillum_rubrum_WP_011388981	Bacteria	Deferribacteres	Calditerrivibrio nitroreducens
F_beta	Paracoccus_denitrificans_PD1222_ABL71884.1	Bacteria	Calditrichaeota	Caldithrix abyssi
F_beta	Magnetococcus_marinus_WP_011714999	Bacteria	Calditrichaeota	Caldithrix sp.
F_beta	Chrysochromulina_sp._CCMP291_KOO26830	Bacteria	unclassified Bacteria	candidate division CPR2 bacterium GW2011 GWC2 39 10
F_beta	Stylonychia_lemnae_CDW83582	Bacteria	unclassified Bacteria	candidate division CPR2 bacterium GW2011 GWC2 39 35
F_beta	Oxytricha_trifallax_EJY84535	Bacteria	unclassified Bacteria	candidate division KSB1 4572 119
F_beta	Paramecium_tetraurelia_strain_d4-2_XP_001437608	Bacteria	unclassified Bacteria	candidate division KSB1 bacterium RBG 16 48 16
F_beta	Tetrahymena_thermophila_SB210_EAR84968	Bacteria	unclassified Bacteria	candidate division NC10 bacterium CSP1-5
F_beta	Holophaga_foetida_WP_005038027	Bacteria	unclassified Bacteria	candidate division WWE3 bacterium
F_beta	Zea_mays_subsp._mays_AGV02747	Bacteria	unclassified Bacteria	candidate division Zixibacteria bacterium CG 4 9 14 3 um filter 46 8
F_beta	Oryza_sativa_Japonica_Group_NP_039390	Bacteria	unclassified Bacteria	Candidatus Aminicenantes bacterium RBG 16
F_beta	Spinacia_oleracea_6FKF_F	Bacteria	unclassified Bacteria	Candidatus Aminicenantes bacterium RBG 19FT COMBO 65 30
F_beta	Populus_trichocarpa_YP_001109508	Bacteria	unclassified Bacteria	Candidatus Beckwithbacteria bacterium CG 4
F_beta	Arabidopsis_thaliana_NP_051066	Bacteria	unclassified Bacteria	Candidatus Beckwithbacteria bacterium CG23
F_beta	Physcomitrella_patens_subsp._patens_NP_904195	Bacteria	unclassified Bacteria	Candidatus Beckwithbacteria bacterium GW2011
F_beta	Volvox_carteri_f._nagariensis_ACY05995	Bacteria	unclassified Bacteria	Candidatus Beckwithbacteria bacterium RIFCSPLOWO2
F_beta	Chlamydomonas_reinhardtii_P06541	Bacteria	unclassified Bacteria	Candidatus Chisholmbacteria bacterium RIFCSPHIGHO2 01 FULL 48 12
F_beta	Chlorella_variabilis_YP_004347820	Bacteria	unclassified Bacteria	Candidatus Chisholmbacteria bacterium RIFCSPLOWO2 01 FULL 50 28
F_beta	Galdieria_sulphuraria_Q08807	Bacteria	Candidatus Kryptonia	Candidatus Chrysopegis kryptomonas
F_beta	Porphyra_umbilicalis_ASN78733.1	Bacteria	Cloacimonetes	Candidatus Cloacimonas sp. SDB
F_beta	Chondrus_crispus_CCP38166	Bacteria	Cloacimonetes	Candidatus Cloacimonetes bacterium
F_beta	Guillardia_theta_O78491	Bacteria	unclassified Bacteria	Candidatus Curtissbacteria bacterium GW2011
F_beta	Aureococcus_anophagefferens_ACS36880	Bacteria	unclassified Bacteria	Candidatus Curtissbacteria bacterium RIFCSPLOWO2
F_beta	Thalassiosira_pseudonana_CCMP1335_XP_002297536	Bacteria	unclassified Bacteria	Candidatus Dadabacteria bacterium CSP1-2
F_beta	Chrysochromulina_sp._CCMP291_AHY04402	Bacteria	unclassified Bacteria	Candidatus Dadabacteria bacterium RIFCSPHIGHO2 12 FULL 53 21
F_beta	Cyanidioschyzon_merolae_strain_10D_NP_849101	Bacteria	unclassified Bacteria	Candidatus Daviesbacteria bacterium GW2011 GWA2 38 24
F_beta	Thermosynechococcus_elongatus_WP_011056375	Bacteria	unclassified Bacteria	Candidatus Daviesbacteria bacterium RIFCSPHIGHO2 12 FULL 37 16
F_beta	Anabaena_variabilis_ATCC_29413_ABA21916	Bacteria	unclassified Bacteria	Candidatus Daviesbacteria bacterium RIFCSPLOWO2 12 FULL 38 10
F_beta	Synechococcus_elongatus_PCC_6301_BAD79977	Bacteria	unclassified Bacteria	Candidatus Eisenbacteria bacterium RBG 16 71 46
F_beta	Gloeobacter_violaceus_WP_011142564	Bacteria	Nitrospinae/Tectomicrobia group	Candidatus Entotheonella palauensis
F_beta	Candidatus_Melainabacteria_bacterium_MEL.A1_AOR38416.1	Bacteria	unclassified Bacteria	Candidatus Firestonebacteria bacterium RIFOXYD2 FULL 39 29
F_beta	Candidatus_Gastranaerophilales_bacterium_HUM_20_DAB18663	Bacteria	unclassified Bacteria	Candidatus Fischerbacteria bacterium RBG 13 37 8
F_beta	Candidatus_Gastranaerophilales_bacterium_HUM_5_DAA90667	Bacteria	unclassified Bacteria	Candidatus Gastranaerophilales bacterium HUM 20
F_beta	Candidatus_Gastranaerophilales_bacterium_HUM_6_DAA91668	Bacteria	unclassified Bacteria	Candidatus Gastranaerophilales bacterium HUM 5
F_beta	Candidatus_Gastranaerophilales_bacterium_HUM_9_DAA96029	Bacteria	unclassified Bacteria	Candidatus Gastranaerophilales bacterium HUM 6
F_beta	Candidatus_Melainabacteria_bacterium_GWA2_34_9_OGH96909.1	Bacteria	unclassified Bacteria	Candidatus Gastranaerophilales bacterium HUM 9
F_beta	Candidatus_Melainabacteria_bacterium_RIFCSPLOWO2_12_FULL_35_11_OGI06699.1	Bacteria	unclassified Bacteria	Candidatus Gottesmanbacteria bacterium GW2011 GWA2 42 18
F_beta	Candidatus_Melainabacteria_bacterium_RIFCSPHIGHO2_02_FULL_34_12_OGI20559.1	Bacteria	unclassified Bacteria	Candidatus Gottesmanbacteria bacterium GW2011 GWA2 43 14
F_beta	Candidatus_Nitrospira_defluvii_CBK40151	Bacteria	unclassified Bacteria	Candidatus Handelsmanbacteria bacterium RIFCSPLOWO2 12 FULL 64 10
F_beta	Candidatus_Rokubacteria_bacterium_RIFCSPLOWO2_02_FULL_72_37_OGL09191.1	Bacteria	unclassified Bacteria	Candidatus Howlettbacteria bacterium CG23 combo of CG06-09 8 20 14 all 37 9
F_beta	Candidatus_Rokubacteria_bacterium_13_1_40CM_68_15_OLC01260.1	Bacteria	unclassified Bacteria	Candidatus Hydrogenedentes bacterium CG1 02 42 14
F_beta	Candidatus_Rokubacteria_bacterium_GWA2_70_23_OGK81179.1	Bacteria	unclassified Bacteria	Candidatus Kapabacteria sp. 59-99

F_beta	Candidatus_Methylomirabilis_oxyfera_CBE70037	Bacteria	proteobacteria	Candidatus Lambdaproteobacteria bacterium RIFOXYC1 FULL 56 13
F_beta	candidate_division_NC10_bacterium_CSP1-5_KRT70772	Bacteria	proteobacteria	Candidatus Lambdaproteobacteria bacterium RIFOXYD2 FULL 50 16
F_beta	Nitrospinae_bacterium_RIFCSPHIGHO2_02_39_11_OGV97757.1	Bacteria	Latescibacteria	Candidatus Latescibacteria bacterium 4484 107
F_beta	Candidatus_Schekmanbacteria_bacterium_RIFCSPLOWO2_02_FULL_38_14_OGL49356.1	Bacteria	unclassified Bacteria	Candidatus Lindowbacteria bacterium RIFCSPLOWO2
F_beta	Candidatus_Schekmanbacteria_bacterium_GWA2_38_11_OGL38320.1	Bacteria	unclassified Bacteria	Candidatus Liptonbacteria bacterium
F_beta	Nitrospinae_bacterium_CG11_big_fil_rev_8_21_14_0_20_56_8_PIQ96844.1	Bacteria	unclassified Bacteria	Candidatus Margulisbacteria bacterium GWF2 38 17
F_beta	Nitrospinae_bacterium_CG11_big_fil_rev_8_21_14_0_20_45_15_PIQ99633.1	Bacteria	Fibrobacteres/Acidobacteria	Candidatus Marinimicrobia bacterium CG 4
F_beta	Nitrospina_gracilis_3/211_CCQ91838	Bacteria	unclassified Bacteria	Candidatus Melainabacteria bacterium GWA2
F_beta	Caldithrix_sp._RBG_13_44_9_OGB69844.1	Bacteria	unclassified Bacteria	Candidatus Melainabacteria bacterium MEL.A1
F_beta	Caldithrix_abyssi_DSM_13497_EHO39880	Bacteria	unclassified Bacteria	Candidatus Melainabacteria bacterium RIFCSPHIGHO2 02 FULL 34 12
F_beta	Candidatus_Entotheonella_palauensis_WP_089944236	Bacteria	unclassified Bacteria	Candidatus Melainabacteria bacterium RIFCSPLOWO2 12 FULL 35 11
F_beta	Denitrovibrio_acetiphilus_WP_013010194	Bacteria	unclassified Bacteria	Candidatus Methylomirabilis oxyfera
F_beta	Deferribacter_desulfuricans_WP_013008583	Bacteria	Nitrospirae	Candidatus Nitrospira defluvii
F_beta	Calditerrivibrio_nitroreducens_WP_013451706	Bacteria	unclassified Bacteria	Candidatus Pacebacteria bacterium CG 4
F_beta	Candidatus_Chrysopegis_kryptomonas_CUS95797	Bacteria	unclassified Bacteria	Candidatus Pacebacteria bacterium CG10
F_beta	Melioribacter_roseus_P3M-2_AFN75592	Bacteria	unclassified Bacteria	Candidatus Pacebacteria bacterium GW2011 GWA1 46 10
F_beta	Ignavibacterium_album_WP_014560501	Bacteria	unclassified Bacteria	Candidatus Pacebacteria bacterium GW2011 GWF2 38 9
F_beta	Candidatus_Kapabacteria_sp._59-99_OJX61120	Bacteria	Peregrinibacteria	Candidatus Peregrinibacteria bacterium GW2011
F_beta	Prosthecochloris_aestuarii_WP_012504653	Bacteria	Peregrinibacteria	Candidatus Peribacteria bacterium
F_beta	Chlorobium_phaeobacteroides_WP_011743961	Bacteria	unclassified Bacteria	Candidatus Roizmanbacteria bacterium CG 4 9 14 3 um filter 36 11
F_beta	Chlorobaculum_parvum_WP_012501308	Bacteria	unclassified Bacteria	Candidatus Roizmanbacteria bacterium GW2011
F_beta	Thermodesulfobacterium_geofontis_WP_013910322	Bacteria	unclassified Bacteria	Candidatus Roizmanbacteria bacterium RIFCSPHIGHO2
F_beta	Thermodesulfobacterium_commune_DSM_2178_AIH03535	Bacteria	unclassified Bacteria	Candidatus Roizmanbacteria bacterium RIFOXYA2
F_beta	Thermodesulfatator_indicus_DSM_15286_AEH45201	Bacteria	unclassified Bacteria	Candidatus Rokubacteria bacterium 13 1 40CM
F_beta	Geobacter_sulfurreducens_PCA_AAR33448	Bacteria	unclassified Bacteria	Candidatus Rokubacteria bacterium GWA2
F_beta	Candidatus_Dadabacteria_bacterium_RIFCSPHIGHO2_12_FULL_53_21_OGE18464.1	Bacteria	unclassified Bacteria	Candidatus Rokubacteria bacterium RIFCSPLOWO2
F_beta	Candidatus_Dadabacteria_bacterium_CSP1-2_KRT65373.1	Bacteria	unclassified Bacteria	Candidatus Schekmanbacteria bacterium GWA2
F_beta	candidate_division_KSB1_4572_119_OQX96024	Bacteria	unclassified Bacteria	Candidatus Schekmanbacteria bacterium RIFCSPLOWO2
F_beta	Candidatus_Lambdaproteobacteria_bacterium_RIFOXYD2_FULL_50_16_OGG96482.1	Bacteria	Fibrobacteres/Acidobacteria	Candidatus Solibacter usitatus
F_beta	Candidatus_Lambdaproteobacteria_bacterium_RIFOXYC1_FULL_56_13_OGH03656.1	Bacteria	unclassified Bacteria	Candidatus Taylorbacteria bacterium RIFCSPLOWO2 02
F_beta	Candidatus_Lindowbacteria_bacterium_RIFCSPLOWO2_12_FULL_62_27_OGH61315.1	Bacteria	unclassified Bacteria	Candidatus Woesebacteria bacterium GW2011
F_beta	Omnitrophica_bacterium_RIFCSPLOWO2_02_FULL_44_11_OGX04503.1	Bacteria	unclassified Bacteria	Candidatus Woesebacteria bacterium RIFCSPLOWO2
F_beta	Candidatus_Eisenbacteria_bacterium_RBG_16_71_46_OGF04985.1	Bacteria	unclassified Bacteria	Candidatus Woykebacteria bacterium GWA1
F_beta	candidate_division_Zixibacteria_bacterium_CG_4_9_14_3_um_filter_46_8_PJA28029.1	Bacteria	unclassified Bacteria	Candidatus Woykebacteria bacterium RBG 16
F_beta	Chthonomonas_calidirosea_WP_016481980	Bacteria	unclassified Bacteria	Candidatus Woykebacteria bacterium RBG 19FT
F_beta	Candidatus_Cloacimonas_sp._SDB_KQC03752	Bacteria	unclassified Bacteria	Candidatus Woykebacteria bacterium RIFCSPLOWO2
F_beta	candidate_division_KSB1_bacterium_RBG_16_48_16_OGC09772	Bacteria	unclassified Bacteria	Candidatus Yanofskybacteria bacterium GW2011
F_beta	Desulfovibrio_vulgaris_str._Hildenborough_YP_009996	Bacteria	unclassified Bacteria	Candidatus Yanofskybacteria bacterium RIFCSPLOWO2
F_beta	Streptobacillus_moniliformis_WP_012859038	Eukarya	Viridiplantae	Chlamydomonas reinhardtii
F_beta	Ilyobacter_polytropus_WP_013386667	Bacteria	Fibrobacteres/Acidobacteria	Chloracidobacterium thermophilum
F_beta	Fusobacterium_nucleatum_subsp._nucleatum_ATCC_25586_AAL94561	Eukarya	Viridiplantae	Chlorella variabilis
F_beta	Candidatus_Hydrogenedentes_bacterium_CG1_02_42_14_OIO34030	Bacteria	Bacteroidetes/Chlorobi group	Chlorobaculum parvum NCIB 8327
F_beta	Candidatus_Firestonebacteria_bacterium_RIFOXYD2_FULL_39_29_OGF47184.1	Bacteria	Bacteroidetes/Chlorobi group	Chlorobium phaeobacteroides DSM 266
F_beta	Thermosulfidibacter_takaii_ABI70S6_BAT71668.1	Eukarya	Rhodphyta	Chondrus crispus
F_beta	Thermobaculum_terrenum_ATCC_BAA-798_ACZ40986	Eukarya	Haptophyceae	Chrysochromulina sp.
F_beta	Candidatus_Woykebacteria_bacterium_RIFCSPLOWO2_01_FULL_43_14_OGY32208.1	Eukarya	Haptophyceae	Chrysochromulina sp.
F_beta	Candidatus_Daviesbacteria_bacterium_RIFCSPHIGHO2_12_FULL_37_16_OGE36005.1	Bacteria	Chlamydiae/Verrucomicrobia group	Chthoniobacter flavus
F_beta	Candidatus_Daviesbacteria_bacterium_GW2011_GWA2_38_24_KKQ66452.1	Bacteria	Armatinonadetes	Chthonomonas calidirosea
F_beta	Candidatus_Daviesbacteria_bacterium_RIFCSPLOWO2_12_FULL_38_10_OGE71778.1	Eukarya	Rhodphyta	Cyanidioschyzon merolae strain 10D
F_beta	Candidatus_Woykebacteria_bacterium_RBG_19FT_COMBO_43_10_OGY28297.1	Eukarya	Opisthokonta	Danio rerio
F_beta	Candidatus_Woykebacteria_bacterium_RBG_16_39_9b_OGY25146.1	Eukarya	Opisthokonta	Danio rerio

F_beta	Candidatus_Woykebacteria_bacterium_GWA1_44_8_OGY21190.1	Bacteria	Deferribacteres	Deferribacter desulfuricans
F_beta	Candidatus_Curtissbacteria_bacterium_RIFCSPLOWO2_02_FULL_42_37_OGE11612.1	Bacteria	Chloroflexi	Dehalococcoides mccartyi 195 AAW40172
F_beta	Candidatus_Curtissbacteria_bacterium_GW2011_GWC2_41_21_KKS02109.1	Bacteria	Deferribacteres	Denitrovibrio acetiphilus
F_beta	Candidatus_Howlettbacteria_bacterium_CG23_combo_of_CG06-09_8_20_14_all_37_9_PIP30536.1	Bacteria	proteobacteria	Desulfovibrio vulgaris str. Hildenborough
F_beta	candidate_division_CPR2_bacterium_GW2011_GWC2_39_10_KKQ95280	Bacteria	Fusobacteria	Fusobacterium nucleatum
F_beta	candidate_division_CPR2_bacterium_GW2011_GWC2_39_35_KKR18687	Eukarya	Rhodphyta	Galdieria sulphuraria
F_beta	Candidatus_Peregrinibacteria_bacterium_GW2011_GWF2_43_17_KKT02618.1	Bacteria	proteobacteria	Geobacter sulfurreducens
F_beta	Opitutus_terrae_WP_012373701	Bacteria	Cyanobacteria	Gloeobacter violaceus PCC 7421
F_beta	Methylacidiphilum_infernorum_WP_012464757	Eukarya	Cryptophyta	Guillardia theta
F_beta	Chthoniobacter_flavus_WP_006981811	Bacteria	Fibrobacteres/Acidobacteria	Holophaga foetida
F_beta	Candidatus_Roizmanbacteria_bacterium_GW2011_GWB1_40_7_KKR71665.1	Eukarya	Opisthokonta	Homo sapiens
F_beta	Candidatus_Gottesmanbacteria_bacterium_GW2011_GWA2_43_14_KKS98507.1	Bacteria	Bacteroidetes/Chlorobi group	Ignavibacterium album
F_beta	Candidatus_Gottesmanbacteria_bacterium_GW2011_GWA2_42_18_KKS46809.1	Bacteria	Fusobacteria	Ilyobacter polytropus
F_beta	Candidatus_Roizmanbacteria_bacterium_RIFOXYA2_FULL_38_14_OGK62390.1	Bacteria	Thermotogae	Kosmotoga olearia
F_beta	Candidatus_Roizmanbacteria_bacterium_RIFCSPHIGHO2_02_FULL_38_11_OGK23342.1	Bacteria	Lentisphaerae	Lentisphaerae bacterium
F_beta	Candidatus_Roizmanbacteria_bacterium_CG_4_9_14_3_um_filter_36_11_PJA53485.1	Bacteria	spirochaetes	Leptospira interrogans serovar Lai
F_beta	Candidatus_Liptonbacteria_bacterium_CG11_big_fil_rev_8_21_14_0_20_35_14_PIR04861.1	Bacteria	proteobacteria	Magnetococcus marinus
F_beta	Candidatus_Peribacteria_bacterium_RIFCSPLOWO2_12_FULL_55_15_OGJ71667.1	Bacteria	Bacteroidetes/Chlorobi group	Melioribacter roseus P3M-2
F_beta	Chloracidobacterium_thermophilum_WP_041569105	Bacteria	Thermotogae	Mesoaciditoga lauensis
F_beta	Candidatus_Solibacter_usitatus_WP_011682251	Bacteria	Chlamydiae/Verrucomicrobia group	Methylacidiphilum infernorum
F_beta	Acidobacterium_capsulatum_WP_015896187	Bacteria	Nitrospinae/Tectomicrobia group	Nitrospina gracilis
F_beta	Candidatus_Aminicenantes_bacterium_RBG_19FT_COMBO_65_30_OGD29385.1	Bacteria	Nitrospinae/Tectomicrobia group	Nitrospinae bacterium CG11 big fil rev 8 21 14 0 20 45 15
F_beta	Candidatus_Aminicenantes_bacterium_RBG_16_63_16_OGD19777.1	Bacteria	Nitrospinae/Tectomicrobia group	Nitrospinae bacterium CG11 big fil rev 8 21 14 0 20 56 8
F_beta	Candidatus_Fischerbacteria_bacterium_RBG_13_37_8_OGF66644.1	Bacteria	Nitrospinae/Tectomicrobia group	Nitrospinae bacterium RIFCSPHIGHO2 02 39 11
F_beta	Candidatus_Handelsmanbacteria_bacterium_RIFCSPLOWO2_12_FULL_64_10_OGG44374.1	Bacteria	Omnitrophica	Omnitrophica bacterium RIFCSPLOWO2
F_beta	Candidatus_Latescibacteria_bacterium_4484_107_OPX23678	Bacteria	Chlamydiae/Verrucomicrobia group	Opitutus terrae
F_beta	Kosmotoga_olearia_WP_041288787	Eukarya	Viridiplantae	Oryza sativa japonica
F_beta	Mesoaciditoga_lauensis_WP_036221450	Eukarya	Alveolates	Oxytricha trifallax
F_beta	Thermotoga_maritima_MSB8_AAD36677	Bacteria	proteobacteria	Paracoccus denitrificans
F_beta	Petrotoga_mobilis_WP_012208572	Eukarya	Alveolates	Paramecium tetraurelia strain
F_beta	Candidatus_Marinimicrobia_bacterium_CG_4_10_14_0_2_um_filter_48_9_PIZ69983.1	Bacteria	Thermotogae	Petrotoga mobilis
F_beta	Candidatus_Yanofskybacteria_bacterium_RIFCSPLOWO2_01_FULL_41_67_OGN22880.1	Eukarya	Viridiplantae	Physcomitrella patens subsp. patens
F_beta	Candidatus_Yanofskybacteria_bacterium_GW2011_GWA2_44_9_KKT82173.1	Eukarya	Viridiplantae	Populus trichocarpa
F_beta	Candidatus_Taylorbacteria_bacterium_RIFCSPLOWO2_02_FULL_43_11_OHA37218.1	Eukarya	Rhodphyta	Porphyra umbilicalis
F_beta	Candidatus_Margulisbacteria_bacterium_GWF2_38_17_OGI02774.1	Bacteria	Bacteroidetes/Chlorobi group	Prosthecochloris aestuarii
F_beta	Leptospira_interrogans_serovar_Lai_str._56601_AAN49975	Bacteria	proteobacteria	Rhodospirillum rubrum
F_beta	candidate_division_WWE3_bacterium_CG_4_8_14_3_um_filter_42_11_PJC69282.1	Eukarya	Opisthokonta	Schistosoma mansoni
F_beta	Candidatus_Pacebacteria_bacterium_GW2011_GWA1_46_10_KKU18703.1	Eukarya	Viridiplantae	Spinacia oleracea
F_beta	Candidatus_Pacebacteria_bacterium_CG10_big_fil_rev_8_21_14_0_10_44_54_PIR60621.1	Bacteria	Fusobacteria	Streptobacillus moniliformis
F_beta	Candidatus_Pacebacteria_bacterium_CG_4_9_14_0_2_um_filter_34_50_PJC43848.1	Eukarya	Opisthokonta	Strongylocentrotus purpuratus
F_beta	Candidatus_Pacebacteria_bacterium_GW2011_GWF2_38_9_KKQ88510.1	Eukarya	Alveolates	Stylonychia lemnae
F_beta	Candidatus_Chisholmbacteria_bacterium_RIFCSPLOWO2_01_FULL_50_28_OGY20239.1	Bacteria	Cyanobacteria	Synechococcus elongatus
F_beta	Candidatus_Chisholmbacteria_bacterium_RIFCSPHIGHO2_01_FULL_48_12_OGY16910.1	Eukarya	Alveolates	Tetrahymena thermophila
F_beta	Candidatus_Beckwithbacteria_bacterium_RIFCSPLOWO2_02_FULL_47_23_OGD61452.1	Eukarya	stramenopiles	Thalassiosira pseudonana
F_beta	Candidatus_Beckwithbacteria_bacterium_CG_4_10_14_0_2_um_filter_47_25_PJA22939.1	Bacteria	unclassified Bacteria	Thermobaculum terrenum ATCC BAA-798
F_beta	Candidatus_Beckwithbacteria_bacterium_GW2011_GWA2_47_25_KKU71570.1	Bacteria	Thermodesulfobacteria	Thermodesulfatator indicus
F_beta	Candidatus_Beckwithbacteria_bacterium_CG23_combo_of_CG06-09_8_20_14_all_34_8_PIP53563.1	Bacteria	Thermodesulfobacteria	Thermodesulfobacterium commune
F_beta	Dehalococcoides_mccartyi_195_AAW40172	Bacteria	Thermodesulfobacteria	Thermodesulfobacterium geofontis
F_beta	Candidatus_Cloacimonetes_bacterium_4572_55_OQY26980.1	Bacteria	Aquificae	Thermosulfidibacter takaii
F_beta	Lentisphaerae_bacterium_RIFOXYC12_FULL_60_16_OGV65892	Bacteria	Cyanobacteria	Thermosynechococcus elongatus
F_beta	Candidatus_Woesebacteria_bacterium_RIFCSPLOWO2_01_FULL_39_23_OGM62783.1	Bacteria	Thermotogae	Thermotoga maritima

F_beta	Candidatus_Woesebacteria_bacterium_GW2011_GWB1_38_8_KKQ86233.1	Eukarya	Viridiplantae	Volvox carteri f. nagariensis
F_beta	Acetothermia_bacterium_64_32_KUK26690	Eukarya	Viridiplantae	Zea mays
