# Supplementary material for: Functional and structural characterization of F1 ‐ATPase with common ancestral core domains in stator ring
Source: Protein Sci. 2025 Oct 23;34(11):e70345. doi: 10.1002/pro.70345 (PMC12550136; doi:10.1002/pro.70345)
Supplement: Supplementary file 12 — Data S12: Sequence_T3SS_FliI. [file PRO-34-e70345-s015.pdf]

Subunit	Sequence Names	Domain	Phylum or Kingdom	Species
T3SS_FliI	Acidimicrobium_ferrooxidans_DSM_10331_ACU53109	Bacteria	Actinobacteria	Acidimicrobium ferrooxidans
T3SS_FliI	Acidobacterium_capsulatum_ATCC_51196_ACO34069	Bacteria	Fibrobacteres/Acidobacteria	Acidobacterium capsulatum
T3SS_FliI	Azotobacter_vinelandii_DJ_ACO78607	Bacteria	proteobacteria	Azotobacter vinelandii
T3SS_FliI	Bacillus_pseudofirmus_OF4_ADC48105.1	Bacteria	Firmicutes	Bacillus pseudofirmus
T3SS_FliI	Bacillus_pseudofirmus_OLO40894.1	Bacteria	Firmicutes	Bacillus pseudofirmus
T3SS_FliI	Bacillus_subtilis_subsp._subtilis_str._168_CAA39523	Bacteria	Firmicutes	Bacillus subtilis subsp. subtilis
T3SS_FliI	Borrelia_burgdorferi_B31_AAC66660	Bacteria	spirochaetes	Borrelia burgdorferi
T3SS_FliI	Burkholderia_mallei_ATCC_23344_AAU45957	Bacteria	proteobacteria	Burkholderia mallei
T3SS_FliI	Calditerrivibrio_nitroreducens_DSM_19672_ADR19689	Bacteria	Deferribacteres	Calditerrivibrio nitroreducens
T3SS_FliI	Caldithrix_abyssi_DSM_13497_EHO42246	Bacteria	Calditrichaeota	Caldithrix abyssi
T3SS_FliI	candidate_division_KSB1_bacterium_RBG_16_48_16_OGB99053	Bacteria	unclassified Bacteria	candidate division KSB1 bacterium RBG 16 48 16
T3SS_FliI	candidate_division_WOR-1_bacterium_RIFCSPHIGHO2_01_FULL_53_15_OGB90243.1	Bacteria	unclassified Bacteria	candidate division WOR-1 bacterium RIFCSPHIGHO2 01 FULL 53 15
T3SS_FliI	candidate_division_WOR-1_bacterium_RIFOXYA12_FULL_43_27_OGC05139.1	Bacteria	unclassified Bacteria	candidate division WOR-1 bacterium RIFOXYA12 FULL 43 27
T3SS_FliI	candidate_division_WOR-1_bacterium_RIFOXYA12_FULL_52_29_OGC17486.1	Bacteria	unclassified Bacteria	candidate division WOR-1 bacterium RIFOXYA12 FULL 52 29
T3SS_FliI	candidate_division_WOR-1_bacterium_RIFOXYA2_FULL_36_21_OGC06719.1	Bacteria	unclassified Bacteria	candidate division WOR-1 bacterium RIFOXYA2 FULL 36 21
T3SS_FliI	candidate_division_WOR-1_bacterium_RIFOXYC2_FULL_41_25_OGC33228.1	Bacteria	unclassified Bacteria	candidate division WOR-1 bacterium RIFOXYC2 FULL 41 25
T3SS_FliI	candidate_division_Zixibacteria_bacterium_CG_4_9_14_3_um_filter_46_8_PJA26641.1	Bacteria	unclassified Bacteria	candidate division Zixibacteria bacterium CG 4 9 14 3 um filter 46 8
T3SS_FliI	Candidatus_Blackallbacteria_bacterium_CG13_big_fil_rev_8_21_14_2_50_49_14_PIW49092.1	Bacteria	unclassified Bacteria	Candidatus Blackallbacteria bacterium
T3SS_FliI	Candidatus_Desantisbacteria_bacterium_CG_4_8_14_3_um_filter_40_12_PIX17104.1	Bacteria	unclassified Bacteria	Candidatus Desantisbacteria bacterium
T3SS_FliI	Candidatus_Gastranaerophilales_bacterium_HUM_21_DAB23645	Bacteria	unclassified Bacteria	Candidatus Gastranaerophilales bacterium HUM 21
T3SS_FliI	Candidatus_Gastranaerophilales_bacterium_HUM_5_DAA90817	Bacteria	unclassified Bacteria	Candidatus Gastranaerophilales bacterium HUM 5
T3SS_FliI	Candidatus_Gastranaerophilales_bacterium_HUM_5_DAA91232	Bacteria	unclassified Bacteria	Candidatus Gastranaerophilales bacterium HUM 5
T3SS_FliI	Candidatus_Gastranaerophilales_bacterium_HUM_6_DAA90030	Bacteria	unclassified Bacteria	Candidatus Gastranaerophilales bacterium HUM 6
T3SS_FliI	Candidatus_Gastranaerophilales_bacterium_HUM_9_DAA97120	Bacteria	unclassified Bacteria	Candidatus Gastranaerophilales bacterium HUM 9
T3SS_FliI	Candidatus_Goldbacteria_bacterium_HGW-Goldbacteria-1_PKL91641.1	Bacteria	unclassified Bacteria	Candidatus Goldbacteria bacterium HGW-Goldbacteria-1
T3SS_FliI	Candidatus_Handelsmanbacteria_bacterium_RIFCSPLOWO2_12_FULL_64_10_OGG56824.1	Bacteria	unclassified Bacteria	Candidatus Handelsmanbacteria bacterium RIFCSPLOWO2 12 FULL 64 10
T3SS_FliI	Candidatus_Hydrogenedentes_bacterium_CG1_02_42_14_OIO28214	Bacteria	unclassified Bacteria	Candidatus Hydrogenedentes bacterium CG1 02 42 14
T3SS_FliI	Candidatus_Kryptobacter_tengchongensis_CUU09675	Bacteria	Candidatus Kryptonia	Candidatus Kryptobacter tengchongensis
T3SS_FliI	Candidatus_Kryptonium_thompsoni_CUS76824	Bacteria	Candidatus Kryptonia	Candidatus Kryptonium thompsoni
T3SS_FliI	Candidatus_Lambdaproteobacteria_bacterium_RIFOXYC1_FULL_56_13_OGG99800.1	Bacteria	proteobacteria	Candidatus Lambdaproteobacteria bacterium RIFOXYC1 FULL 56 13
T3SS_FliI	Candidatus_Lambdaproteobacteria_bacterium_RIFOXYD2_FULL_50_16_OGG94765.1	Bacteria	proteobacteria	Candidatus Lambdaproteobacteria bacterium RIFOXYD2 FULL 50 16
T3SS_FliI	Candidatus_Latescibacteria_bacterium_4484_7_OQX84930	Bacteria	Candidatus Latescibacteria	Candidatus Latescibacteria bacterium 4484 7
T3SS_FliI	Candidatus_Lindowbacteria_bacterium_RIFCSPLOWO2_12_FULL_62_27_OGH63155.1	Bacteria	unclassified Bacteria	Candidatus Lindowbacteria bacterium RIFCSPLOWO2
T3SS_FliI	Candidatus_Margulisbacteria_bacterium_GWF2_35_9_OGI08237.1	Bacteria	unclassified Bacteria	Candidatus Margulisbacteria bacterium GWF2 35 9
T3SS_FliI	Candidatus_Margulisbacteria_bacterium_GWF2_38_17_OGI05189.1	Bacteria	unclassified Bacteria	Candidatus Margulisbacteria bacterium GWF2 38 17
T3SS_FliI	Candidatus_Melainabacteria_bacterium_GWA2_34_9_OGH96657.1	Bacteria	unclassified Bacteria	Candidatus Melainabacteria bacterium GWA2
T3SS_FliI	Candidatus_Melainabacteria_bacterium_MEL.A1_AOR39057.1	Bacteria	unclassified Bacteria	Candidatus Melainabacteria bacterium MEL.A1
T3SS_FliI	Candidatus_Melainabacteria_bacterium_RIFCSPHIGHO2_02_FULL_34_12_OGI18670.1	Bacteria	unclassified Bacteria	Candidatus Melainabacteria bacterium RIFCSPHIGHO2 02 FULL 34 12
T3SS_FliI	Candidatus_Melainabacteria_bacterium_RIFCSPLOWO2_12_FULL_35_11_OGI08658.1	Bacteria	unclassified Bacteria	Candidatus Melainabacteria bacterium RIFCSPLOWO2 12 FULL 35 11
T3SS_FliI	Candidatus_Melainabacteria_bacterium_RIFOXYA2_FULL_32_9_OGI23037.1	Bacteria	unclassified Bacteria	Candidatus Melainabacteria bacterium RIFOXYA2 FULL 32 9
T3SS_FliI	Candidatus_Nitrospira_defluvii_CBK42004	Bacteria	Nitrospirae	Candidatus Nitrospira defluvii
T3SS_FliI	Candidatus_Riflebacteria_bacterium_GWC2_50_8_OGK05596	Bacteria	unclassified Bacteria	Candidatus Riflebacteria bacterium GWC2
T3SS_FliI	Candidatus_Riflebacteria_bacterium_HGW-Riflebacteria-1_PKL45707.1	Bacteria	unclassified Bacteria	Candidatus Riflebacteria bacterium HGW-Riflebacteria-1
T3SS_FliI	Candidatus_Riflebacteria_bacterium_HGW-Riflebacteria-2_PKL49781.1	Bacteria	unclassified Bacteria	Candidatus Riflebacteria bacterium HGW-Riflebacteria-2
T3SS_FliI	Candidatus_Rokubacteria_bacterium_13_1_40CM_68_15_OLB94027.1	Bacteria	unclassified Bacteria	Candidatus Rokubacteria bacterium 13 1 40CM
T3SS_FliI	Candidatus_Saganbacteria_bacterium_CG08_land_8_20_14_0_20_45_16_PIS29985.1	Bacteria	unclassified Bacteria	Candidatus Saganbacteria bacterium
T3SS_FliI	Candidatus_Solibacter_usitatus_Ellin6076_ABJ81355	Bacteria	Fibrobacteres/Acidobacteria	Candidatus Solibacter usitatus
T3SS_FliI	Candidatus_Wallbacteria_bacterium_GWC2_49_35_OGM07894	Bacteria	unclassified Bacteria	Candidatus Wallbacteria bacterium GWC2

T3SS_FliI	Candidatus_Wallbacteria_bacterium_HGW-Wallbacteria-1_PKK91562.1	Bacteria	unclassified Bacteria	Candidatus Wallbacteria bacterium HGW-Wallbacteria-1
T3SS_FliI	Chitinispirillum_alkaliphilum_KMQ51162	Bacteria	Fibrobacteres/Acidobacteria	Chitinispirillum alkaliphilum
T3SS_FliI	Chitinivibrio_alkaliphilus_ACht1_ERP31686	Bacteria	Fibrobacteres/Acidobacteria	Chitinivibrio alkaliphilus
T3SS_FliI	Chlamydia_pneumoniae_WP_010883345	Bacteria	Chlamydiae/Verrucomicrobia group	Chlamydophila pneumoniae
T3SS_FliI	Chlamydia_trachomatis_D/UW-3/CX_AAC68264	Bacteria	Chlamydiae/Verrucomicrobia group	Chlamydia trachomatis
T3SS_FliI	Chloracidobacterium_thermophilum_B_AEP13655	Bacteria	Fibrobacteres/Acidobacteria	Chloracidobacterium thermophilum
T3SS_FliI	Chrysiogenes_arsenatis_WP_034764826	Bacteria	Chrysiogenetes	Chrysiogenes arsenatis
T3SS_FliI	Clostridium_acetobutylicum_ATCC_824_AAK80117	Bacteria	Firmicutes	Clostridium acetobutylicum
T3SS_FliI	Deferribacter_desulfuricans_SSM1_BAI80019	Bacteria	Deferribacteres	Deferribacter desulfuricans
T3SS_FliI	Denitrovibrio_acetiphilus_DSM_12809_ADD69073	Bacteria	Deferribacteres	Denitrovibrio acetiphilus
T3SS_FliI	Desulfovibrio_vulgaris_str._Hildenborough_AAS94344	Bacteria	proteobacteria	Desulfovibrio vulgaris str. Hildenborough
T3SS_FliI	Desulfovibrio_vulgaris_str._Hildenborough_AAS94793	Bacteria	proteobacteria	Desulfovibrio vulgaris str. Hildenborough
T3SS_FliI	Desulfurispirillum_indicum_S5_ADU66930	Bacteria	Chrysiogenetes	Desulfurispirillum indicum
T3SS_FliI	Desulfurobacterium_thermolithotrophum_DSM_11699_ADY73870	Bacteria	Aquificae	Desulfurobacterium thermolithotrophum
T3SS_FliI	Escherichia_coli_str._K-12_substr._MG1655_AAC75008	Bacteria	proteobacteria	Escherichia coli
T3SS_FliI	Gemmata_obscuriglobus_WP_029600690	Bacteria	Planctomycetes	Gemmata obscuriglobus
T3SS_FliI	Gemmatimonas_aurantiaca_T-27_BAH37370	Bacteria	Gemmtimonadetes	Gemmatimonas aurantiaca T-27
T3SS_FliI	Gemmatimonas_phototrophica_WP_043580514	Bacteria	Gemmtimonadetes	Gemmatimonas phototrophica
T3SS_FliI	Holophaga_foetida_WP_005036653	Bacteria	Fibrobacteres/Acidobacteria	Holophaga foetida
T3SS_FliI	Ignavibacterium_album_JCM_16511_AFH50243	Bacteria	Bacteroidetes/Chlorobi group	Ignavibacterium album
T3SS_FliI	Legionella_pneumophila_subsp._pneumophila_str._Philadelphia_1_AAU27836	Bacteria	proteobacteria	Legionella pneumophila subsp. Pneumophila
T3SS_FliI	Leptospirillum_ferrooxidans_C2-3_BAM05995	Bacteria	Nitrospirae	Leptospirillum ferrooxidans
T3SS_FliI	Limnochorda_pilosa_BAS27500	Bacteria	Firmicutes	Limnochorda pilosa
T3SS_FliI	Magnetococcus_marinus_MC-1_ABK42799	Bacteria	proteobacteria	Magnetococcus marinus
T3SS_FliI	Mariprofundus_aestuarium_ATX78581.1	Bacteria	proteobacteria	Mariprofundus aestuarium
T3SS_FliI	Melioribacter_roseus_P3M-2_AFN75362	Bacteria	Bacteroidetes/Chlorobi group	Melioribacter roseus P3M-2
T3SS_FliI	Methylobacillus_flagellatus_KT_ABE50242	Bacteria	proteobacteria	Methylobacillus flagellatus KT
T3SS_FliI	Myxococcus_xanthus_DK_1622_ABF91762	Bacteria	proteobacteria	Myxococcus xanthus
T3SS_FliI	Nitrosomonas_europaea_WP_041357274	Bacteria	proteobacteria	Nitrosomonas europaea
T3SS_FliI	Nitrospina_gracilis_3/211_CCQ90547	Bacteria	Nitrospinae/Tectomicrobia group	Nitrospina gracilis
T3SS_FliI	Nitrospinae_bacterium_CG11_big_fil_rev_8_21_14_0_20_45_15_PIR00522.1	Bacteria	Nitrospinae/Tectomicrobia group	Nitrospinae bacterium CG11 big fil rev 8 21 14 0 20 45 17
T3SS_FliI	Opitutus_terrae_PB90-1_ACB73709	Bacteria	Chlamydiae/Verrucomicrobia group	Opitutus terrae
T3SS_FliI	Petrotoga_mobilis_SJ95_ABX32080	Bacteria	Thermotogae	Petrotoga mobilis
T3SS_FliI	Pseudomonas_putida_GB-1_ABY99817	Bacteria	proteobacteria	Pseudomonas putida GB-1
T3SS_FliI	Shewanella_oneidensis_MR-1_AAN56224	Bacteria	proteobacteria	Shewanella oneidensis MR-1
T3SS_FliI	Simkania_negevensis_WP_013944344	Bacteria	Chlamydiae/Verrucomicrobia group	Simkania negevensis
T3SS_FliI	Sphingomonas_paucimobilis_NBRC_13935_GAN13617	Bacteria	proteobacteria	Sphingomonas paucimobilis NBRC 13935
T3SS_FliI	Spirochaeta_thermophila_DSM_6192_ADN01860.1	Bacteria	spirochaetes	Spirochaeta thermophila
T3SS_FliI	Sulfuricella_denitrificans_skB26_BAN35059	Bacteria	proteobacteria	Sulfuricella denitrificans
T3SS_FliI	Thermanaerovibrio_acidaminovorans_DSM_6589_ACZ19565	Bacteria	Synergistetes	Thermanaerovibrio acidaminovorans
T3SS_FliI	Thermodesulfatator_indicus_DSM_15286_AEH45670	Bacteria	Thermodesulfobacteria	Thermodesulfatator indicus
T3SS_FliI	Thermodesulfobacterium_geofontis_OPF15_AEH22179	Bacteria	Thermodesulfobacteria	Thermodesulfobacterium geofontis
T3SS_FliI	Thermomicrobium_roseum_DSM_5159_ACM07031	Bacteria	Chloroflexi	Thermomicrobium roseum DSM 5159
T3SS_FliI	Thermotoga_maritima_MSB8_AAD35310	Bacteria	Thermotogae	Thermotoga maritima
T3SS_FliI	Thiobacillus_denitrificans_ATCC_25259_AAZ97556	Bacteria	proteobacteria	Thiobacillus denitrificans ATCC 25259
T3SS_FliI	Vibrio_cholerae_O1_biovar_El_Tor_str._N16961_AAF95275	Bacteria	proteobacteria	Vibrio cholerae O1 biovar El Tor
