# Supplementary material for: Functional and structural characterization of F1 ‐ATPase with common ancestral core domains in stator ring
Source: Protein Sci. 2025 Oct 23;34(11):e70345. doi: 10.1002/pro.70345 (PMC12550136; doi:10.1002/pro.70345)
Supplement: Supplementary file 13 — Data S13: Sequence_V‐type_A. [file PRO-34-e70345-s003.pdf]

Subunit	Sequence Names	Domain	Phylum or Kingdom	Species
V_A	Thermus_thermophilus_HB27_AAS81251	Bacteria	Tenericutes	Acholeplasma laidlawii
V_A	Deinococcus_radiodurans_R1_AAF10278	Bacteria	Tenericutes	Acholeplasma laidlawii
V_A	Meiothermus_ruber_DSM_1279_ADD27726	Archaea	Euryarchaeota	Aciduliprofundum boonei
V_A	Candidatus_Edwardsbacteria_bacterium_RifOxyC12_full_54_24_OGF04251.1	Bacteria	Synergistetes	Aminobacterium colombiense
V_A	Candidatus_Micrarchaeota_archaeon_CG_4_10_14_0_2_um_filter_60_11_PIZ91073.1	Archaea	Euryarchaeota	Archaeoglobus fulgidus
V_A	Aciduliprofundum_boonei_T469_ADD09070	Archaea	Euryarchaeota	Archaeoglobus veneficus
V_A	Pyrococcus_horikoshii_OT3_3M4Y_A	Eukarya	Opisthokonta	Botrytis cinerea
V_A	Thermococcus_kodakarensis_KOD1_BAD85791	Bacteria	Caldiserica	Caldisericum exile
V_A	Methanocaldococcus_jannaschii_DSM_2661_AAB98200	Bacteria	Caldiserica	Caldisericum sp.
V_A	Methanococcus_maripaludis_S2_CAF30600	Bacteria	unclassified Bacteria	candidate division Hyd24-12 bacterium Dam 1
V_A	Methanopyrus_kandleri_AV19_AAM02230	Bacteria	unclassified Bacteria	candidate division TA06 bacterium 32 111
V_A	Methanobacterium_formicicum_DSM_3637_EKF85769	Bacteria	unclassified Bacteria	candidate division TA06 bacterium DG 26
V_A	Methanobrevibacter_smithii_ATCC_35061_A5UKB2	Bacteria	unclassified Bacteria	candidate division TA06 bacterium SM23 40
V_A	Candidatus_Altiarchaeales_archaeon_ex4484_2_OYT54103	Bacteria	unclassified Bacteria	candidate division WOR-1 bacterium RIFOXYA12 FULL 43 27
V_A	Candidatus_Altiarchaeales_archaeon_IMC4_ODS42834	Bacteria	unclassified Bacteria	candidate division WOR-1 bacterium RIFOXYC2 FULL 41 25
V_A	Candidatus_Methanohalarchaeum_thermophilum_OKY78117	Bacteria	unclassified Bacteria	candidate division WOR 3 bacterium JGI Cruoil
V_A	Methanonatronarchaeum_thermophilum_OUJ19125	Bacteria	unclassified Bacteria	candidate division Zixibacteria bacterium SM23 81
V_A	Archaeoglobus_fulgidus_DSM_4304_AAB90074	Bacteria	Acetothermia	Candidatus Acetothermus autotrophicum
V_A	Archaeoglobus_veneficus_SNP6_AEA46338	Bacteria	unclassified Bacteria	Candidatus Aegiribacteria bacterium
V_A	Ferroglobus_placidus_DSM_10642_ADC66428	Archaea	Aenigmarchaeota	Candidatus Aenigmarchaeota archaeon
V_A	Candidatus_Methanoperedens_nitroreducens_KCZ71341	Archaea	Euryarchaeota	Candidatus Altiarchaeales archaeon ex4484
V_A	Methanohalophilus_mahii_DSM_5219_ADE36942	Archaea	Euryarchaeota	Candidatus Altiarchaeales archaeon IMC4
V_A	Methanosarcina_barkeri_WP_048119904	Bacteria	unclassified Bacteria	Candidatus Andersenbacteria bacterium CG10
V_A	Methanocella_paludicola_SANAE_BAI60411	Bacteria	unclassified Bacteria	Candidatus Andersenbacteria bacterium RIFCSPHIGHO2
V_A	Marine_Group_III_euryarchaeote_CG-Bathy1_OIR19065	Bacteria	Atribacteria	Candidatus Atribacteria bacterium 4572 76
V_A	Marine_Group_III_euryarchaeote_CG-Epi1_OIR20625	Bacteria	Atribacteria	Candidatus Atribacteria bacterium CG2 30 33 13
V_A	Candidatus_Diapherotrites_archaeon_CG08_land_8_20_14_0_20_34_12_PIU21675.1	Bacteria	Atribacteria	Candidatus Atribacteria bacterium CG2 30 33 13
V_A	Candidatus_Methanomethylophilus_alvus_Mx1201_AGI84768	Bacteria	Atribacteria	Candidatus Atribacteria bacterium HGW-Atribacteria-1
V_A	Marine_Group_II_euryarchaeote_MED-G33_PDH27846.1	Bacteria	Atribacteria	Candidatus Atribacteria bacterium RBG 19FT COMBO 35 14
V_A	uncultured_Candidatus_Thalassoarchaea_euryarchaeote_ANV81271	Bacteria	Atribacteria	Candidatus Atribacteria bacterium RBG 19FT COMBO 35 14
V_A	Marine_Group_II_euryarchaeote_MED-G34_PDH26413.1	Archaea	Aigarchaeota	Candidatus Caldiarchaeum subterraneum
V_A	Marine_Group_II_euryarchaeote_MED-G37_PDH22019.1	Bacteria	unclassified Bacteria	Candidatus Desantisbacteria bacterium
V_A	Marine_Group_II_euryarchaeote_MED-G36_PDH24508.1	Archaea	Diapherotrites	Candidatus Diapherotrites archaeon
V_A	Marine_Group_II_euryarchaeote_MED-G38_PDH23634.1	Bacteria	unclassified Bacteria	Candidatus Edwardsbacteria bacterium RifOxyC12 full 54 24
V_A	Candidatus_Woesearchaeota_archaeon_CG10_big_fil_rev_8_21_14_0_10_45_16_PIN74292.1	Bacteria	unclassified Bacteria	Candidatus Gribaldobacteria bacterium CG 4
V_A	Candidatus_Aenigmarchaeota_archaeon_ex4484_52_OYT34499.1	Bacteria	unclassified Bacteria	Candidatus Gribaldobacteria bacterium CG10
V_A	Candidatus_Micrarchaeota_archaeon_CG10_big_fil_rev_8_21_14_0_10_45_29_PIT84214.1	Archaea	Nanohaloarchaeota	Candidatus Haloredivivus sp. G17
V_A	Candidatus_Haloredivivus_sp._G17_EHK02280	Archaea	Heimdallarchaeota	Candidatus Heimdallarchaeota archaeon AB 125
V_A	Candidatus_Nanosalina_sp._J07AB43_EGQ42837	Bacteria	unclassified Bacteria	Candidatus Jacksonbacteria bacterium RIFOXYD2 FULL 43 21
V_A	Nanohaloarchaea_archaeon_SG9_AOV95276	Archaea	Korarchaeota	Candidatus Korarchaeum cryptofilum OPF8
V_A	Halobacterium_salinarum_R1_B0R755	Bacteria	Latescibacteria	Candidatus Latescibacteria bacterium 4484 107
V_A	halophilic_archaeon_DL31_AEN06657	Bacteria	Candidatus Latescibacteria	Candidatus Latescibacteria bacterium 4484 7
V_A	Natrialba_magadii_ATCC_43099_ADD04950	Bacteria	Fibrobacteres/Acidobacteria	Candidatus Marinimicrobia bacterium CG08
V_A	Candidatus_Acetothermus_autotrophicum_BAL59642	Archaea	Euryarchaeota	Candidatus Methanohalarchaeum thermophilum
V_A	candidate_division_TA06_bacterium_32_111_KUK51433	Archaea	Euryarchaeota	Candidatus Methanomethylophilus alvus Mx1201
V_A	candidate_division_TA06_bacterium_DG_26_KPJ49875	Archaea	Euryarchaeota	Candidatus Methanoperedens nitroreducens
V_A	candidate_division_TA06_bacterium_SM23_40_KPK68481	Archaea	Micrarchaeota	Candidatus Micrarchaeota archaeon CG 4

V_A	candidate_division_Zixibacteria_bacterium_SM23_81_KPL18986	Archaea	Micrarchaeota	Candidatus Micrarchaeota archaeon CG10
V_A	candidate_division_WOR-3_bacterium_JGI_Cruoil_03_44_89_OYD15240	Archaea	Micrarchaeota	Candidatus Micrarchaeota archaeon Mia14
V_A	Candidatus_Stahlbacteria_bacterium_CG23_combo_of_CG06-09_8_20_14_all_40_9_PIP11804.1	Archaea	Micrarchaeota	Candidatus Micrarchaeum acidiphilum ARMAN-2
V_A	Candidatus_Atribacteria_bacterium_4572_76_OQY41157.1	Bacteria	unclassified Bacteria	Candidatus Moduliflexus flocculans (bacterium UASB14)
V_A	Candidatus_Atribacteria_bacterium_HGW-Atribacteria-1_PKP58334.1	Bacteria	unclassified Bacteria	Candidatus Moduliflexus flocculans (bacterium UASB14)
V_A	Candidatus_Atribacteria_bacterium_RBG_19FT_COMBO_35_14_OGD14190.1	Archaea	Nanohaloarchaeota	Candidatus Nanosalina sp. J07AB43
V_A	Candidatus_Atribacteria_bacterium_CG2_30_33_13_OIP75061.1	Bacteria	unclassified Bacteria	Candidatus Nealsonbacteria bacterium
V_A	Candidatus_Desantisbacteria_bacterium_CG_4_8_14_3_um_filter_40_12_PIX16275.1	Archaea	Thaumarchaeota	Candidatus Nitrosoarchaeum koreensis
V_A	Theionarchaea_archaeon_DG-70-1_KYK32459	Archaea	Thaumarchaeota	Candidatus Nitrosoarchaeum limnia
V_A	Theionarchaea_archaeon_DG-70_KYK38938	Archaea	Thaumarchaeota	Candidatus Nitrosopelagicus brevis
V_A	Aminobacterium_colombiense_DSM_12261_ADE56940	Archaea	Thaumarchaeota	Candidatus Nitrosopumilus salaria
V_A	Pyramidobacter_piscolens_W5455_EFB89867	Archaea	Thaumarchaeota	Candidatus Nitrososphaera evergladensis SR1
V_A	Synergistes_jonesii_KEJ92088	Archaea	Thaumarchaeota	Candidatus Nitrososphaera gargensis
V_A	Finegoldia_magna_ATCC_29328_BAG08492	Archaea	Thaumarchaeota	Candidatus Nitrosotalea devanaterra
V_A	Streptobacillus_moniliformis_DSM_12112_ACZ00741	Archaea	Thaumarchaeota	Candidatus Nitrosotenuis cloacae
V_A	candidate_division_Hyd24-12_bacterium_Dam_1_KZD19792	Archaea	Parvarchaeota	Candidatus Parvarchaeum acidiphilum ARMAN-4
V_A	Candidatus_Aegiribacteria_bacterium_MLS_C_OPL19697.1	Archaea	Parvarchaeota	Candidatus Parvarchaeum acidophilus ARMAN-5
V_A	Dictyoglomus_thermophilum_H-6-12_ACI19501	Bacteria	unclassified Bacteria	Candidatus Ratteibacteria bacterium CG15
V_A	Dictyoglomus_turgidum_DSM_6724_ACK42775	Bacteria	unclassified Bacteria	Candidatus Stahlbacteria bacterium
V_A	Caldisericum_exile_AZM16c01_BAL81284	Archaea	Thorarchaeota	Candidatus Thorarchaeota archaeon AB 25
V_A	Caldisericum_sp._CG2_30_36_11_OIP12904.1	Archaea	Thorarchaeota	Candidatus Thorarchaeota archaeon SMTZ1-45
V_A	candidate_division_WOR-1_bacterium_RIFOXYA12_FULL_43_27_OGC05192.1	Archaea	Thorarchaeota	Candidatus Thorarchaeota archaeon SMTZ1-83
V_A	candidate_division_WOR-1_bacterium_RIFOXYC2_FULL_41_25_OGC34879.1	Bacteria	unclassified Bacteria	Candidatus Vecturithrix granuli (bacterium UASB270)
V_A	Candidatus_Ratteibacteria_bacterium_CG15_BIG_FIL_POST_REV_8_21_14_020_41_12_PIW34082.1	Bacteria	unclassified Bacteria	Candidatus Vecturithrix granuli (bacterium UASB270)
V_A	Candidatus_Jacksonbacteria_bacterium_RIFOXYD2_FULL_43_21_OGY81761.1	Archaea	Woesearchaeota	Candidatus Woesearchaeota archaeon
V_A	Candidatus_Gribaldobacteria_bacterium_CG10_big_fil_rev_8_21_14_0_10_37_21_PIR90648.1	Bacteria	unclassified Bacteria	Candidatus Yonathbacteria bacterium
V_A	Candidatus_Gribaldobacteria_bacterium_CG_4_10_14_0_2_um_filter_41_16_PJA01426.1	Archaea	Thaumarchaeota	Cenarchaeum symbiosum A
V_A	Candidatus_Nealsonbacteria_bacterium_RIFOXYB1_FULL_40_15_OGZ27424.1	Bacteria	Deinococcus-Thermus	Deinococcus radiodurans
V_A	Candidatus_Andersenbacteria_bacterium_CG10_big_fil_rev_8_21_14_0_10_54_11_PIT97716.1	Archaea	Crenarchaeota	Desulfurococcus mucosus DSM 2163
V_A	Candidatus_Andersenbacteria_bacterium_RIFCSPHIGHO2_12_FULL_45_11b_OGY37232.1	Bacteria	Dictyoglomi	Dictyoglomus thermophilum
V_A	Candidatus_Micrarchaeota_archaeon_Mia14_ASI13681.1	Bacteria	Dictyoglomi	Dictyoglomus turgidum
V_A	Candidatus_Micrarchaeum_acidiphilum_ARMAN-2_EET90349	Eukarya	Amoebozoa	Entamoeba histolytica
V_A	Candidatus_Parvarchaeum_acidiphilum_ARMAN-4_EEZ93165	Bacteria	Firmicutes	Erysipelothrix rhusiopathiae str. Fujisawa
V_A	Candidatus_Parvarchaeum_acidophilus_ARMAN-5_EFD92604	Archaea	Euryarchaeota	Ferroglobus placidus
V_A	Ferroplasma_acidarmanus_fer1_AGO60916	Archaea	Euryarchaeota	Ferroplasma acidarmanus fer1
V_A	Candidatus_Nitrosotalea_devanaterra_CUR52766	Bacteria	Firmicutes	Finegoldia magna ATCC 29328
V_A	Methanocella_paludicola_SANAE_BAI62362	Bacteria	Firmicutes	Finegoldia magna ATCC 29328
V_A	Candidatus_Heimdallarchaeota_archaeon_AB_125_OLS31618	Archaea	Euryarchaeota	Halobacterium salinarum R1
V_A	Candidatus_Moduliflexus_flocculans_GAK54039.1	Archaea	unclassified archaea	halophilic archaeon DL31
V_A	Candidatus_Vecturithrix_granuli_GAK59872.1	Bacteria	Fibrobacteres/Acidobacteria	Holophaga foetida
V_A	Acholeplasma_laidlawii_PG-8A_ABX81583	Eukarya	Euglenozoa	Leishmania major
V_A	Candidatus_Thorarchaeota_archaeon_AB_25_OLS31424	Archaea	Euryarchaeota	Marine Group II euryarchaeote MED-G33
V_A	Candidatus_Thorarchaeota_archaeon_SMTZ1-45_KXH73384	Archaea	Euryarchaeota	Marine Group II euryarchaeote MED-G34
V_A	Candidatus_Thorarchaeota_archaeon_SMTZ1-83_KXH77548	Archaea	Euryarchaeota	Marine Group II euryarchaeote MED-G36
V_A	Candidatus_Caldiarchaeum_subterraneum_BAE03290	Archaea	Euryarchaeota	Marine Group II euryarchaeote MED-G37
V_A	Candidatus_Nitrosoarchaeum_koreensis_MY1_EGP94529	Archaea	Euryarchaeota	Marine Group II euryarchaeote MED-G38
V_A	Candidatus_Nitrosoarchaeum_limnia_BG20_EPA06812	Archaea	Euryarchaeota	Marine Group III euryarchaeote CG-Bathy1
V_A	Nitrosopumilus_maritimus_SCM1_A9A2R0	Archaea	Euryarchaeota	Marine Group III euryarchaeote CG-Epi1
V_A	Candidatus_Nitrosopumilus_salaria_BD31_EIJ66210.1	Bacteria	Deinococcus-Thermus	Meiothermus ruber

V_A	Candidatus_Nitrosopelagicus_brevis_AJA92738	Bacteria	Thermotogae	Mesoaciditoga lauensis
V_A	Cenarchaeum_symbiosum_A_A0RXK1	Archaea	Euryarchaeota	Methanobacterium formicicum DSM 3637
V_A	Candidatus_Nitrosotenuis_cloacae_AJZ75933	Archaea	Euryarchaeota	Methanobrevibacter smithii ATCC 35061
V_A	Thaumarchaeota_archaeon_N4_CDI06744	Archaea	Euryarchaeota	Methanocaldococcus jannaschii
V_A	Thaumarchaeota_archaeon_MY2_WP_042684208	Archaea	Euryarchaeota	Methanocella paludicola SANAE
V_A	Candidatus_Nitrososphaera_evergladensis_SR1_AIF85308	Archaea	Euryarchaeota	Methanocella paludicola SANAE
V_A	Nitrososphaera_viennensis_EN76_AIC16550	Archaea	Euryarchaeota	Methanococcus maripaludis S2
V_A	Candidatus_Nitrososphaera_gargensis_Ga9.2_AFU60106	Archaea	Euryarchaeota	Methanohalophilus mahii
V_A	Candidatus_Atribacteria_bacterium_CG2_30_33_13_OIP71946.1	Archaea	Euryarchaeota	Methanonatronarchaeum thermophilum
V_A	Candidatus_Atribacteria_bacterium_RBG_19FT_COMBO_35_14_OGD17393.1	Archaea	Euryarchaeota	Methanopyrus kandleri
V_A	Finegoldia_magna_ATCC_29328_BAG08584	Archaea	Euryarchaeota	Methanosarcina barkeri
V_A	Mesoaciditoga_lauensis_WP_036224517	Archaea	Nanohaloarchaeota	Nanohaloarchaea archaeon SG9
V_A	Candidatus_Latescibacteria_bacterium_4484_7_OQX86192	Archaea	Euryarchaeota	Natrialba magadii ATCC 43099
V_A	Candidatus_Marinimicrobia_bacterium_CG08_land_8_20_14_0_20_45_22_PIS28245.1	Archaea	Thaumarchaeota	Nitrosopumilus maritimus SCM1
V_A	Candidatus_Moduliflexus_flocculans_GAK54670.1	Archaea	Thaumarchaeota	Nitrososphaera viennensis EN76
V_A	Candidatus_Vecturithrix_granuli_GAK55007.1	Bacteria	Nitrospinae/Tectomicrobia group	Nitrospinae bacterium RIFCSPHIGHO2 02 39 11
V_A	Candidatus_Latescibacteria_bacterium_4484_107_OPX21681	Bacteria	Synergistetes	Pyramidobacter piscolens
V_A	Desulfurococcus_mucosus_DSM_2162_ADV64400	Archaea	Euryarchaeota	Pyrococcus horikoshii OT3
V_A	Thermosphaera_aggregans_DSM_11486_ADG90361	Archaea	Crenarchaeota	Staphylothermus marinus F1
V_A	Staphylothermus_marinus_F1_A3DNQ6	Bacteria	Fusobacteria	Streptobacillus moniliformis
V_A	Holophaga_foetida_WP_005035429	Eukarya	Euglenozoa	Strigomonas culicis
V_A	Candidatus_Yonathbacteria_bacterium_CG_4_10_14_0_8_um_filter_47_645_PIY57639.1	Bacteria	Synergistetes	Synergistes jonesii
V_A	Acholeplasma_laidlawii_PG-8A_ABX81768	Archaea	Thaumarchaeota	Thaumarchaeota archaeon MY2
V_A	Erysipelothrix_rhusiopathiae_str._Fujisawa_BAK32099	Archaea	Thaumarchaeota	Thaumarchaeota archaeon N4
V_A	Nitrospinae_bacterium_RIFCSPHIGHO2_02_39_11_OGV97041.1	Archaea	Euryarchaeota	Theionarchaea archaeon DG-70
V_A	Thermofilum_pendens_Hrk_5_A1RX21	Archaea	Euryarchaeota	Theionarchaea archaeon DG-70-1
V_A	Candidatus_Korarchaeum_cryptofilum_OPF8_ACB08046	Archaea	Euryarchaeota	Thermococcus kodakarensis KOD1
V_A	Entamoeba_histolytica_HM-1	Archaea	Crenarchaeota	Thermofilum pendens Hrk 5
V_A	Strigomonas_culicis_EPY20193	Archaea	Crenarchaeota	Thermosphaera aggregans
V_A	Leishmania_major_strain_Friedlin_XP_001686471.1	Bacteria	Deinococcus-Thermus	Thermus thermophilus HB28
V_A	Trypanosoma_brucei_AAX80929	Eukarya	Euglenozoa	Trypanosoma brucei
V_A	Botrytis_cinerea_BcDW1_EMR90066	Archaea	Euryarchaeota	uncultured Candidatus Thalassoarchaea euryarchaeote
