# Supplementary material for: Functional and structural characterization of F1 ‐ATPase with common ancestral core domains in stator ring
Source: Protein Sci. 2025 Oct 23;34(11):e70345. doi: 10.1002/pro.70345 (PMC12550136; doi:10.1002/pro.70345)
Supplement: Supplementary file 14 — Data S14: Sequence_V‐type_B. [file PRO-34-e70345-s001.pdf]

Subunit	Sequence Names	Domain	Phylum or Kingdom	Species
V_B	Thermus_thermophilus_HB27_AAS81250	Bacteria	Tenericutes	Acholeplasma laidlawii
V_B	Meiothermus_ruber_DSM_1279_ADD27727	Archaea	Crenarchaeota	Acidilobus saccharovorans
V_B	Deinococcus_radiodurans_R1_AAF10279	Archaea	Euryarchaeota	Aciduliprofundum boonei
V_B	Candidatus_Edwardsbacteria_bacterium_RifOxyC12_full_54_24_OGF04254.1	Archaea	Crenarchaeota	Aeropyrum pernix
V_B	Acholeplasma_laidlawii_PG-8A_ABX81582	Bacteria	Synergistetes	Aminobacterium colombiense
V_B	Aminobacterium_colombiense_DSM_12261_ADE56941	Bacteria	Caldiserica	Caldisericum exile
V_B	Thermanaerovibrio_acidaminovorans_DSM_6589_ACZ19092	Bacteria	Caldiserica	Caldisericum sp.
V_B	Synergistes_jonesii_KEJ92087	Archaea	Crenarchaeota	Caldisphaera lagunensis
V_B	Pyramidobacter_piscolens_W5455_EFB89868	Archaea	Crenarchaeota	Caldivirga maquilingensis
V_B	Candidatus_Atribacteria_bacterium_4572_76_OQY41156.1	Bacteria	unclassified Bacteria	candidate division TA06 bacterium 32 111
V_B	Candidatus_Atribacteria_bacterium_RBG_19FT_COMBO_35_14_OGD14189.1	Bacteria	unclassified Bacteria	candidate division TA06 bacterium DG 78
V_B	Candidatus_Atribacteria_bacterium_CG2_30_33_13_OIP75062.1	Bacteria	unclassified Bacteria	candidate division TA06 bacterium SM23 40
V_B	candidate_division_TA06_bacterium_32_111_KUK51434	Bacteria	unclassified Bacteria	candidate division WOR 3 bacterium
V_B	candidate_division_TA06_bacterium_SM23_40_KPK68482	Bacteria	unclassified Bacteria	candidate division WOR-1 bacterium RIFOXYA12 FULL 43 27
V_B	candidate_division_Zixibacteria_bacterium_SM23_81_KPL18985	Bacteria	unclassified Bacteria	candidate division WOR-1 bacterium RIFOXYC2 FULL 41 25
V_B	candidate_division_WOR-3_bacterium_JGI_Cruoil_03_44_89_OYD15239	Bacteria	unclassified Bacteria	candidate division WOR 3 bacterium JGI Cruoil
V_B	Candidatus_Stahlbacteria_bacterium_CG23_combo_of_CG06-09_8_20_14_all_40_9_PIP11803.1	Bacteria	unclassified Bacteria	candidate division WOR 3 bacterium JGI Cruoil 03 51 56
V_B	candidate_division_TA06_bacterium_DG_78_KPJ73186	Bacteria	unclassified Bacteria	candidate division Zixibacteria bacterium SM23 81
V_B	candidate_division_WOR_3_bacterium_SM23_42_KPK62704	Bacteria	Acetothermia	Candidatus Acetothermus autotrophicum
V_B	Candidatus_Ratteibacteria_bacterium_CG15_BIG_FIL_POST_REV_8_21_14_020_41_12_PIW34086.1	Archaea	Euryarchaeota	Candidatus Altiarchaeales archaeon ex4484
V_B	Candidatus_Ratteibacteria_bacterium_CG23_combo_of_CG06-09_8_20_14_all_48_7_PIP16688.1	Archaea	Euryarchaeota	Candidatus Altiarchaeales archaeon IMC4
V_B	candidate_division_WOR-3_bacterium_JGI_Cruoil_03_51_56_OYD16486	Bacteria	Atribacteria	Candidatus Atribacteria bacterium 4572 76
V_B	Finegoldia_magna_ATCC_29328_BAG08491	Bacteria	Atribacteria	Candidatus Atribacteria bacterium CG2 30 33 13
V_B	Fusobacterium_nucleatum_subsp._nucleatum_ATCC_25586_AAL93849	Bacteria	Atribacteria	Candidatus Atribacteria bacterium RBG 19FT COMBO 35 14
V_B	Streptobacillus_moniliformis_DSM_12112_ACZ00742	Archaea	Aigarchaeota	Candidatus Caldiarchaeum subterraneum
V_B	Streptococcus_pyogenes_M1_GAS_AAK33258	Bacteria	unclassified Bacteria	Candidatus Edwardsbacteria bacterium RifOxyC12 full 54 24
V_B	Candidatus_Acetothermus_autotrophicum_BAL59641	Archaea	Heimdallarchaeota	Candidatus Heimdallarchaeota archaeon LC 3
V_B	Caldisericum_exile_AZM16c01_BAL81283	Bacteria	Latescibacteria	Candidatus Latescibacteria bacterium 4484 107
V_B	Caldisericum_sp._CG2_30_36_11_OIP12905.1	Bacteria	Candidatus Latescibacteria	Candidatus Latescibacteria bacterium 4484 7
V_B	candidate_division_WOR-1_bacterium_RIFOXYA12_FULL_43_27_OGC05191.1	Bacteria	Fibrobacteres/Acidobacteria	Candidatus Marinimicrobia bacterium CG08
V_B	candidate_division_WOR-1_bacterium_RIFOXYC2_FULL_41_25_OGC34888.1	Archaea	Euryarchaeota	Candidatus Methanohalarchaeum thermophilum
V_B	Candidatus_Saganbacteria_bacterium_CG08_land_8_20_14_0_20_45_16_PIS30894.1	Archaea	Micrarchaeota	Candidatus Micrarchaeota archaeon CG 4
V_B	Candidatus_Nealsonbacteria_bacterium_RIFOXYB1_FULL_40_15_OGZ27423.1	Archaea	Micrarchaeota	Candidatus Micrarchaeota archaeon CG1
V_B	Dictyoglomus_turgidum_DSM_6724_ACK42774	Archaea	Micrarchaeota	Candidatus Micrarchaeota archaeon Mia14
V_B	Acidilobus_saccharovorans_345-15_ADL18827	Archaea	Micrarchaeota	Candidatus Micrarchaeum acidiphilum ARMAN-2
V_B	Caldisphaera_lagunensis_DSM_15908_AFZ70922	Bacteria	unclassified Bacteria	Candidatus Moduliflexus flocculans (bacterium UASB14)
V_B	Aeropyrum_pernix_K1_BAA79360	Bacteria	unclassified Bacteria	Candidatus Nealsonbacteria bacterium
V_B	Hyperthermus_butylicus_WP_048061765	Archaea	Thaumarchaeota	Candidatus Nitrosoarchaeum koreensis
V_B	Pyrodictium_delaneyi_WP_055410825	Archaea	Thaumarchaeota	Candidatus Nitrosopelagicus brevis
V_B	Pyrolobus_fumarii_WP_048192620	Archaea	Thaumarchaeota	Candidatus Nitrosopumilus salaria
V_B	Metallosphaera_yellowstonensis_MK1_EHP68783	Archaea	Thaumarchaeota	Candidatus Nitrosotalea devanaterra
V_B	Sulfolobus_tokodaii_str._7_BAK54573	Archaea	Thaumarchaeota	Candidatus Nitrosotenuis cloacae
V_B	Ignicoccus_hospitalis_KIN4/I_A8AAA9	Archaea	Odinarchaeota	Candidatus Odinarchaeota archaeon
V_B	Pyrodictium_delaneyi_ALL01377	Archaea	Parvarchaeota	Candidatus Parvarchaeum acidiphilum ARMAN-4
V_B	Aciduliprofundum_boonei_T469_ADD09069	Archaea	Parvarchaeota	Candidatus Parvarchaeum acidophilus ARMAN-5
V_B	Methanobacterium_formicicum_DSM_3637_EKF85768	Bacteria	unclassified Bacteria	Candidatus Ratteibacteria bacterium CG15
V_B	Methanothermus_fervidus_DSM_2088_ADP78037	Bacteria	unclassified Bacteria	Candidatus Ratteibacteria bacterium CG23

V_B	Methanobrevibacter_smithii_ATCC_35061_A5UKB1	Bacteria	unclassified Bacteria	Candidatus Saganbacteria bacterium
V_B	Methanocaldococcus_jannaschii_DSM_2661_AAB98199	Bacteria	unclassified Bacteria	Candidatus Stahlbacteria bacterium
V_B	Methanococcus_maripaludis_S2_CAF30601	Archaea	Thorarchaeota	Candidatus Thorarchaeota archaeon AB 25
V_B	Pyrococcus_furiosus_DSM_3638_AAL80307	Archaea	Thorarchaeota	Candidatus Thorarchaeota archaeon SMTZ1-45
V_B	Pyrococcus_horikoshii_WP_048053618	Archaea	Thorarchaeota	Candidatus Thorarchaeota archaeon SMTZ1-83
V_B	Thermococcus_WP_048053740	Bacteria	unclassified Bacteria	Candidatus Vecturithrix granuli (bacterium UASB270)
V_B	Hadesarchaea_archaeon_YNP_45_KUO42039	Archaea	Thaumarchaeota	Cenarchaeum symbiosum A
V_B	Candidatus_Methanohalarchaeum_thermophilum_OKY78118	Bacteria	Deinococcus-Thermus	Deinococcus radiodurans
V_B	Methanonatronarchaeum_thermophilum_OUJ19124	Archaea	Crenarchaeota	Desulfurococcus mucosus DSM 2162
V_B	Candidatus_Odinarchaeota_archaeon_LCB_4_OLS17679	Bacteria	Dictyoglomi	Dictyoglomus turgidum
V_B	Candidatus_Altiarchaeales_archaeon_ex4484_2_OYT54115	Archaea	Euryarchaeota	Ferroplasma acidarmanus fer1
V_B	Candidatus_Altiarchaeales_archaeon_IMC4_ODS43116	Bacteria	Firmicutes	Finegoldia magna ATCC 29328
V_B	Candidatus_Thorarchaeota_archaeon_AB_25_OLS31427	Bacteria	Fusobacteria	Fusobacterium nucleatum
V_B	Candidatus_Thorarchaeota_archaeon_SMTZ1-45_KXH73442	Archaea	Euryarchaeota	Hadesarchaea archaeon YNP 45
V_B	Candidatus_Thorarchaeota_archaeon_SMTZ1-83_KXH77545	Bacteria	Fibrobacteres/Acidobacteria	Holophaga foetida
V_B	Candidatus_Micrarchaeota_archaeon_CG1_02_47_40_OIO21946.1	Archaea	Crenarchaeota	Hyperthermus butylicus
V_B	Candidatus_Micrarchaeota_archaeon_CG_4_10_14_0_2_um_filter_60_11_PIZ91074.1	Archaea	Crenarchaeota	Ignicoccus hospitalis KIN4/I
V_B	Candidatus_Micrarchaeota_archaeon_Mia14_ASI13682.1	Bacteria	Deinococcus-Thermus	Meiothermus ruber
V_B	Candidatus_Micrarchaeum_acidiphilum_ARMAN-2_EET90348	Archaea	Crenarchaeota	Metallosphaera yellowstonensis MK1
V_B	Candidatus_Parvarchaeum_acidiphilum_ARMAN-4_EEZ93164	Archaea	Euryarchaeota	Methanobacterium formicicum DSM 3637
V_B	Candidatus_Parvarchaeum_acidophilus_ARMAN-5_EFD92467	Archaea	Euryarchaeota	Methanobrevibacter smithii ATCC 35061
V_B	Candidatus_Nitrosotalea_devanaterra_CUR52765	Archaea	Euryarchaeota	Methanocaldococcus jannaschii
V_B	Ferroplasma_acidarmanus_fer1_AGO60915	Archaea	Euryarchaeota	Methanocella paludicola SANAE
V_B	Picrophilus_torridus_DSM_9790_AAT43074	Archaea	Euryarchaeota	Methanococcus maripaludis S2
V_B	Thermoplasma_acidophilum_DSM_1728_Q9HM64	Archaea	Euryarchaeota	Methanonatronarchaeum thermophilum
V_B	Thermoplasma_volcanium_GSS1_BAB59194	Archaea	Euryarchaeota	Methanothermus fervidus
V_B	Candidatus_Moduliflexus_flocculans_GAK54040.1	Eukarya	Heterolobosea	Naegleria gruberi
V_B	Candidatus_Vecturithrix_granuli_GAK59871.1	Archaea	Thaumarchaeota	Nitrosopumilus maritimus SCM1
V_B	Candidatus_Caldiarchaeum_subterraneum_BAE03291	Archaea	Thaumarchaeota	Nitrososphaera viennensis EN76
V_B	Candidatus_Nitrosoarchaeum_koreensis_MY1_EGP94528	Archaea	Euryarchaeota	Picrophilus torridus
V_B	Nitrosopumilus_maritimus_SCM1_A9A2Q9	Bacteria	Synergistetes	Pyramidobacter piscolens
V_B	Candidatus_Nitrosopumilus_salaria_BD31_EIJ66235.1	Archaea	Crenarchaeota	Pyrobaculum aerophilum
V_B	Candidatus_Nitrosotenuis_cloacae_AJZ75932	Archaea	Euryarchaeota	Pyrococcus furiosus
V_B	Thaumarchaeota_archaeon_MY2_WP_042684210	Archaea	Euryarchaeota	Pyrococcus horikoshii OT3
V_B	Candidatus_Nitrosopelagicus_brevis_AJA92453	Archaea	Crenarchaeota	Pyrodictium delaneyi
V_B	Cenarchaeum_symbiosum_A_A0RXK0	Archaea	Crenarchaeota	Pyrodictium delaneyi
V_B	Nitrososphaera_viennensis_EN76_AIC16551	Archaea	Crenarchaeota	Pyrolobus fumarii
V_B	Methanocella_paludicola_SANAE_BAI62363	Archaea	Crenarchaeota	Staphylothermus marinus F1
V_B	Desulfurococcus_mucosus_DSM_2162_ADV64399	Bacteria	Fusobacteria	Streptobacillus moniliformis
V_B	Staphylothermus_marinus_F1_A3DNQ5	Bacteria	Bacillota	Streptococcus pyogenes
V_B	Thermosphaera_aggregans_DSM_11486_ADG90360	Archaea	Crenarchaeota	Sulfolobus tokodaii
V_B	Candidatus_Heimdallarchaeota_archaeon_LC_3_OLS22921	Bacteria	Synergistetes	Synergistes jonesii
V_B	Candidatus_Latescibacteria_bacterium_4484_7_OQX86193	Bacteria	Synergistetes	Synergistes jonesii
V_B	Candidatus_Marinimicrobia_bacterium_CG08_land_8_20_14_0_20_45_22_PIS28244.1	Archaea	Thaumarchaeota	Thaumarchaeota archaeon MY2
V_B	Holophaga_foetida_WP_005035427	Eukarya	Apsozoa	Thecamonas trahens ATCC 50062
V_B	Candidatus_Latescibacteria_bacterium_4484_107_OPX22522	Bacteria	Synergistetes	Thermanaerovibrio acidaminovorans
V_B	Synergistes_jonesii_KEJ93510	Bacteria	Synergistetes	Thermanaerovibrio acidaminovorans
V_B	Thermanaerovibrio_acidaminovorans_DSM_6589_ACZ18752	Archaea	Euryarchaeota	Thermococcus
V_B	Naegleria_gruberi_EFC47722	Archaea	Euryarchaeota	Thermoplasma acidophilum DSM 1728

V_B	Thecamonas_trahens_ATCC_50062_KNC52343	Archaea	Euryarchaeota	Thermoplasma volcanium GSS1
V_B	Caldivirga_maquilingensis_IC-167_ABW01188	Archaea	Crenarchaeota	Thermosphaera aggregans
V_B	Vulcanisaeta_moutnovskia_768-28_ADY01141	Bacteria	Deinococcus-Thermus	Thermus thermophilus HB27
V_B	Pyrobaculum_aerophilum_str._IM2_AAL63284	Archaea	Crenarchaeota	Vulcanisaeta moutnovskia768-28
