# Supplementary material for: Functional and structural characterization of F1 ‐ATPase with common ancestral core domains in stator ring
Source: Protein Sci. 2025 Oct 23;34(11):e70345. doi: 10.1002/pro.70345 (PMC12550136; doi:10.1002/pro.70345)
Supplement: Supplementary file 18 — Data S18: Supplementary information. [file PRO-34-e70345-s004.docx]

**Supporting Information**

**Functional and Structural Characterization of F_1_-ATPase with common ancestral core domains in stator ring**

Aya K. Suzuki^1^, Ryutaro Furukawa^2^, Meghna Sobti^3,4^, Simon H. J. Brown^5^, Alastair G. Stewart^3,4^, Satoshi Akanuma^6^, Hiroshi Ueno^2*^, Hiroyuki Noji^2,7*^

^1^ Department of Life Sciences, Graduate School of Arts and Sciences, The University of Tokyo, Tokyo, Japan.

^2^ Department of Applied Chemistry, Graduate School of Engineering, The University of Tokyo, Tokyo, Japan.

, ^3^Molecular, Structural and Computational Biology Division, The Victor Chang Cardiac Research Institute, NSW, Australia.

, ^4^ St Vincent’s Clinical School, Faculty of Medicine, UNSW Sydney, NSW, Australia.

, ^5^School of Science, Molecular Horizons, and the Australian Research Council Centre for Cryo-electron Microscopy of Membrane Proteins, University of Wollongong, Wollongong, NSW, Australia.

, ^6^Faculty of Human Sciences, Waseda University, Saitama, Japan.

, ^7^ Research Institute of Planetary Health (RIPH), The University of Tokyo, Tokyo, Japan,

^*^Corresponding authors: hueno@g.ecc.u-tokyo.ac.jp and hnoji@g.ecc.u-tokyo.ac.jp


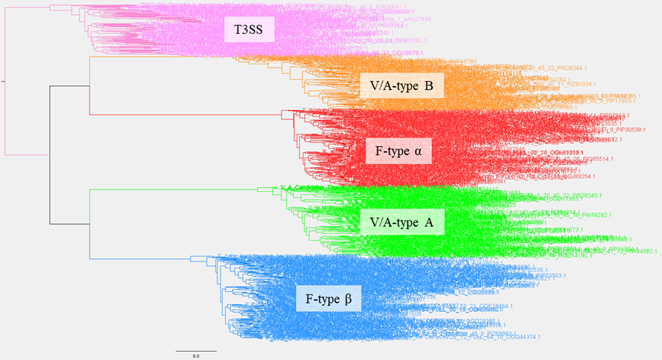
Fig. S1. Phylogenetic tree of catalytic/non-catalytic subunits of rotary ATPases

The phylogenetic tree was constructed using IQ-TREE based on a dataset of 617 sequences, including 94 T3SS ATPase FliI sequences, 142 F-type ATPase α-subunit sequences, 153 β-subunit sequences, 128 V-type ATPase A-subunit sequences, and 100 B-subunit sequences.


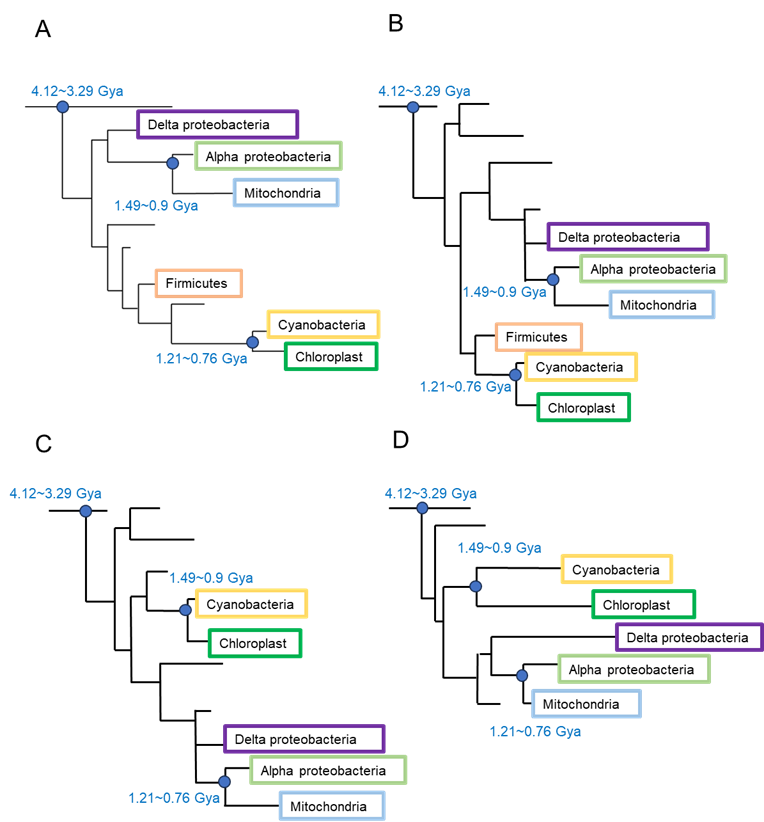


Fig. S2. Phylogenetic Tree Topology Comparison

To compare tree topologies, we manually extracted the branching patterns of shared phyla. Branch lengths indicate evolutionary rates. Estimated divergence times are annotated at corresponding nodes based on the time estimates from a previous study^[1]^. **(A-B)** α subunit. **A.** Previous study^[1]^, **B.** This study. **(C-D)** β subunit. **C.** Previous study^[1]^, **D.** This study.


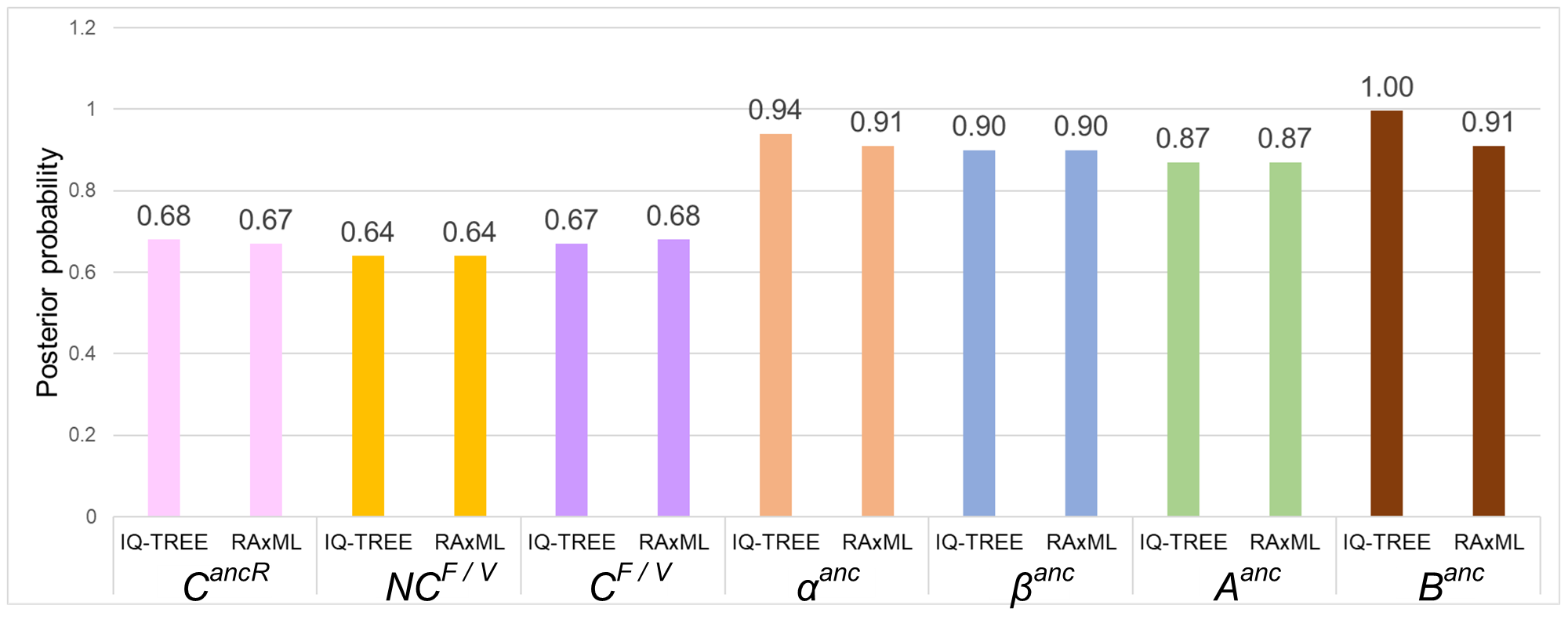


Fig. S3. Evaluation of Posterior Probability Values

Posterior probability values of ancestral sequences reconstructed with IQ-tree-based or RAxML-based phylogenetic trees are presented for each ancestral subunit.


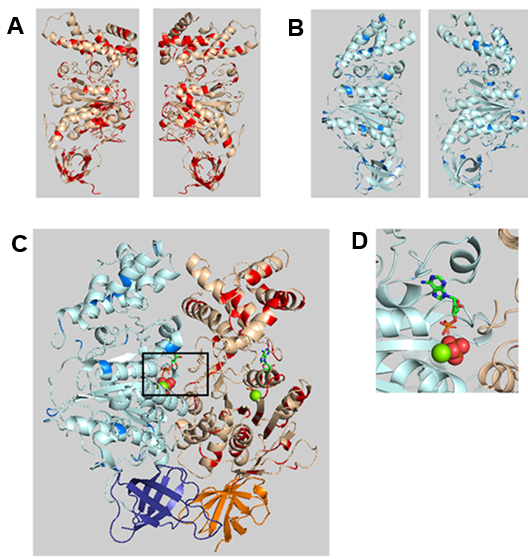


Fig. S4. Comparison of IQ-TREE- and RAxML-based sequence reconstruction

The sequences of the ancestral subunit inferred with IQ-TREE- and RAxML-based phylogenic trees were compared and the sequence differences were mapped on the subunit structure of TF_1_ The structural information was obtained from the PDB (PDB ID:4XD7). **A.** Structural map of the common ancestral α-subunit (*α^anc^*). The sequence differences are highlighted in red, while conserved regions are shown in cream. **B.** Structural map of the common ancestral β-subunit (*β^anc^*). The sequence differences are highlighted in red, while conserved regions are shown in cream. **C.** Structural map of the α-β interface. The N-terminal domains of the α- or β-subunit are depicted with dark blue and orange. **D.** A magnified view of the substrate-binding site. No amino acid difference was observed between the two sequences.


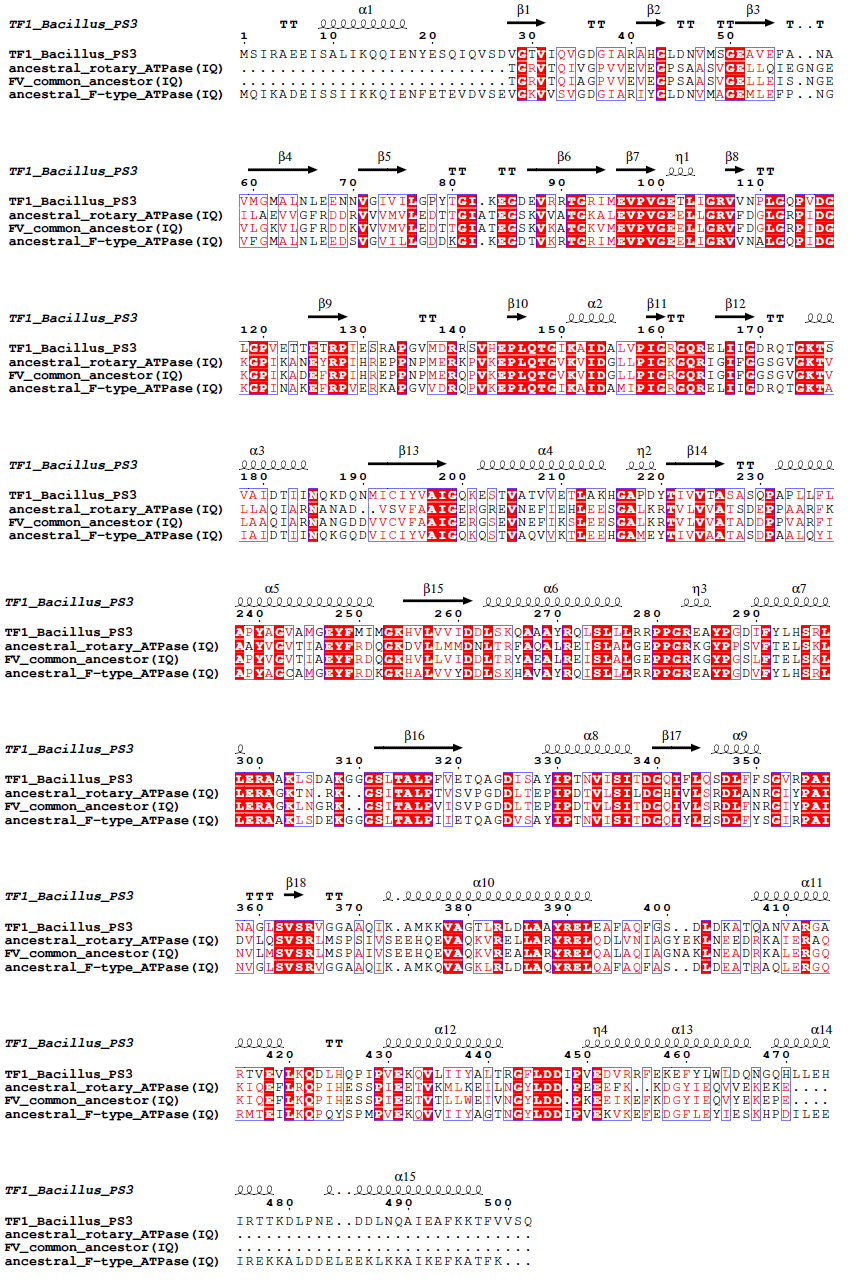


Fig. S5. MSA of Ancestral and Extant F-type ATPase α Subunit Sequences (IQ-TREE)

The multiple sequence alignment (MSA) includes the sequence and secondary structure of the α subunit from the extant TF_1_ ATPase, along with ancestral sequences reconstructed based on the IQ-TREE phylogenetic tree. These ancestral sequences comprise the ancestral rotary ATPase (*C*^ancR^), which represents the common ancestor of the F-type ATPase α/β subunits and the V-type ATPase A/B subunits, the F/V common ancestor (*NC* ^F/V^), which is the common ancestor of the non-catalytic subunits F-type ATPase α and V-type ATPase B, and the ancestral F-type ATPase (*α*^anc^), which is the common ancestor of the F-type ATPase α subunits. Conserved sequence regions are highlighted in red with white text, while groups of amino acid residues with similar properties, such as hydrophobic/hydrophilic or acidic/basic characteristics, are enclosed in blue boxes. Secondary structure annotations include α-helices (α), β-strands (β), 310 helices (η), and loop structures causing abrupt directional changes in the polypeptide chain (TT), with structural information obtained from the PDB (PDB ID: 6N2Y). The sequence region corresponding to the N-terminal structural foundation involved in complex formation in the hybrid F_1_ with the ancestral core domains is shaded in orange.


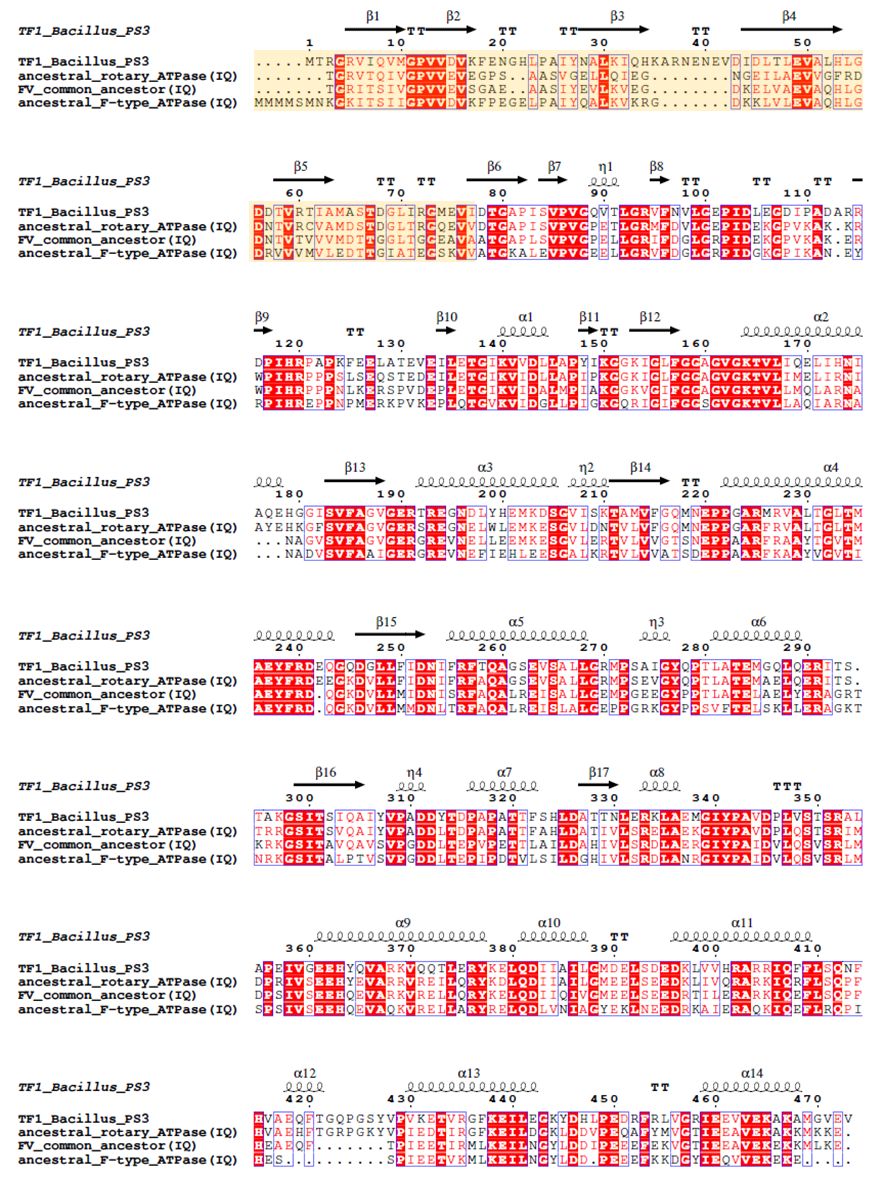


Fig. S6. MSA of Ancestral and Extant F-type ATPase β Subunit Sequences (IQ-TREE)

The multiple sequence alignment (MSA) includes the sequence and secondary structure of the β subunit from the extant TF_1_ ATPase, along with ancestral sequences reconstructed based on the IQ-TREE phylogenetic tree. These ancestral sequences comprise the ancestral rotary ATPase (*C*^ancR^), which represents the common ancestor of the F-type ATPase α/β subunits and the V-type ATPase A/B subunits, the F/V common ancestor (*C*^F/V^), which is the common ancestor of the catalytic subunits F-type ATPase β and V-type ATPase A, and the ancestral F-type ATPase (*β*^anc^), which is the common ancestor of the F-type ATPase β subunits. Conserved sequence regions are highlighted in red with white text, while groups of amino acid residues with similar properties, such as hydrophobic/hydrophilic or acidic/basic characteristics, are enclosed in blue boxes. Secondary structure annotations include α-helices (α), β-strands (β), 310 helices (η), and loop structures causing abrupt directional changes in the polypeptide chain (TT), with structural information obtained from the PDB (PDB ID: 6N2Y). The sequence region corresponding to the N-terminal structural foundation involved in complex formation in the hybrid F_1_ with the ancestral core domains is shaded in orange.


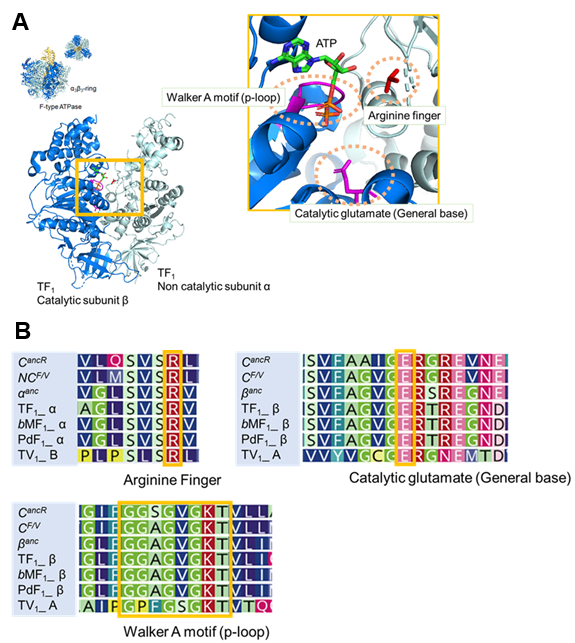


Fig. S7. Catalytically critical residues and motifs of ancestral sequences

**A.** Structural location of catalytically critical residues and motifs. **B.** Sequence alignment of the common ancestral sequences and extant sequences. Orange boxes show the catalytic residues and motifs.


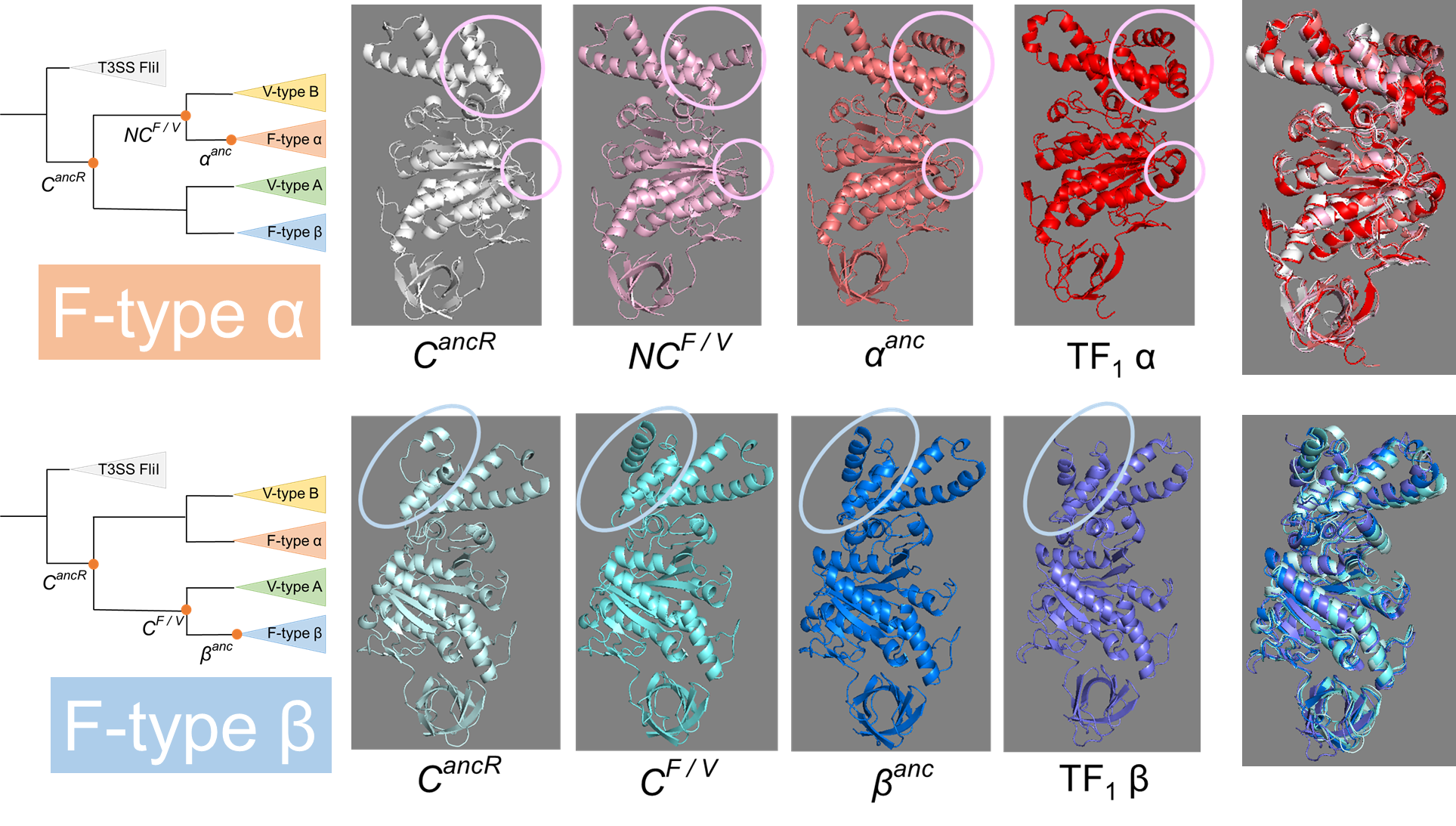


Fig. S8. Evolutionary Trajectories of F-type ATPase Subunits

The evolutionary transitions of each subunit from the common ancestor of F-type and V-type ATPases are depicted by arranging ancestral structures sequentially from left to right, corresponding to upstream nodes in the phylogenetic tree. On the right, the structural superpositions of each subunit are displayed. Circular markers indicate regions where significant structural changes were observed. The structure of TF_1_ is referenced as a representative of extant species. In the figure, the top represents the C-terminal side, while the bottom corresponds to the N-terminal side. Across all ancestral forms and the extant species, the fundamental structural elements remain conserved, including the α-helical structure at the C-terminal region, the β-sheet structure in the central region, and the β-barrel structure at the N-terminal region. The structures of the ancestral types were predicted using AlphaFold2^[3]^, a deep learning-based algorithm developed by DeepMind. The model was implemented using the open-source version of AlphaFold2 with default parameters.


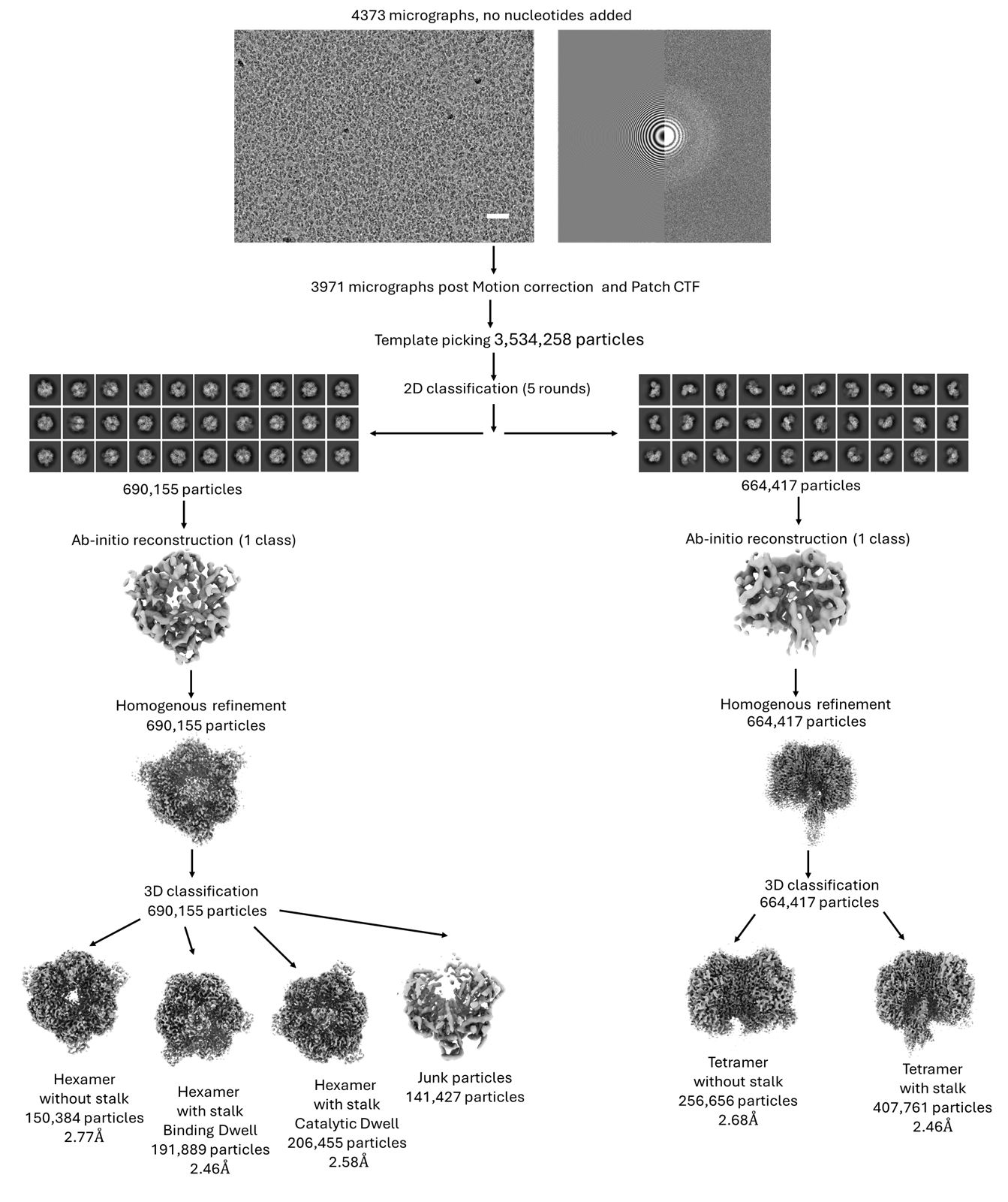


Fig. S9. Cryo-EM data collection and data processing flowchart

Micrographs with white scale bar equivalent to 50 nm. 2D class averages and 3D classification were used to sort the data into sub-states.


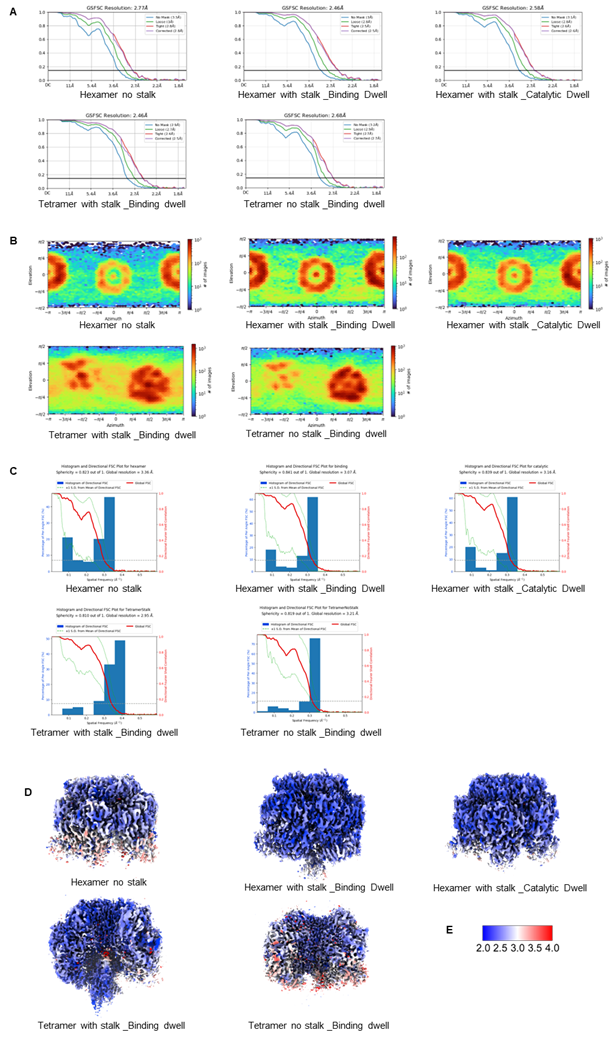


Fig. S10. 3D FSC Curves and Local Resolution Estimates

**A.** Gold standard Fourier shell correlation (GSFSC) curves from CryoSPARC. **B.** Viewing direction distribution plot. **C.** Histogram and direct FSC Plot (3D FSC). **D.**Local resolution estimate calculated in cryoSPARC. **E.** Local resolution estimate scale.


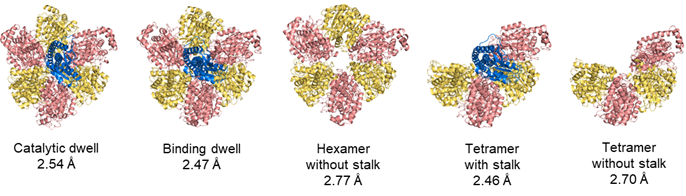


Fig. S11. Structures of F_1_^anc_core^ obtained in this study

The structural representation of the common ancestral F_1_-ATPase is shown with distinct color coding for each subunit: the α-subunit is depicted in pink, the β-subunit in cream, and the γ-subunit in light blue.


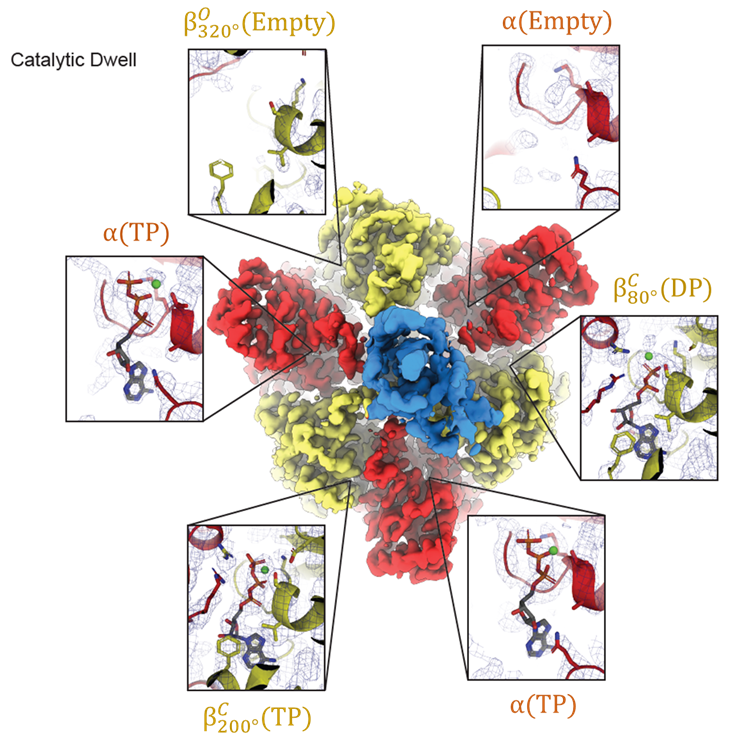


Fig. S12. Close-up Views of the Nucleotide Binding Sites (Catalytic dwell)

The cryo-EM maps are shown along with close-up views of each nucleotide binding site. Cryo-EM map is shown as mesh at 4 σ in all close-up panels. At the ~80° and 320° rotational states, the β subunits of F_1_^anc_core^ were bound to ADP and no nucleotide, respectively, whereas in TF_1_ the equivalent sites carried ATP and ADP. These differences in nucleotide occupancy likely arise from the absence of exogenous nucleotides during sample preparation.


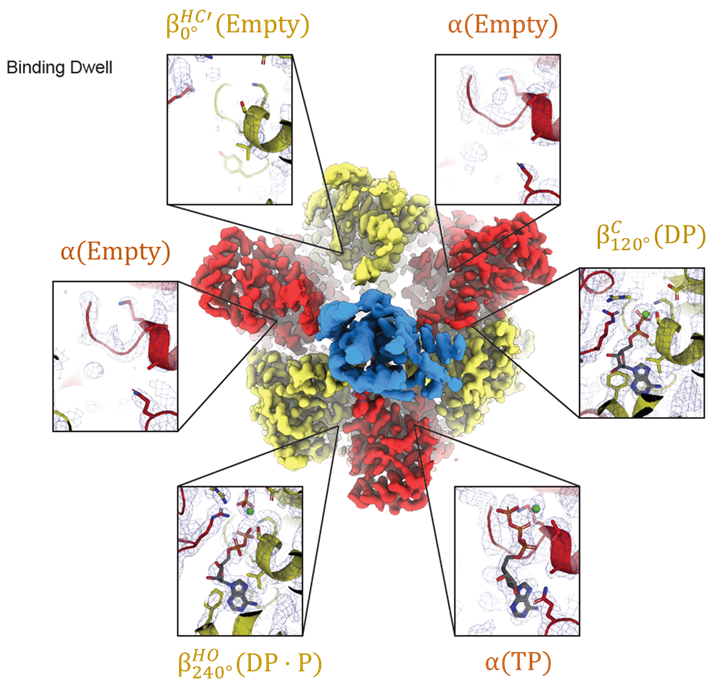


Fig. S13. Close-up Views of the Nucleotide Binding Sites (Binding dwell)

The cryo-EM maps are shown along with close-up views of each nucleotide binding site. Cryo-EM map is shown as mesh at 4 σ in all close-up panels. At the 120° rotational states, the β subunits of F_1_^anc_core^ were bound to ADP, whereas in TF_1_ the equivalent sites carried ATP. This difference in nucleotide occupancy likely arises from the absence of exogenous nucleotides during sample preparation.


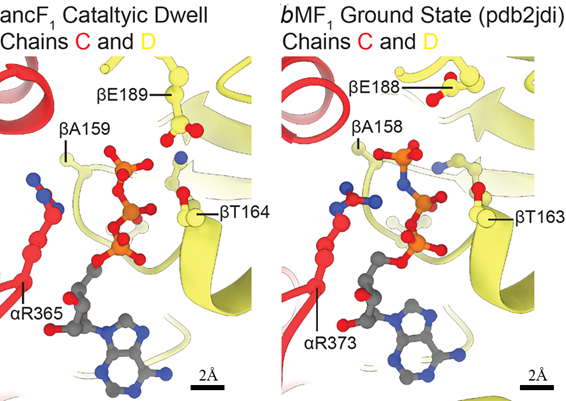


Fig. S14. Direct comparison between F_1_^anc_core^ and the bovine mitochondrial F_1_ ground state

Comparison of the binding site between $\beta_{200^{\circ}}^{C}\left( \mathrm{TP} \right)$ and $\alpha\left( \mathrm{TP} \right)$ for F_1_^anc_core^ in catalytic state(left) and bovine mitochondrial F_1_ in ground state (right). The alpha subunits are colored red, and beta subunits are colored yellow. Residues are labelled to allow for comparison of p-loop (from βA159 to βT164 for F_1_^anc_core^, and from βA159 to βT164 for *b*MF_1_), arginine finger (αR365 for F_1_^anc_core^, and αR363 for *b*MF_1_) and catalytic glutamate (βE189 for F_1_^anc_core^, and βE188 for *b*MF_1_). Although the catalytic glutamate and arginine finger are in slightly different conformations, this is likely just due to the limited resolution of the structural data.


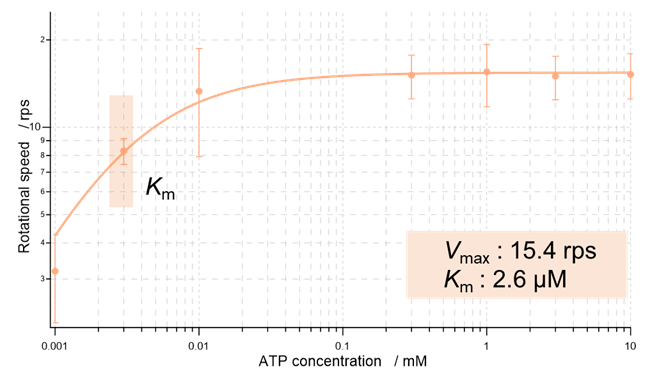
Fig. S15. Michaelis-Menten Curve of F_1_^anc_core^ for ATP

The mean rotational velocity of at least three particles was plotted for each ATP concentration and fitted using the Michaelis-Menten equation. Error bars represent the standard deviation (SD). The fitting analysis estimated a maximum velocity (*V*_max_) of 15.4 rps and a Michaelis constant (*K*_m_) of 2.6 μM.


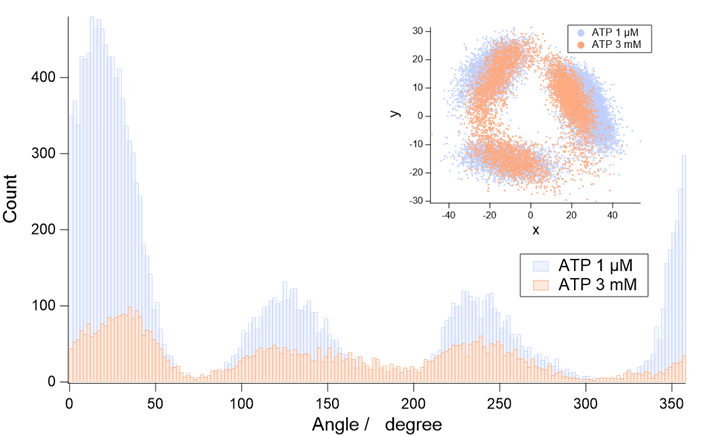
Fig. S16. Solution Exchange Between 3 mM ATP and 1 μM ATP

Angle histograms before and after solution exchange. The light purple histogram represents the low ATP concentration condition (1 μM ATP), while the light orange histogram corresponds to the high ATP concentration condition (3 mM ATP). The upper right panel displays x–y plots for each ATP concentration, with color coding consistent with the angle histograms.


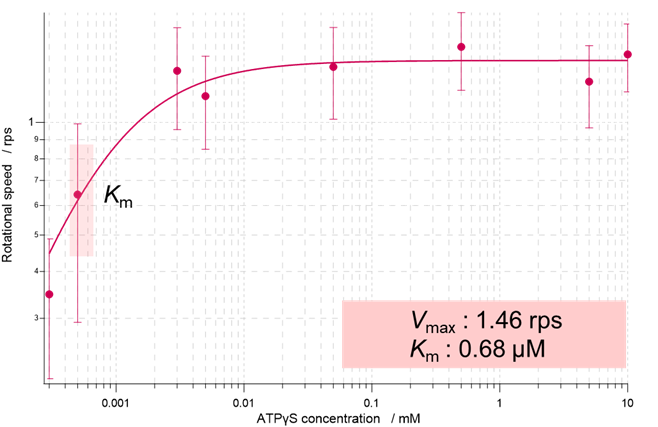


Fig. S17. Michaelis-Menten Curve of F_1_^anc_core^ for ATPγS

The mean rotational velocity of at least three particles was plotted for each ATPγS concentration and fitted using the Michaelis-Menten equation. Error bars represent the standard deviation (SD). The fitting analysis estimated a maximum velocity (*V*_max_) of 1.46 rps and a Michaelis constant (*K*_m_) of 0.68 μM.


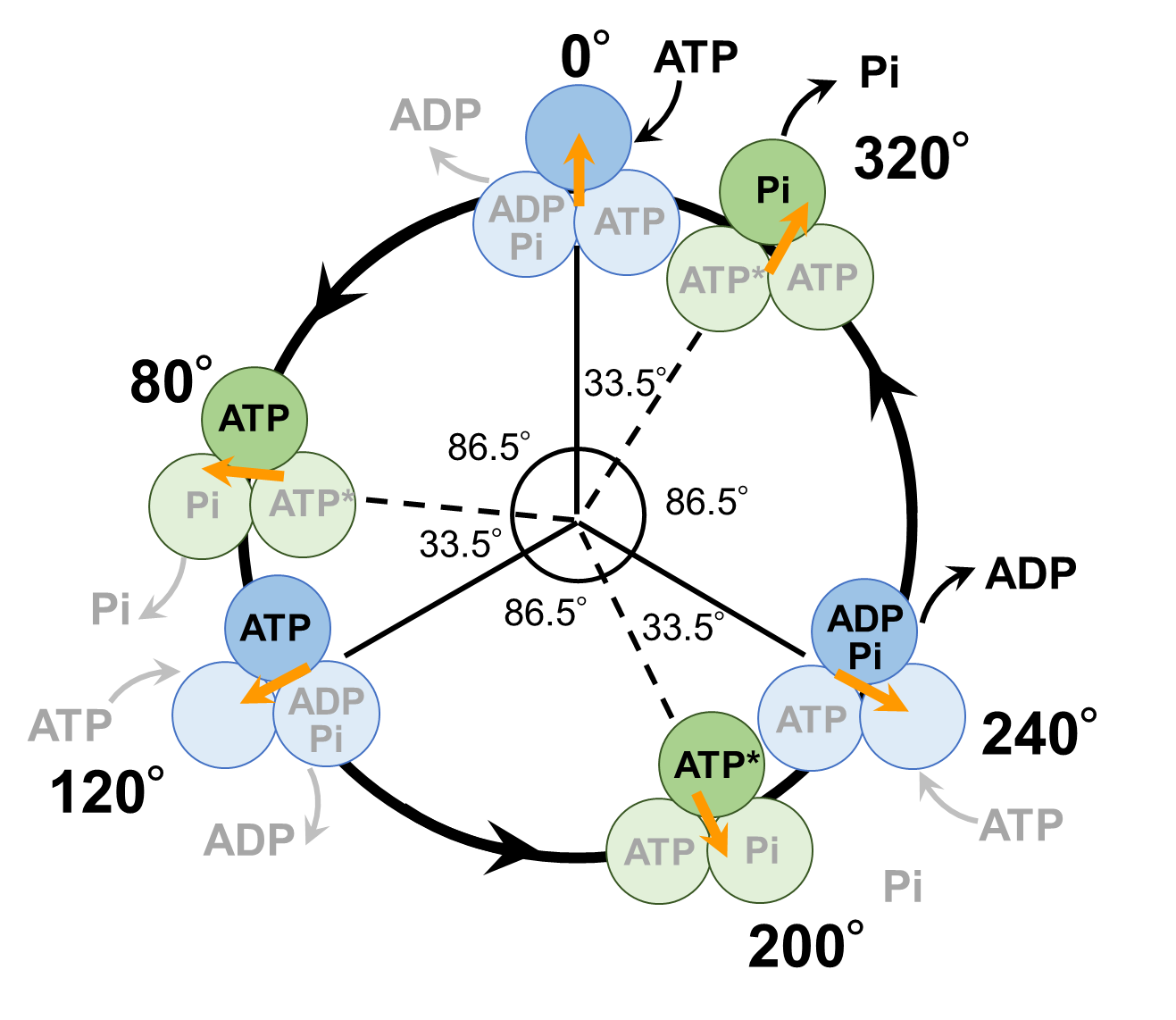
Fig. S18. Rotary Catalytic Mechanism of Hybrid F_1_ with the Ancestral Core Domains

The three circles represent the three β subunits of the hexameric ring, with the nucleotides bound at each β subunit’s binding site indicated inside the circles. ATP* denotes the activated state of ATP prior to hydrolysis. The orange arrow indicates the rotation of the γ subunit. The blue and light blue markers represent the binding dwell positions, while the green and light green markers indicate the catalytic dwell positions, where rotation pauses.

Due to the significantly faster reaction kinetics occurring at the catalytic dwell compared to the binding dwell, the rotational angles of the binding dwell are represented by solid lines, whereas those of the catalytic dwell are depicted by dashed lines. The difference in rotational angles between the binding dwell and catalytic dwell corresponds to the angular shift revealed by cryo-EM structural analysis.

Table. S1. Sequence Identity Between Ancestral and Extant sequences

|  | *C^ancR^* | | *NC^F / V^* | | *C^F / V^* | |
| --- | --- | --- | --- | --- | --- | --- |
|  | IQ-TREE | RAxML | IQ-TREE | RAxML | IQ-TREE | RAxML |
| TF_1__α | 37 % | 35 % | 43 % | 39 % |  |  |
| TF_1__β | 46 % | 46 % |  |  | 58 % | 56 % |
| *b*MF_1__α | 36 % | 35 % | 43 % | 40 % |  |  |
| *b*MF_1__β | 43 % | 45 % |  |  | 58 % | 56 % |
| TtV_1__A | 34 % | 33 % |  |  | 40 % | 40 % |
| TtV_1__B | 36 % | 37 % | 39 % | 41 % |  |  |

Table. S2. Proportion of Particles with and without nucleotide

|  | AMP-PNP | | w/o nucleotide | |
| --- | --- | --- | --- | --- |
|  | Number of particles | % | Number of particles | % |
| All particles | 1,753,764 |  | 1,357,417 |  |
| Hexamer w/ stalk | 97,557 | 5.6 | 398,344 | 29.3% |
| Hexamer w/o stalk | 114,081 | 6.5 | 150,384 | 11% |
| Tetramer w/ stalk | 225,450 | 12.9 | 256,656 | 18.9% |
| Tetramer w/o stalk | 255,786 | 14.6 | 407,761 | 30% |
| Unclassified | 1,060,890 | 60.5 | 144,272 | 10.6% |

Table. S3. *Q*_10_ Factor of F_1_^anc_core^

| Temperature | w/ LDAO | w/o LDAO |
| --- | --- | --- |
| 20℃ - 30℃ | 1.33 | 2.91 |
| 25℃ - 35℃ | 1.60 | 1.89 |
| 30℃ - 40℃ | 1.63 | 1.79 |
| Average | 1.52 | 2.20 |

The ATPase activity of F_1_^anc_core^ was measured at various solution temperatures to estimate the *Q*_10_ factor, which represents the fold increase in ATPase activity upon a 10°C rise in temperature. ATPase activity measurements were conducted twice at each temperature, and the average values were used to estimate the *Q*_10_ factor.

Table. S4. Cryo-EM data collection, refinement and validation statistics

#### Reference

1. Mahendrarajah TA, Moody ERR, Schrempf D, Szánthó LL, Dombrowski N, Davín AA, Pisani D, Donoghue PCJ, Szöllősi GJ, Williams TA, Spang A (2023) ATP synthase evolution on a cross-braced dated tree of life. *Nat Commun* **14**:7456.
2. Punjani A, Rubinstein JL, Fleet DJ, Brubaker MA (2017) cryoSPARC: algorithms for rapid unsupervised cryo-EM structure determination. *Nat Methods* **14**:290–296.
3. Jumper J, Evans R, Pritzel A, Green T, *et. al.*, (2021) Highly accurate protein structure prediction with AlphaFold. *Nature* 596:583–589.
